# A Simple Access to Highly Active Heterogeneous Hydrogenation Catalysts

**DOI:** 10.3390/molecules31152601

**Published:** 2026-07-25

**Authors:** Sebastian W. Simon, Laura La Placa, Fabian Casalino, Artur Felske, Tom Milbert, Anna Borysenko, Tobias Simon, Stefan Lach, Johannes L’huillier, Christiane Ziegler, Wolfgang Kleist, Werner R. Thiel

**Affiliations:** 1 Fachbereich Chemie, RPTU Kaiserslautern Landau, Erwin Schrödinger-Str. 54, 67663 Kaiserslautern, Germany; 2 Institut für Oberflächen- und Schichttechnik GmbH, IFOS GmbH, Trippstadter Straße 120, 67663 Kaiserslautern, Germany; 3 Fachbereich Physik and Research Center OPTIMAS, RPTU Kaiserslautern Landau, Erwin Schrödinger-Str. 56, 67663 Kaiserslautern, Germany

**Keywords:** hydrogenation, phosphine ligand, palladium, rhodium, silica, SBA-15

## Abstract

This paper describes the synthesis, characterization and application in hydrogenation catalysis of triphenylphosphine-stabilized palladium and rhodium species on SBA-15. The materials were achieved by synthesizing a triethoxysilyl-functionalized triphenylphosphine ligand through the reaction of (2-chloromethylphenyl)diphenylphosphine and (3-mercaptopropyl)triethoxysilane. Treatment of this ligand with the appropriate palladium(II) and rhodium(I) complexes produced the corresponding transition metal complexes, which were then immobilized on the support material. The final catalysts were obtained by treating these precursor materials with dihydrogen at elevated temperatures. These catalysts are highly active in the hydrogenation of a wide range of olefins under mild conditions. XPS studies on one of the palladium catalysts reveal that only a part of the metal is reduced, with almost no further reduction of the remaining palladium(II) species observed after tenfold recycling and reuse of the material.

## 1. Introduction

Catalytic hydrogenation—that is, the addition of hydrogen to organic compounds containing multiple bonds (e.g., olefins, alkynes, carbonyl compounds, carboxylic acids and esters, imines, amides, nitriles, nitro compounds, etc.)—is a fundamental reaction in chemical synthesis. It dates to 1897, when Sabatier and Sanderens first described the catalytic hydrogenation of ethene using reduced nickel oxide in a heterogeneous reaction [1]. Since then, a multitude of homogeneous and heterogeneous hydrogenation catalysts have been developed. The clear advantage of heterogeneous hydrogenation catalysts is that they can be easily removed from the reaction mixture for reuse, and their solid-state nature makes them suitable for applications in flow systems.

This triggered investigations on the immobilization of highly reactive homogeneous catalysts on inert support materials [2]. As early as 1971, Grubbs et al. reported on a novel catalyst material that they obtained by immobilizing a rhodium(I) complex similar to the Wilkinson catalyst on polystyrene beads [3]. However, since cross-linked polymers exhibit varying swelling behavior depending on the composition of the reaction mixture, research has increasingly focused on inert metal oxides as catalyst support materials.

In 1975, Howell et al. pioneered the use of silica gels for the immobilization of catalytically active transition metal complexes by modifying the support with various phosphorus, nitrogen, sulfur, and oxygen donor ligands using the post-grafting method [4,5]. In their work, the authors demonstrated that it is more advantageous to first synthesize trialkylsilyl-functionalized complexes and then immobilize them than to immobilize the linker group with the ligand in the first step and subsequently incorporate the transition metal center. Since then, a large number of other catalytic complexes have been immobilized [6,7,8,9,10,11,12,13,14,15,16]. These materials are often, but not always, resistant to the leaching of the catalytically active metal species.

As the basic principles of the heterogenization of catalytically active complexes are now well understood, the focus of scientific interest has shifted to the topics of selectivity and activity. Thermodynamically, it is in principle possible to carry out hydrogenation reactions without an external heat input and at atmospheric pressure.

The mesoporous, ordered silica gel SBA-15 [17,18,19] is particularly well-suited for this purpose, as it is characterized not only by a high specific surface area (up to 1000 m^2^/g) but also by pore diameters of 4–30 nm [20], which facilitates diffusion processes. In addition, the material exhibits micropores (<2 nm) formed by the polyethylene glycol chains of the P123 template. These chains are predominantly embedded in the silica framework during the formation of the material and leave behind micropore channels upon removal [21]. SiOH groups on the pore surface allow the immobilization of functional groups via alkoxysilanes [22,23,24,25].

Some time ago, we published the synthesis of a heterogeneous single-site catalyst based on a rhodium triphenylphosphine complex immobilized on SBA-15 [26]. This catalyst was active in the hydrogenation of various olefins under mild conditions (40 °C, 1 bar H_2_, 1 mmol substrate, toluene, 1.6 mol% catalyst, 24 h) and could be recycled over six cycles without any loss of activity. Interestingly, the material’s catalytic activity increased after contact with hydrogen. While the first run required 20 h for the complete conversion of the model substrate 2-cyclohexen-1-one to cyclohexanone, only 2 h were needed for quantitative conversion in the following five reaction cycles [26].

A palladium-containing catalyst was also obtained via a similar synthetic route [27], which exhibited even higher activity toward olefins (RT, 1 bar H_2_, 1 mmol substrate, toluene, 0.2 mol% catalyst, 8–12 h) and could be recycled over ten cycles without any loss of catalytic activity. This catalyst also exhibited increased catalytic activity after the first run. Preliminary photoelectron spectroscopy studies showed that, under the reductive conditions, some palladium(0) species had formed, which are commonly described in the literature as active components in catalytic hydrogenation and therefore explain the observed increase in activity upon reuse of the material [28,29,30,31,32].

Compared to bulk metals, metal nanoparticles have a higher surface-to-volume ratio and therefore usually exhibit greater reactivity [33,34]. However, it is important to prevent particles from growing via Ostwald ripening [35] or agglomerating into larger units during the synthesis or use of these materials, as this leads to a reduction in catalytic activity [36]. We attribute the high long-term stability of the materials described above to the suppression of these processes by the immobilized ligands [37].

Due to the complicated synthesis of the immobilized rhodium and palladium complexes described above, we sought easily accessible phosphine ligands containing trialkoxysilyl groups. In this work, we describe the synthesis of such a ligand, its reaction with palladium and rhodium precursors, and the immobilization of these complexes on SBA-15. Furthermore, we focused on the reduction of the palladium(II) and rhodium(I) centers to the corresponding nanoparticles using hydrogen. In contrast to the use of water soluble reducing agents (e.g., NaBH_4_, hydrazine, ascorbic acid, etc.), reduction with hydrogen can be carried out in a nonpolar medium, thereby suppressing the migration of metal species. To characterize the palladium centers on the support materials, we conducted an extensive XPS study.

## 2. Results and Discussion

### 2.1. Catalyst Synthesis

#### 2.1.1. Synthesis of Phosphine Ligand **1**

**Scheme 1:**
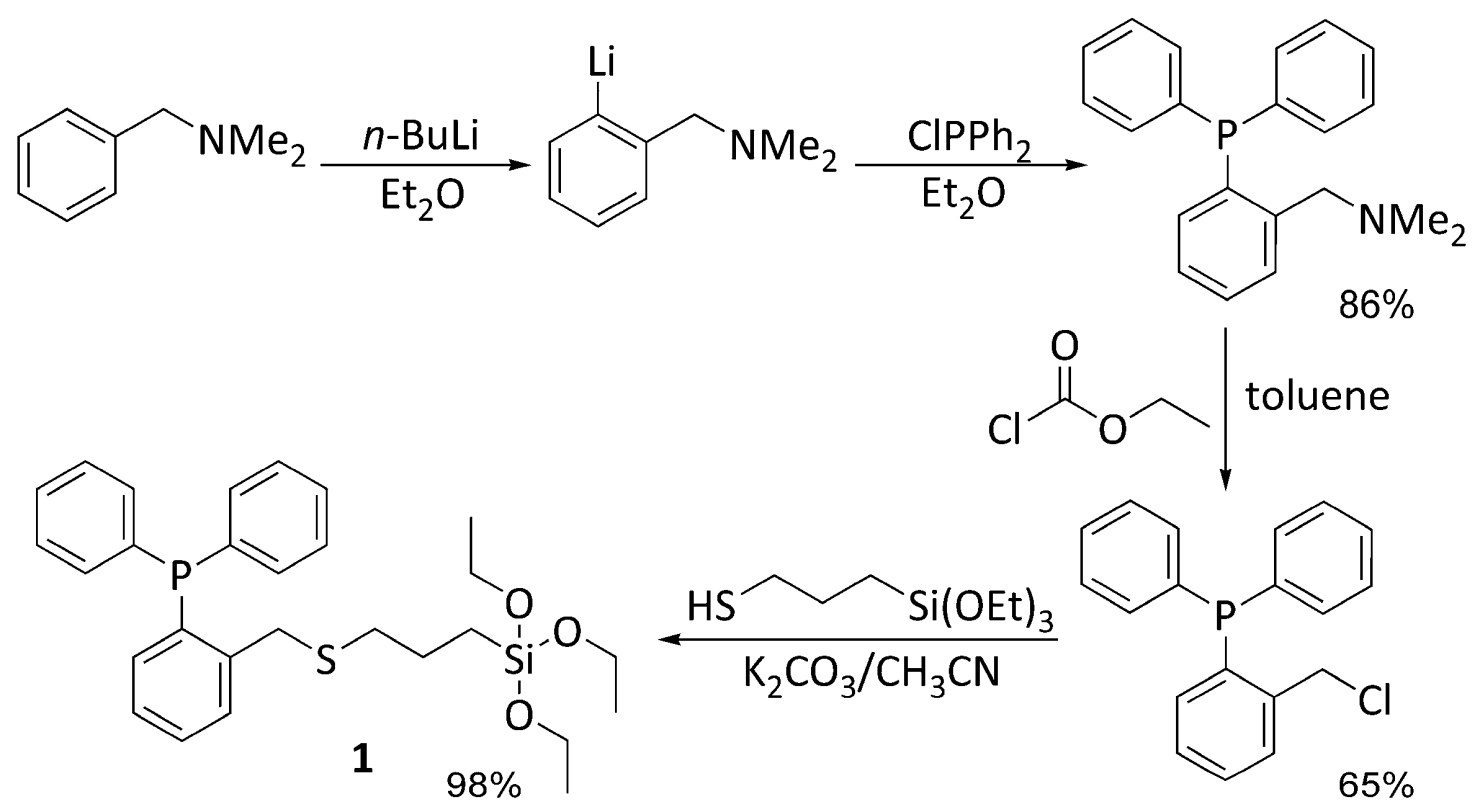
Synthesis of the phosphine ligand **1**.

The phosphine ligands used in this work were synthesized in a three-step reaction sequence starting from *N*,*N*-dimethylbenzylamine. Based on an optimized procedure by Rauchfuss et al. [38], *ortho*-lithiation with *n*-butyllithium and subsequent reaction with chlorodiphenylphosphine yielded (2-diphenylphosphino)-*N*,*N*-dimethylbenzenemethanamine (Scheme 1) in 85% yield. The ^31^P{^1^H} NMR spectrum of this intermediate shows one resonance at −15.4 ppm. After workup and purification, the reaction with chloroformic acid ethyl ester provided (2-chloromethylphenyl)diphenylphosphine in 65% yield (^31^P{^1^H} NMR: −17.0 ppm) [39,40]. This reactive intermediate was finally reacted with (3-mercaptopropyl)triethoxysilane in acetonitrile for 18 h at 82 °C in the presence of potassium carbonate as the base. The phosphine ligand **1** (^31^P{^1^H} NMR: −17.2 ppm) and its precursors were characterized comprehensively by spectroscopy and elemental analysis (see the Experimental Section and the Supporting Information).

#### 2.1.2. Palladium and Rhodium Phosphine Complexes

**Scheme 2:**
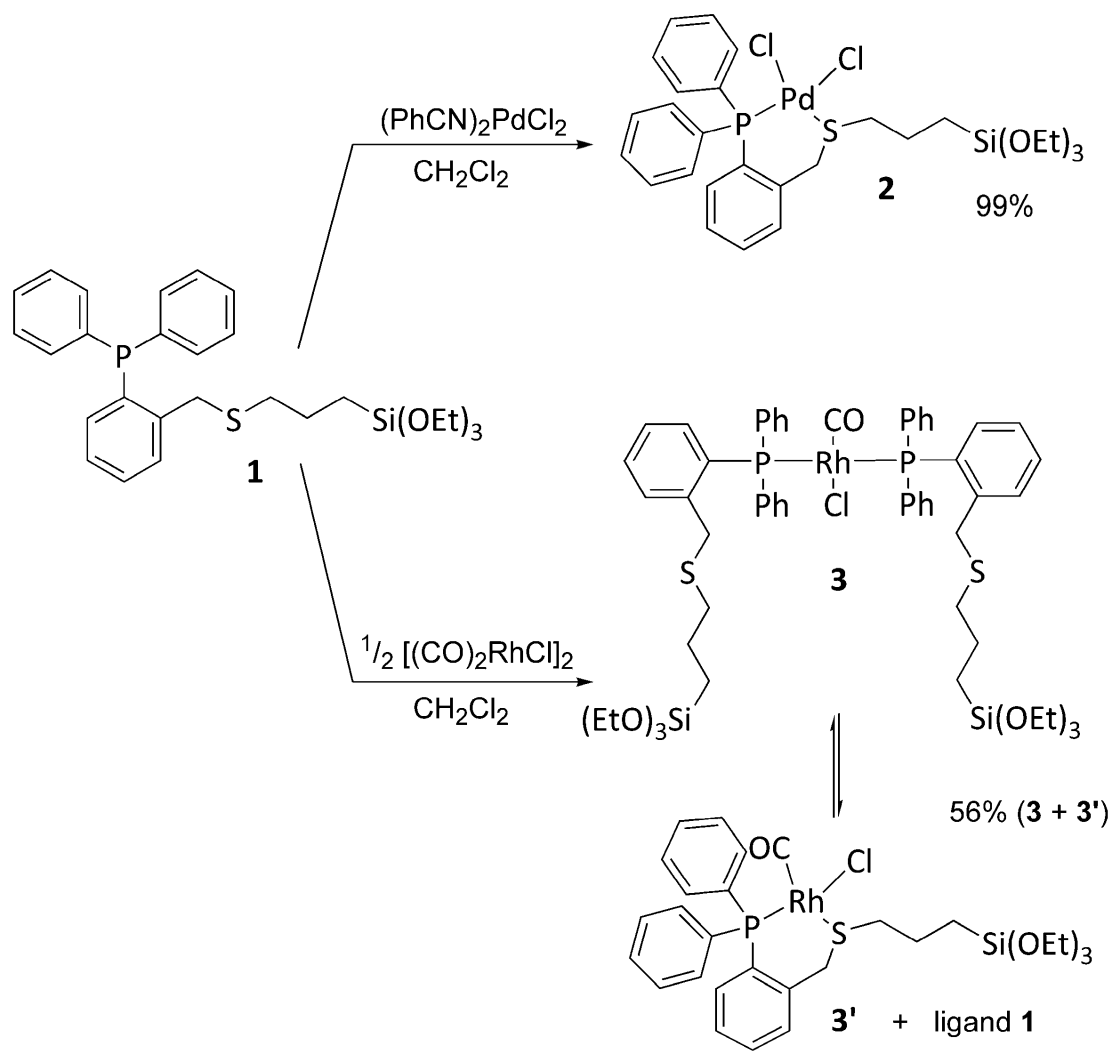
Synthesis of the palladium complex **2** and a mixture of the rhodium complexes **3** and **3′**.

The synthesis of palladium and rhodium phosphine complexes **2** and **3** was carried out according to a procedure described by us (Scheme 2) [26]. For this purpose, ligand **1** was first reacted in dichloromethane with (C_6_H_5_CN)_2_PdCl_2_ or [(CO)_2_RhCl]_2_ at a metal/ligand ratio of 1:2, under the assumption of two ligands coordinating in *trans*-orientation.

However, the ^1^H NMR spectrum and elemental analysis of the palladium complex **2** prove that, despite the excess of ligands, a complex with only one coordinating phosphine ligand is formed. The ligand coordinates bidentate to palladium via both the phosphorus and the sulfur donor. In further reactions, the molar ratios were therefore adjusted accordingly. The bidentate coordination of the ligand creates a six-membered, folded ring structure that, relative to the time scale of ^1^H NMR spectroscopy, rearranges so slowly that the diastereotropy of the two phenyl groups and all protons of the methylene units in the ligand skeleton can be observed (see the Supporting Information). All signals in the ^1^H and ^13^C NMR spectra could be assigned using 2D NMR spectroscopic methods. The ^31^P NMR spectrum confirms the analytically pure synthesis of complex **2** with only one signal at a chemical shift of 25.7 ppm.

Salmeida et al. reported on the synthesis of a structurally related palladium complex. They observed that when two equivalents of the ligand were used, a bisphosphine complex (monodentate phosphine coordination) was formed, whereas when only one equivalent was used, bidentate coordination occurred via the phosphorus and the sulfur atoms [41].

In the case of the reaction of ligand **1** with [(CO)_2_RhCl]_2_ at a metal/ligand ratio of 1:2, the IR spectrum shows the expected strong band of a C=O stretching vibration at 1971 cm^−1^, the position of which is typical for *trans*-configured rhodium bisphosphine complexes [42,43,44,45]. However, a shoulder at a wavenumber of 2008 cm^−1^ indicates that a small amount of a second species with a carbonyl ligand might be present. The ^1^H NMR spectrum of the synthesis carried out with the rhodium precursor [(CO)_2_RhCl]_2_ shows the signals of diastereotopic protons already observed in palladium complex **2**, indicating a bidentate *P,S*-coordination of one ligand, which we assign to complex **3′**. In addition, the spectrum is characterized by a large number of very broad signals, indicating pronounced dynamics. The ^31^P NMR spectrum shows the signal of the free ligand **1** and a doublet at a chemical shift of 20.3 ppm (^1^*J*_RhP_ = 125 Hz) as well as some smaller signals that cannot be definitively assigned. We interpret these findings as a dissociation/association equilibrium between a rhodium bisphosphine complex **3** on the one hand and a rhodium complex **3′** with a *P,S*-donating ligand and free ligand **1** on the other. If we calculate the elemental analysis under these assumptions, we obtain a ratio of bis- to monophosphine complex of 4:1, which suggests that a small proportion of ligand is lost during purification of the product. Suomalainen et al. report a wavenumber of 2010 cm^−1^ for the C=O stretching vibration in a *P,S*-coordinated rhodium carbonyl chloride complex [46], which confirms the shoulder in the IR spectrum mentioned above.

#### 2.1.3. Synthesis of the Catalyst Materials

**Scheme 3:**
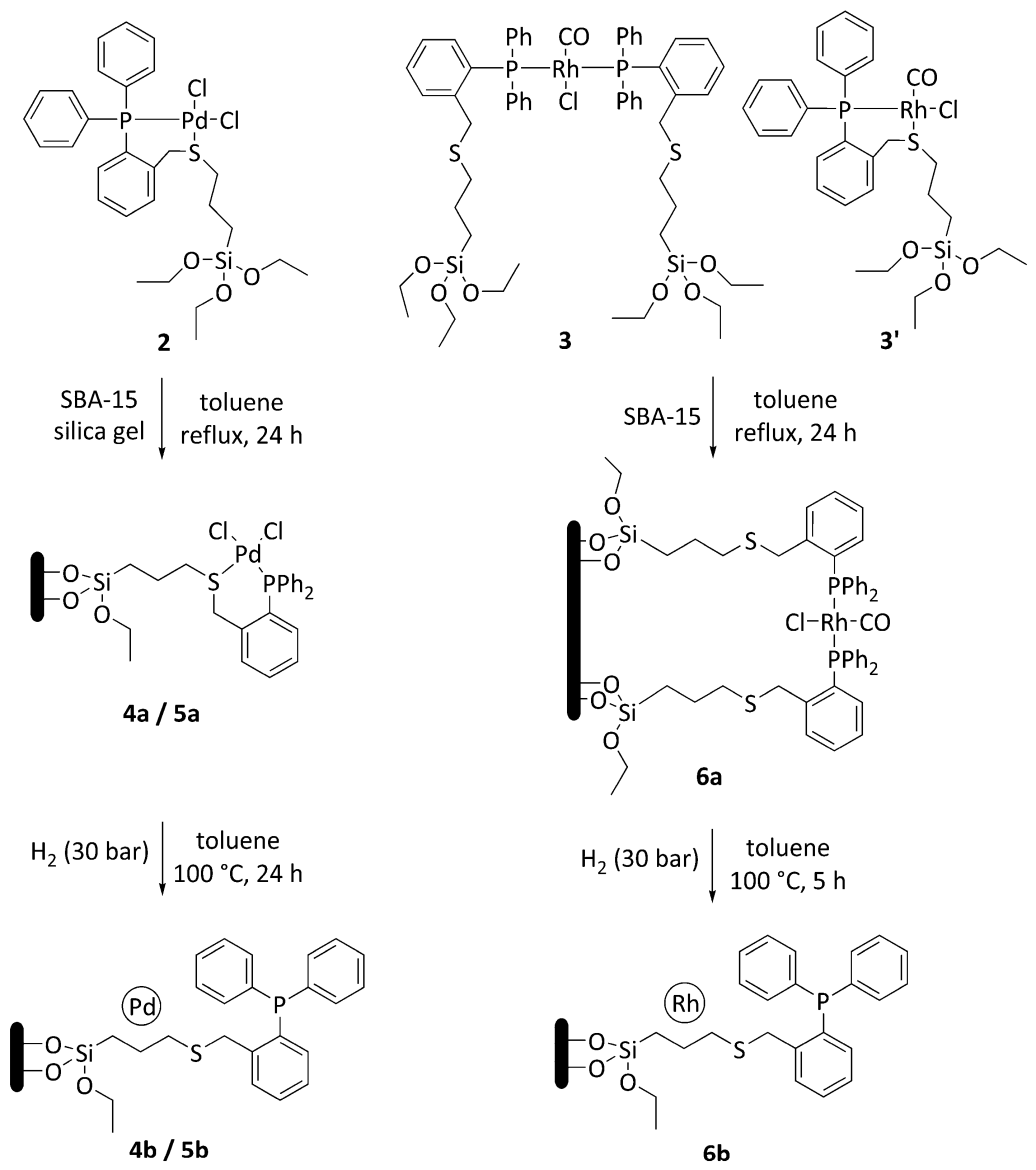
Synthesis of the catalyst materials catalysts **4b**, **5b**, and **6b**.

The immobilization of the palladium complex **2** onto the surfaces of SBA-15 [17,18] and commercially available silica gel (see the Exp. Section for details) resulted in materials **4a** and **5a**, respectively. Material **6a** was obtained from the reaction of the mixture of rhodium complexes **3** and **3′** with SBA-15. To achieve this, 0.35 mmol of the complexes were reacted with 1.0 g of the appropriate silica gel in toluene at 110 °C for 24 h. The intermediates **4a**, **5a**, and **6a** obtained in this way were characterized in detail and then reduced with hydrogen to provide the final catalysts **4b**, **5b**, and **6b** (Scheme 3).

All samples were characterized using elemental analysis, infrared spectroscopy, CP MAS NMR spectroscopy, nitrogen physisorption measurements, and scanning electron microscopy.

Significant amounts of carbon and sulfur in **4a,b**, **5a,b**, and **6a,b** indicate the successful immobilization of complexes **2** and **3** on the support materials (Table 1). In addition, metal contents between 2.10–2.50 wt% were detected. After reduction of the metal centers, the carbon content decreased slightly, whereas the weight percentage of the metal increased. The loss of carbon can be explained by minor losses of organic components, e.g., silicon-bound ethoxy groups, during reduction.

**Figure 1:**
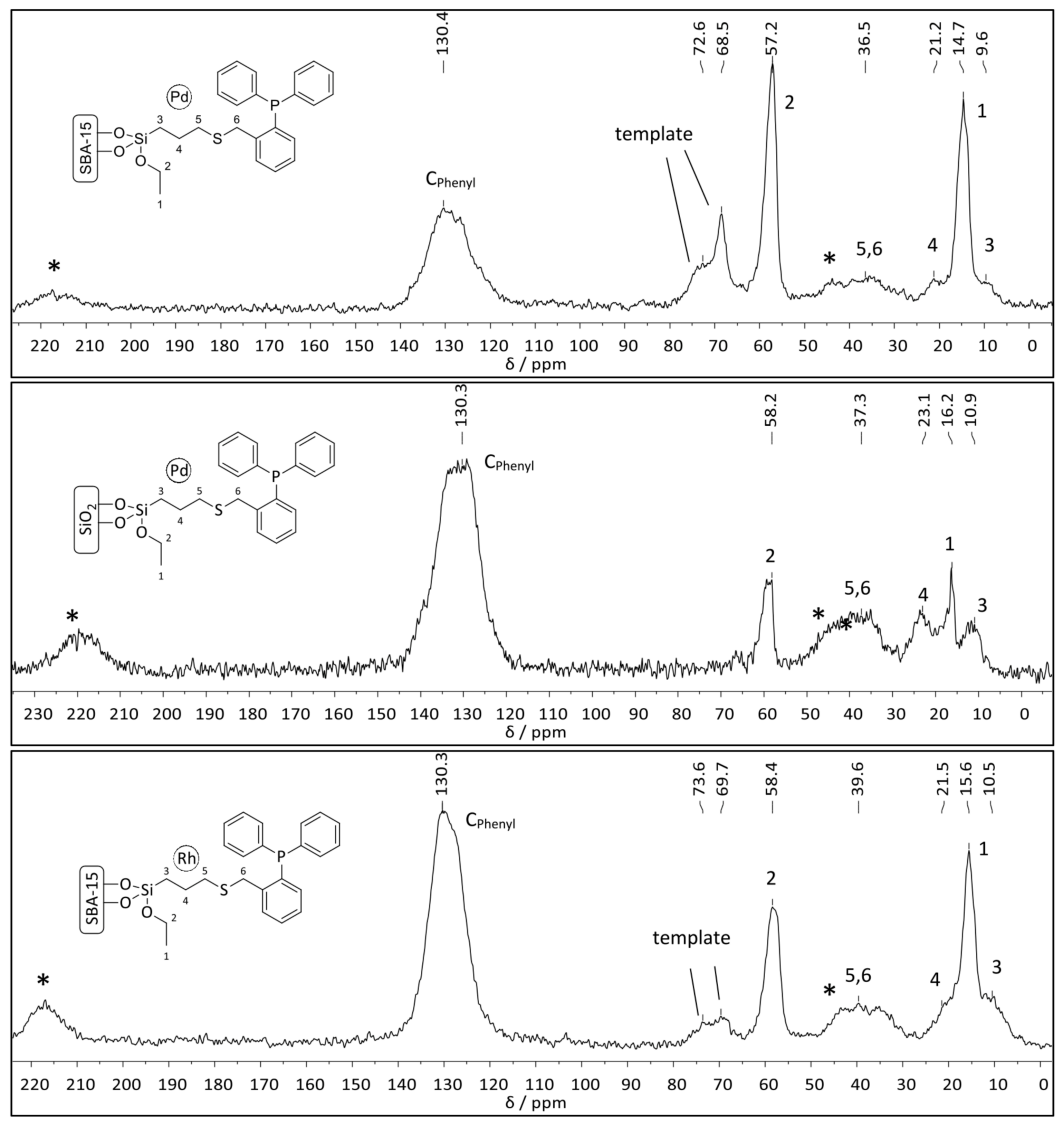
^13^C CP-MAS NMR spectra of materials **4b**, **5b**, and **6b**. The * denotes rotational sidebands.

^13^C and ^31^P CP-MAS NMR spectroscopy also confirms the successful immobilization of the complexes. Since the spectra of intermediates **4a**, **5a**, and **6a** are very similar to those of the reduced samples **4b**, **5b**, and **6b** (see the Supporting Information), Figure 1 shows exemplary the ^13^C CP-MAS NMR spectra of materials **4b**, **5b**, and **6b**. All signals could be assigned.

While the chemical shifts observed for **4a,b** and **5a,b** in the ^31^P CP MAS NMR (approx. 26 ppm) correspond well to the value recorded for the precursor **2**, the chemical shift observed for materials **6a,b** (34.7 and 35.4 ppm) are somewhat different to the resonance assigned to compound **3** in the high-resolution ^31^P NMR spectrum (20.3 ppm). It might be that by binding to the surface, one of the two phosphine ligands bound to compound **3** gets lost. The typical T resonances of the species R-Si(OSi)_3_ and R-Si(OSi)_2_(OH) at approx. −45 to −60 ppm in the ^29^Si CP-MAS NMR spectra of **4a,b**, **5a,b**, and **6a,b** prove the covalent bonding of the precursor complexes to the silica gel matrix of the carrier materials (see the Supporting Information). Here as well, no change in the spectra is observed after reduction of the materials.

The pore characteristics of the materials were determined using nitrogen physisorption measurements. Table 2 summarizes the measured data.

Several trends can be deduced from these data: the specific surface area of the materials increases with reduction. Treatment with hydrogen under pressure and at elevated temperatures may remove remaining organic compounds (e.g., the template) from the surface. The specific pore volume also increases slightly in parallel. The average pore diameter decreases slightly, which can be explained by the opening of previously closed small pores during the reduction process. However, the overall morphology of the materials remains unchanged as shown in SEM images (see the Supporting Information).

To obtain more detailed analytical data on materials **4a,b**, **5a,b**, and **6a,b**, EFTEM, element mapping TEM imaging and element mapping analyses by EDX measurements were carried out. A detailed XPS analysis was additionally performed on **4a,b**. Since the materials were examined both before and after reduction, conclusions can be drawn about changes occurring during the reduction process.

**Figure 2:**
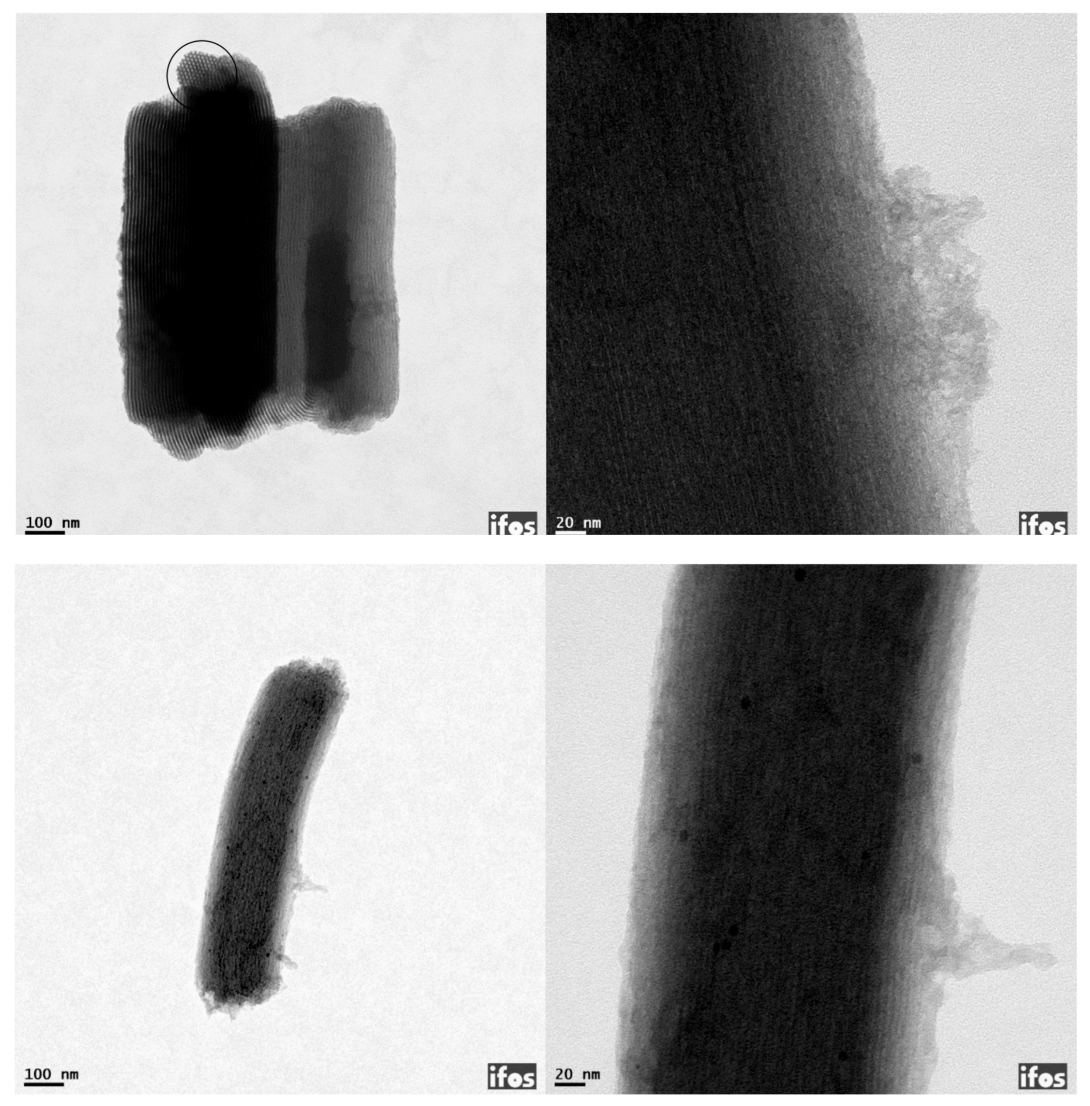
EFTEM images of **4a** (**top**) and **4b** (**bottom**); right images: five-fold enlargement of the left images. The circle in the top-left image points to a section where the hexagonal structure of the SBA-15 is clearly visible.

In the EFTEM images of materials based on the mesoporous silica gel SBA-15, the hexagonal pore structure characteristic of this carrier becomes apparent [17,18]. The pore diameters of approx. 5 nm derived from these images correspond well with the data from the nitrogen physisorption measurements (see Table 2). Figure 2 shows EFTEM images of **4a** and **4b** as an example. While no palladium particles are visible in **4a**, particles with estimated diameters of 2–5 nm can be seen in the image of **4b**. The immobilized ligand structures thus contribute to stabilizing the metal particles.

**Figure 3:**
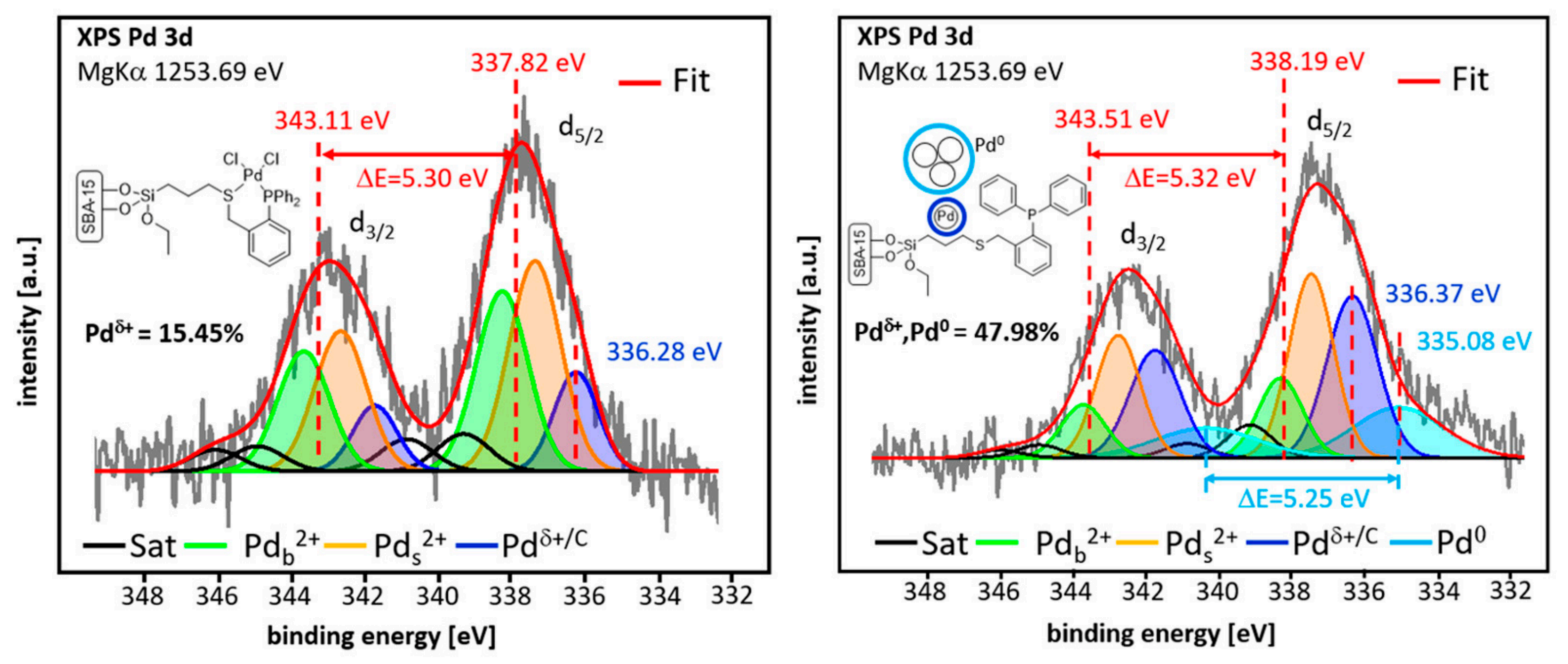
Charge corrected Pd 3d XPS detail spectra (grey line), fit analysis (red line), spin–orbit coupling ΔE and percentage Pdδ+/Pd0 for materials **4a** (**left**) and **4b** (**right**). The guidelines (red dashed) to evaluate the spin–orbit coupling ΔE are centered between the two Pd (II) peaks (green, orange). For further details please refer to the main text.

Element imaging by EDX that is coupled with element mapping TEM imaging, confirms the presence and the distribution of all elements expected to be present in the materials (see the Supporting Information). To analyze the nature and oxidation state of palladium in the native material **4a** and the reduced material **4b**, we used X-ray photoelectron spectroscopy (XPS). In the survey spectra of **4a,b** the typical signature of the SBA-15 framework elements and of the palladium sites are clearly identified (see the Supporting Information). For the identification of the oxidation state of the palladium atoms, we measured the Pd 3d core level peak. Figure 3 shows the changes in the 3d detail spectra before (**4a**, left) and after the reduction process (**4b**, right). In general, the typical 3d peak is split into the Pd d_3/2_ and the Pd d_5/2_ peak due to the spin–orbit coupling.

Because of the overall shape and partial asymmetry of the peaks, the underlying peak structure was analyzed using an appropriate fitting routine which we have recently applied also successfully to other Pd complexes [47,48]. The resulting fit analysis in Figure 3 shows up to four different palladium species.

According to our previous XPS analyses on matrix-supported Pd complexes and in accordance with the literature [47,48], we assign the peaks as follows: For both materials, the orange-colored fraction is associated with palladium(II) species being located near or the surface of the SBA-15 matrix, while the green-colored fraction is associated with palladium(II) species being located more in the pore channels [47]. The observed difference in binding energies between these two palladium(II) species of 0.86 eV for material **4a** and 1.05 eV for material **4b** can be attributed to a difference in charge compensation of these ions during the photoemission process. Palladium(II) ions near the surface (orange) can more efficiently compensate for the positive charge generated by photoelectron emission than palladium(II) ions embedded in the pore channels (green), resulting in lower measured binding energies. Additionally to the core level peaks of each palladium(II) species, accompanying satellites must be taken into account (black) [49]. The measured differences in binding energies is consistent with the results of 1.3 eV found by Roger et al., who also differentiated in their study between PdO being located on the surface and PdO being present in the cavities of the material for PdO on Al_2_O_3_ [50]. Using the center between the palladium(II) related 3d peaks (red dashed line in Figure 3), the 3d binding energies of 337.82 eV and 343.11 eV for **4a** and 338.18 eV and 343.51 eV for **4b** as well as the spin–orbit coupling of ΔE = 5.30 eV and ΔE = 5.32 eV are all in good agreement with 3d binding energies also obtained for palladium(II) species in similar environment [27,47,48,51]. The blue fraction in Figure 3, left for **4a** describes the presence of zero-valent polarized Pd^δ+^ with a fraction of 15.5% of the total 3d peak area, whose polarization is caused by coordination with phosphorus or sulfur. Energetically similar values for Pd^δ+^ palladium were observed by Jing et al. in Pd_4_S nanocrystal catalysts on PPh_3_-modified activated carbon, which supports the correctness of this assignment [52]. An interpretation of the fraction of Pd^δ+^ in **4a** is given below (Section 2.2.1).

The XPS measurement of **4b** (after the reduction step, Figure 3) shows a significantly altered picture. It confirms two different zero-valent Pd species causing a slight asymmetry in the low-energy flank of the peaks in comparison to the non-reduced material **4a.** As a result, in addition to the also in **4b** included Pd^δ+^ at 336.37 eV, the formation of additional unpolarized zero-valent Pd^0^ species at 335.08 eV and a spin–orbit coupling of ΔE = 5.25 eV can be identified (turquoise fraction). This binding energy and ΔE values are in the range of palladium nanoclusters [53]. These peaks are somewhat broader than the other 3d peaks, which can be explained by the different binding energies of clusters of different sizes. Smaller clusters exhibit higher binding energies than larger agglomerates or the pure metallic phase [53]. If all zero-valent species Pd^δ+^/Pd^0^ are summed together, the total proportion of reduced palladium is calculated to be 48.0%.

The palladium species on the substrate were therefore not completely reduced under the given reduction conditions, since in addition to Pd^0^ clusters and polarized Pd^δ+^/species, the XPS spectrum of **4b**, shows also palladium(II) centers, but in a significantly smaller proportion (−32.5%). Nevertheless, a change in catalytic activity compared to the non-reduced sample is to be expected, since metal clusters have been discussed as an active phase in hydrogenation reactions [54,55,56]. However, even the presence of species of different oxidation states in one catalyst has found to be advantageous. Yang et al. and Barbaro et al. described hydrogenation catalysts in which the catalytic activity of rhodium(I) complexes on reduced Pd/SiO_2_ was higher than that of reduced Pd/SiO_2_ alone or the immobilized rhodium complex grafted separately onto silica gel [57,58].

For TEM imaging and EDX measurements we further focused our investigations on **5a,b**. The EFTEM images (see the Supporting Information) show the irregular and diffuse structure of the support material, which was also observed in this form by Kalumpha et al. The pores are not uniform in size, which explains the inhomogeneous pore size distribution already evident from the BJH plot of the nitrogen physisorption data. In the reduced material **5b**, palladium(0) particles are not clearly visible in the TEM image because the silica particles have a high intrinsic contrast. The EDX data confirms the expected composition of **5a,b** (see the Supporting Information). The expected elements are distributed homogeneously throughout the entire sample area.

Finally, **6a,b** were examined using EFTEM, element mapping TEM and EDX (see the Supporting Information). Here, too, the pore channels characteristic of SBA-15 are evident. Rhodium, carbon, oxygen, silicon, phosphorus, and sulfur were detected in the EDX spectra of the material. The element distribution on the substrate is homogeneous, as in the previously discussed materials **4a,b** and **5a,b** (see the Supporting Information). In the TEM images of the reduced catalyst **6b**, extremely small darker areas (approx. 1 nm) can be seen, particularly in the edge areas, which cannot be unambiguously attributed to rhodium nanoparticles. This may be due to the reduced reduction time of 5 h compared to the palladium-containing materials (24 h).

### 2.2. Catalytic Olefin Hydrogenation

#### 2.2.1. Evaluation of Reaction Conditions

In preliminary tests, it was found that **4a,b**, **5a,b**, and **6a,b** are capable of quantitatively hydrogenating the substrate 2-cyclohexen-1-one to cyclohexanone in toluene as the solvent within 2 h at a reaction temperature of 100 °C and a hydrogen pressure of 15 bar. To optimize the reaction conditions, first the temperature dependence of the hydrogenation of 2-cyclohexen-1-one with **4b** was investigated. All other parameters were kept constant. At 80 °C and 60 °C, the yields of cyclohexanone after 2 h were quantitative, while at 40 °C the yield was slightly reduced to 89%. To ensure comprehensive comparability of all catalysts, it was decided to carry out the hydrogenation at room temperature under 1 bar of hydrogen pressure and to monitor the reaction progress over a period of 5 h. Under these conditions, catalyst **4b** is no longer active in toluene. To overcome this problem, various solvents were tested with **4b** to determine their suitability for this reaction. In isopropanol and cyclohexane, 2-cyclohexen-1-one was quantitatively hydrogenated to cyclohexanone at room temperature and 1 bar of hydrogen. Even without solvent, the yield was quantitative. 1,4-Dioxane yielded only 48% cyclohexanone. A possible activity of isopropanol and 1,4-dioxane as hydrogen donors (transfer hydrogenation) was ruled out by the absence of reaction in these solvents in the absence of hydrogen. When ethanol was used, the formation of 1,1-diethoxycyclohexane was observed, which is why this solvent was not further investigated. The occupancy of the active centers could be responsible for the inactivity of toluene when hydrogenating under very mild conditions. A dominant influence of hydrogen solubility can be ruled out. The literature cites solubilities of approx. 2–5 mol/g (298.15 K, 1013 mbar) of hydrogen in the solvents used here [59,60]. Cyclohexane was used as the solvent for the following investigations because it proved to be particularly suitable for reaction analysis.

**Figure 4:**
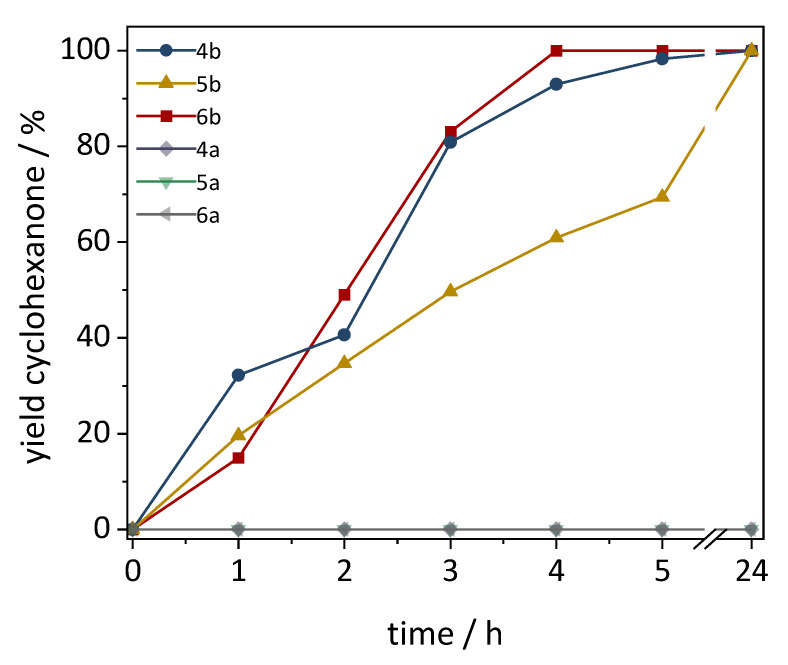
Time-dependent yields of the hydrogenation of 2-cyclohexen-1-one catalyzed with materials **4a,b**, **5a,b**, and **6a,b** (reaction conditions: RT, 1 bar H_2_, 1 mmol substrate, 2 mL cyclohexane, 0.2 mol% Pd/Rh), yields determined by GC-FID.

After determining the reaction conditions, the catalytic activity of **4a,b**, **5a,b**, and **6a,b** in the hydrogenation of 2-cyclohexen-1-one was compared. Figure 4 summarizes the results.

The materials **4a**–**6a**, which were not pre-reduced are not catalytically active under mild hydrogenation conditions, and their activation by hydrogen is not possible within the given reaction time. The pre-reduced materials **4b**–**6b**, on the other hand, are highly active. This raises questions regarding the small proportion (15.5%) of polarized zero-valence Pd^δ+^ species found in the XPS study of **4a**, from which at least a low level of hydrogenation activity would have been expected. Either these species are formed under the conditions of the XPS measurements, or they are already formed during the synthesis of **4a** due to the high reaction temperature but are then inactive. If this is the case, then this proportion of Pd^δ+^ species should also play no role in the activity of the pre-reduced material **4b**. In contrast to **4a**, the XPS spectrum of **4b** contains signals that can be attributed to palladium nanoclusters. One hypothesis is that these are the carriers of the hydrogenation activity, which corresponds to findings in the literature [54].

The same should apply to the high activity of **5b** and **6b**. However, no metal nanoparticles in a detectable size range were visible in the EFTEM images of these catalysts. We attribute this to the poor contrast of very small nanoparticles under the given measurement conditions.

The immobilized organic ligands result in a somewhat less polar surface of the materials. Lazar et al. investigated the hydrogenation activity of rhodium and ruthenium complexes, which they immobilized on different mesoporous support materials. They found that periodically mesoporous organosilicates (PMOs) led to higher catalytic activity than pure SBA-15 silica gel and attributed this result to the altered pore structure of the PMOs on the one hand and to faster adsorption–desorption processes on the less polar surfaces on the other [61]. Studies dedicated to this effect in detail are currently being conducted in our working group.

When comparing the carrier materials, catalyst **4b** (on SBA-15) showed higher catalytic activity than **5b** (unstructured silica gel), which underscores the particular suitability of SBA-15 as a support material. The nitrogen physisorption measurements (Table 2) show a significantly smaller specific surface area and a lower specific pore volume for **5b** with approximately identical average pore diameters. Together with the measurement of the catalytic activities, this can be interpreted as an unfavorable ratio of smaller and larger pores in the unstructured silica gel of **5b**.

In particular the activities of the two palladium catalysts **4b** and **5b** in the hydrogenation of 2-cyclohexen-1-one are high even when compared to the best data from the literature on other supported palladium catalysts. Sharma et al. reported a hydrogenation catalyst consisting of palladium deposited on C3N4 showing complete conversion of the substrate at room temperature and 1 bar of hydrogen after 12 h, requiring irradiation with a 15 W white light LED [62]. Bäckvall et al. published a foam-type material having large pores, which they used to deposit palladium nanoparticles and applied it for the hydrogenation of a series of olefins in a flow system. It rapidly (20 min) and quantitatively hydrogenates 2-cyclohexen-1-one. However, the residual time is not given, and the loading of palladium is much higher than in our case [63].

#### 2.2.2. Substrate Scope

After realizing that the pre-reduced catalysts were the much more catalytically active ones, these were used in the reduction of further organic substrates. Table 3 contains all evaluated substrates with yields after 5 h and 24 h, respectively.

Materials **4b**–**6b** are capable of catalyzing the hydrogenation of a broad scope of olefins under mild conditions. Compared to 2-cyclohexen-1-one, 2-cyclopenten-1-one reacts more slowly with yields of cyclopentanone of 32%, 21%, and >99% after 24 h (Table 3, entry 2). **6b** is the most active catalyst for this substrate. This also applies to the hydrogenation of mesityl oxide (entry 3). Cinnamaldehyde is selectively reduced at the C=C double bond to hydrocinnamaldehyde, but only in yields of up to 20% after 24 h (entry 4). With styrene and α-methylstyrene, yields of ethylbenzene and cumene of >99% are achieved after 24 h (entries 5 + 6), with **4b**. α-Methylstyrene is quantitatively converted to cumene after only 2 h. 4-Methylstyrene was quantitatively hydrogenated to 1-ethyl-4-methylbenzene after only 1 h, whereas **5b** required 4 h for quantitative conversion. In this case, **6b** showed significantly less activity with a yield of only 38% after 24 h (entry 7). *Trans*-stilbene was quantitatively converted to 1,2-diphenylethane within 24 h by both the palladium-containing materials and the rhodium-containing material (entry 8). Corresponding reaction rates were observed in the hydrogenation of 3,4-dihydro-2H-pyran to tetrahydropyran, with the exception of the reaction with **5b** (entry 9). Finally, *trans*-anethol was reduced to 4-propylanisol. Here, a yield of 6% 4-propylanisol after 5 h indicated another drop in the catalytic activity of **6b**. **4b** catalyzed the reduction quantitatively after only 2 h, whereas after 5 h with **5b** a conversion of 81% was achieved (entry 10).

#### 2.2.3. Catalyst Recycling

**Figure 5:**
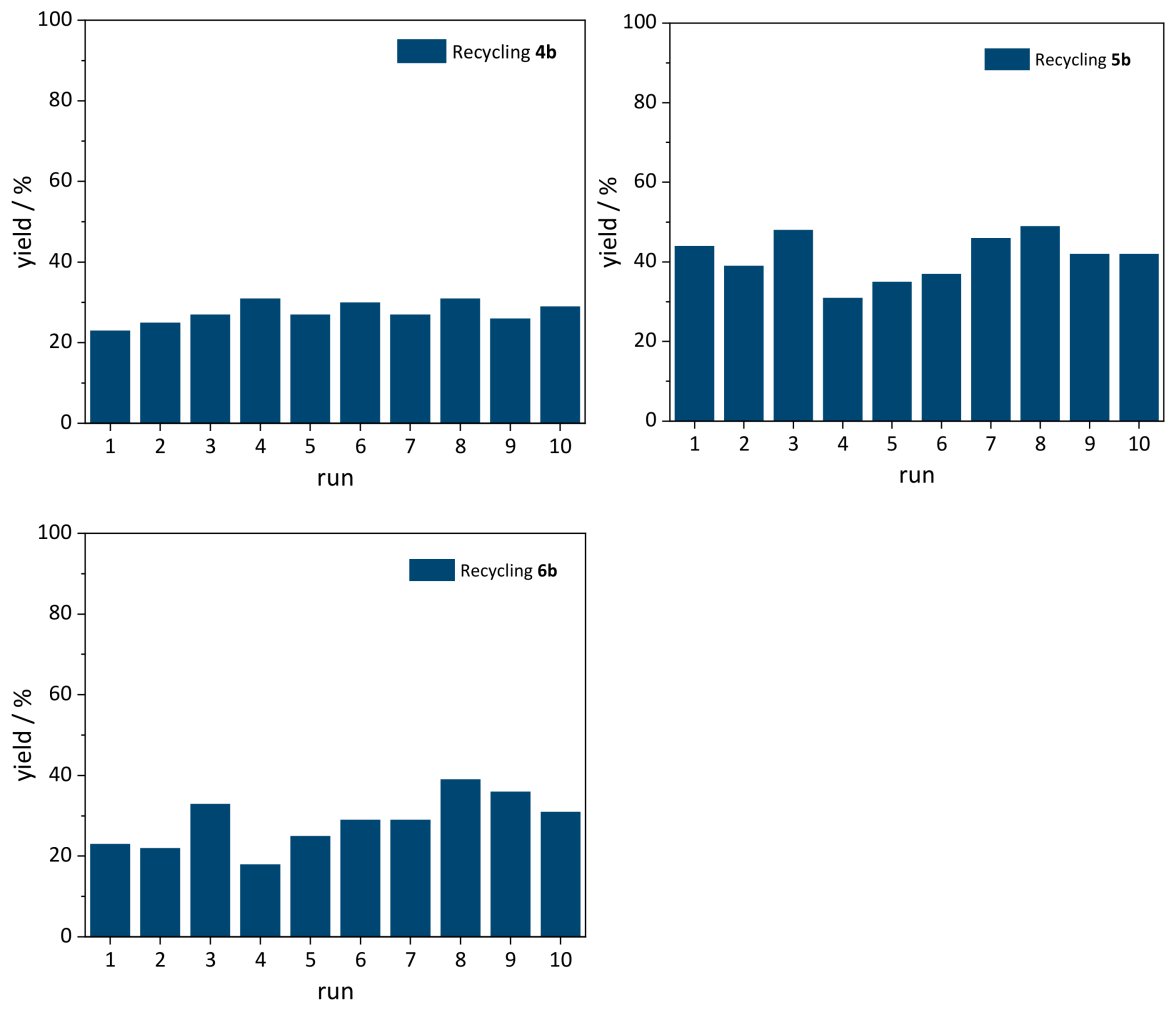
Recycling of **4b** (**top left**), **5b** (**top right**) and **6b** (**bottom**) over ten cycles; reaction parameters: RT, 1 bar H_2_, 1 mmol of 2-cyclohexen-1-one, cyclohexane (2 mL), 1.2 mol% of palladium, 1 h.

Following the application of the catalysts in hydrogenation reactions, their recycling was investigated. This study was conducted exemplarily with material **4b** as the catalyst and with 2-cyclohexen-1-one as the substrate. The detailed setup of this process is described in the Supporting Information. Using 1.2 mol% **4b**, a yield of approximately 25% cyclohexanone was achieved after 1 h. This level of yield was chosen specifically to better identify possible changes during the recycling experiments. Catalyst **4b** showed consistent catalytic activity over ten hydrogenation cycles (Figure 5, top left), while high selectivity was still maintained. A slight increase in activity during the first four cycles may be due to an ongoing reduction of palladium(II) species (see below) and formation of palladium nanoparticles. Further slight fluctuations in the yields of a can be explained on the one hand by measurement inaccuracies of the gas chromatography, and on the other hand by minor differences in the experiment preparation. The loss of catalyst was <0.5 mg per batch.

**Figure 6:**
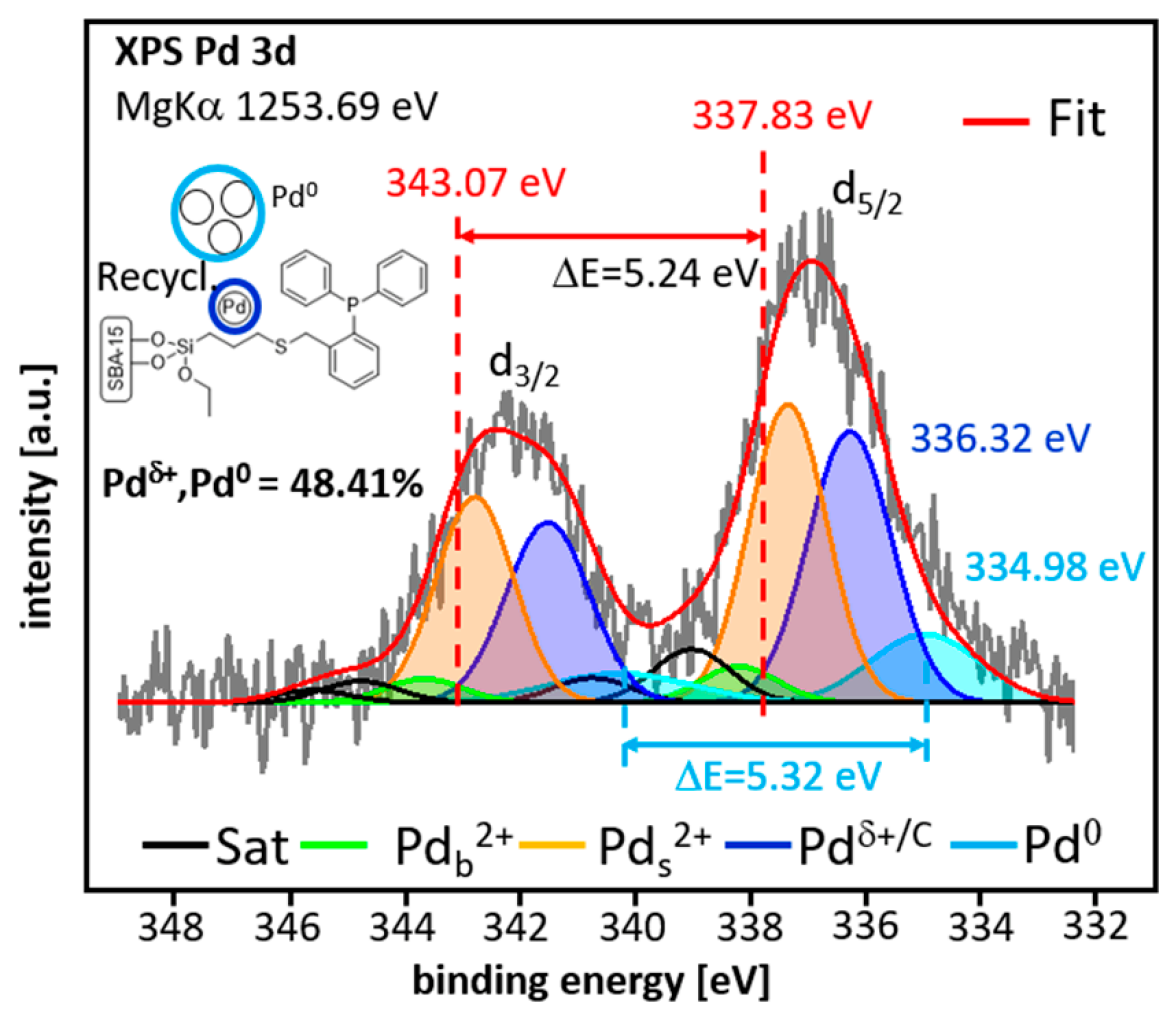
Palladium 3d XPS spectrum (gray line) and fitting curves according to Figure 3 for material **4b** after tenfold recycling.

The tenfold recycled catalyst **4b** was characterized again using ^13^C CP-MAS NMR spectroscopy, SEM, XPS, and EFTEM (see the Supporting Information). In the ^13^C CP-MAS NMR spectrum new resonances were identified at chemical shifts of 24.2 ppm, 16.2 ppm, and −2.2 ppm, which could originate from residual carbon impurities from previous hydrogenations or indicate a change in the ligand structure. The morphology of the material remains unchanged after ten catalysis cycles. No significant changes are noticeable in the EFTEM images either. The dark contrasts (see Figure 2), which are agglomerates of palladium atoms, remain unchanged in frequency and diameter. When looking at the Pd 3d_5/2_ peak in the XPS spectrum in Figure 6, again the asymmetry of the 3d peaks changes slightly.

This is caused by two facts: First the relation between the palladium(II) surface fraction and the bulk fraction changes in favor of the surface fraction. Second, the Pd^δ+^/PdO relation changes too. The binding energy of 334.98 eV and the spin–orbit coupling ΔE for the PdO nanocluster fraction doesn’t really change compared to freshly prepared **4b**. The total proportion of 48.4% for the zero-valent Pd^δ+^/Pd^0^, remains basically unchanged even after ten hydrogenation cycles (previously 48.0%), which proves the stability of this species under hydrogenation conditions. The evaluation was carried out according to Akiri et al. [47]. The catalysts **5b** and **6b** could also be successfully recycled over ten hydrogenation cycles (see the Supporting Information).

## 3. Materials and Methods

*General*: The chemicals used for syntheses and catalytic reactions were purchased commercially and used without further purification. The hydrogen gas used was obtained from Air Liquide Deutschland GmbH (Düsseldorf, Germany) with a purity of ≥99.999%. Acetonitrile was heated over CaH_2_ for several hours under inert gas (N_2_) to achieve absolute purity. Diethyl ether, dichloromethane, toluene, and *n*-pentane were taken from a solvent drying unit (MBraun MB-SPS, M. Braun Inertgassysteme GmbH, Garching, Germany) and then freed of oxygen by passing through nitrogen for 15 min.

Silica gel (CAS No. 112926-00-8) was purchased from Sigma Aldrich Chemie GmbH (Taufkirchen, Germany) and used without further purification. The specific surface area, pore volume, and average pore diameter of this material are 500 m^2^/g, 0.75 cm^3^/g, and 6.0 nm, respectively. SBA-15 was synthesized according to a method described in the literature [64]. The triblock copolymer P123 used for this purpose was removed exclusively by extraction. The SBA-15 material was not calcined. Standard Schlenk techniques were used for syntheses that require protection gas and the use of degassed and dried solvents. All glassware used for this purpose was heated several times under vacuum using a hot air gun and charged with nitrogen before the synthesis was started. The syntheses of 1-(2-(diphenylphosphino)phenyl)-*N,N*-dimethylmethanamine [38,65,66,67,68] and of (2-(chloromethyl)phenyl)diphenylphosphine [39,40] were performed according to the literature.

The high-resolution NMR spectra in solution were measured on a Bruker Avance III 400 FT-NMR spectrometer (Bruker Corporation, Billerica, MA, USA) at room temperature (295 K). The remaining protons of the solvents were used to reference the ^1^H NMR spectra, and the signals of the deuterated solvents were used to reference the ^13^C{^1^H} NMR spectra. No external standard was used when measuring ^31^P NMR spectra. The solid-state NMR spectra were recorded using a Bruker Avance III 500 solid-state NMR spectrometer. The samples were rotated in ZrO_2_ rotors under magic angle spinning (MAS) conditions at 11 kHz. The ^13^C NMR and ^29^Si NMR measurements were performed with cross polarization (CP) to enhance the signal intensity. Adamantane was used to reference the chemical shifts of the carbon spectra, and the shift of the silicon nucleus was calibrated with tetrakis(trimethylsilyl)silane [69]. The NMR spectra were analyzed using the MestReNova software, version 12.0.1-20560 (Mestrelab Research, S.L.U., Santiago de Compostela, Spain).

The percentages of carbon, hydrogen, nitrogen, and sulfur were determined using the vario MICRO Cube device from Elementar Analysesysteme GmbH (Langenselbold, Germany). The metal content (palladium and ruthenium) of the materials was determined using atomic absorption spectroscopy. After microwave digestion CEM Discover SP-D (CEM, Kamp-Lintfort, Germany) in aqua regia, the measurement was carried out in an air/acetylene mixture using the contrAA 700 device (Analytik Jena, Jena, Germany).

IR spectra were recorded using a Spectrum 100 FT-ATR-IR spectrometer (Perkin Elmer). The measurement range was 650–4000 cm^−1^. After measurement, the spectra were smoothened using the Spectrum v6.3.5 software (Perkin Elmer). The graphical presentation was performed using Origin 2022b SR1 9.9.5.171 software (OriginLab).

The nitrogen physisorption measurements were performed using a BELSORP miniX (MICROTRAC MRB) device (Microtrac Retsch GmbH, Haan, Germany). The method developed by S. Brunauer, P. H. Emmett, and E. Teller (BET) was used to determine the specific surface area [70]. while the pore radius distribution and pore volume were determined using the method developed by E. P. Barrett, L. G. Joyner, and P. P. Halenda (BJH) [71]. The Origin 2022b SR1 9.9.5.171 software (OriginLab) was used for the graphical presentation.

The conversions and yields of the catalytic reactions were determined using a Clarus GC 580 gas chromatograph (PerkinElmer, Shelton, CT, USA) equipped with a flame ionization detector (FID). An FS-EnantioSELECTbeta1 (CS-Chromatography Service GmbH, Langerwehe, Germany) was used as the column. Parameters: d_i_ 0.25 mm, length 30 m, carrier gas helium 60 kPa, combustion gases (FID) synthetic air/hydrogen (10:1), V_i_ 1 μL, T_inj_ 250 °C, T_det_ 320 °C, T_start_ 80 °C, heating rate 4 K/min. The measurements were evaluated using Total Chrom software (Perkin Elmer). Yields and conversions in the recycling studies were determined using a Clarus GC 590 gas chromatograph (Perkin Elmer, Shelton, CT, USA) coupled with an SQ 8 S mass spectrometer (Perkin Elmer). An Elite-5MS (Perkin Elmer, Shelton, CT, USA) was used as the column. Parameters: d_i_ 0.25 mm, length 30 m, carrier gas helium 68 kPa, V_i_ 1 μL, T_inj_ 290 °C, T_det_ 300 °C, T_start_ 80 °C, T_end_ 120 °C, heating rate 4 K/min. The measurements were evaluated using TurboMass 6.1.2 software (Perkin Elmer, Shelton, CT, USA).

SEM images were taken using a SU8000 scanning electron microscope (Hitachi High-Tech Europe GmbH, Krefeld, Germany). Prior to measurement, the samples were coated with iridium using an EM ACE600 sputter coater (Leica Microsystems GmbH, Wetzlar, Germany) and then examined in the scanning electron microscope at a voltage of 5.0 kV. EFTEM imaging was performed on a JEOL 2010 (JEOL Ltd., Tokio, Japan) device. The electron beam was generated using a LaB_6_ cathode at an acceleration voltage of 197 kV. A Si(Li)-XEDS system (Oxford Instruments, High Wycombe, United Kingdom) was used for elemental analysis. The 863 GIF Tridiem (Gatan, Pleasanton, CA, United States) was used as an image filter. The EFTEM images were recorded with a slow-scan CCD camera model 794 (Gatan, Pleasanton, CA, United States); the images were evaluated using the Digital Micrograph software (Gatan, Pleasanton, CA, United States).

The XPS measurements were performed using a Phoibos 150 MCD9 hemispherical energy analyzer (SPECS Surface Nano Analysis GmbH, Berlin, Germany). The powder samples were carefully ground under a flow of argon gas, to keep the grain size as small as possible (charge balancing). The powder was then placed on a conductive carbon-containing adhesive carrier for TEM/REM measurements (Plano GmbH, Wetzlar, Germany) on the XPS holder and immediately introduced into the ultra-high vacuum (UHV) system with a base pressure of 2.5·10^−10^ mbar. MgK_α_ radiation (1253.69 eV, X-ray source XR-50, SPECS Surface Nano Analysis GmbH, Berlin, Germany) was used for excitation. To compensate for charging effects, all spectra were calibrated to the C 1s signal (284.8 eV). The Pd 3d detail spectra were measured with a 50 eV pass energy and the overview spectrum with a 100 eV pass energy. All detail spectra were background subtracted with the Shirley software package (2023-2025 © J. A. Hochhaus). The peak fitting routine was performed with Origin (OriginLab Corp., Northampton, MA, USA) using sets of gaussian curves.

*Diphenyl(2-(((3-(triethoxysilyl)propyl)thio)methyl)phenyl)phosphine (**1**)*: 639 mg (2.06 mmol) of (2-chloromethylphenyl)diphenylphosphine and 490 mg (2.06 mmol) 3-mercaptopropyltriethoxysilane were dissolved in 30 mL of CH_3_CN at r.t. and 848 mg (6.14 mmol) of K_2_CO_3_ were added, causing the suspension to turn yellow. The reaction mixture was stirred at 82 °C for 18 h and then filtered. The solvent was removed under vacuum. Yield: 1.04 g (99%) of a pale-yellow oil. Elemental analysis calcd. for C_28_H_37_O_3_PSSi (512.72 g/mol): C 65.59, H 7.27, S 6.25; found: C 65.35, H 7.31, S 6.12%. ^1^H NMR (400 MHz, CD_2_Cl_2_): δ 7.47–7.44 (m, 1H, H-8), 7.33–7.30 (m, 7H, H-9, H-15, H-16), 7.28–7.24 (m, 4H, H-14), 7.15 (t, ^3^*J*_HH_ = 7.5 Hz, 1H, H-10), 6.94–6.91 (m, 1H, H-11), 3.98 (d, ^4^*J*_HH_ = 1.7 Hz, 2H, H-6), 3.76 (q, ^3^*J*_HH_ = 7.4 Hz, 6H, H-2), 2.44 (t, ^3^*J*_HH_ = 7.3 Hz, 2H, H-5), 1.65–1.59 (m, 2H, H-4), 1.18 (t, ^3^*J*_HH_ = 6.9 Hz, 9H, H-1), 0.65–0.61 (m, 2H, H-3). ^13^C{^1^H} NMR (100.6 MHz, CD_2_Cl_2_): δ 144.3 (d, ^2^*J*_PC_ = 25.6 Hz, C-7), 137.7 (d, ^1^*J*_PC_ = 10.8 Hz, C-13), 136.7 (d, ^1^*J*_PC_ = 13.6 Hz, C-12), 134.8 (s, C-11), 134.3 (d, ^3^*J*_PC_ = 19.6 Hz, C-15), 130.3 (d, ^3^*J*_PC_ = 4.8 Hz, C-8), 129.6 (s, C-9), 129.1 (s, C1-6), 129.0 (d, ^2^*J*_PC_ = 6.8 Hz, C-14), 127.7 (s, C-10), 58.8 (s, C-2), 35.7 (s, C-5), 35.4 (d, ^3^*J*^PC^ = 24.8 Hz, C-6), 23.7 (s, C-4), 18.7 (s, C-1), 10.4 (s, C-3). ^31^P{^1^H} NMR (162.0 MHz, CD_2_Cl_2_): δ −17.2 (s).IR (ATR, cm^−1^): ṽ 3055w, 2975m, 2924w, 2883w, 1435m, 1072m, 955m, 741m.


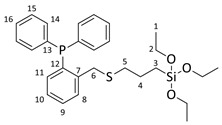


*Dichlorido[diphenyl(2-(((3-(triethoxysilyl)propyl)thio)methyl)phenyl)phosphino]palladium(II) (**2**)*: A solution of 1.82 g (3.54 mmol) of ligand **1** compound in 10 mL of CH_2_Cl_2_ was added to a solution of 679 mg (1.77 mmol) (CH_3_CN)_2_PdCl_2_ in 10 mL of CH_2_Cl_2_. The orange solution was stirred at r.t. for 15 h. It was then filtered and the solvent was removed under vacuum. The resulting yellow solid was washed three times with 10 mL of diethyl ether and twice with 10 mL of *n*-pentane. Yield: 1.21 g (99%) of a yellow solid. Elemental analysis calcd. for C_28_H_37_Cl_2_O_3_PPdSSi (690.03): C 48.74, H 5.40, S 4.65; found: C 48.68, H 5.20, S 4.55%. ^1^H NMR (400 MHz, CD_2_Cl_2_): δ 7.71–7.66 (m, 2H, H-16, H-20), 7.63–7.56 (m, 5H, H-11, H-14, H-18), 7.54–7.49 (m, 4H, H-15, H-19), 7.41–7.38 (m, 2H, H-8, H-10), 6.88–6.84 (m, 1H, H-9), 3.78 (q, ^3^*J*_HH_ = 6.95 Hz, 6H, H-2), 3.72 (d, ^3^*J*_HH_ = 6.46 Hz, 1H, H-6), 3.41 (d, ^3^*J*_HH_ = 13.4 Hz, 1H, H-6′), 3.16–3.09 (m, 1H, H-5), 2.69–2.65 (m, 1H, H-5′), 1.98–1.87 (m, 1H, H-4), 1.75–1.65 (m, 1H, H-4′), 1.19 (t, ^3^*J*_HH_ = 6.97 Hz, 9H, H-1), 0.78–0.70 (m, 1H, H-3), 0.64–0.57 (m, 1H, H-3′). Note: In some cases, it was not possible to assign the ^13^C NMR signals precisely due to signal overlap.^13^C{^1^H} NMR (100.6 MHz, CD_2_Cl_2_): δ 138.4 (d, ^2^*J*_PC_ = 12.2 Hz, C-7), 135.4 (d, *J*_PC_ = 12.2 Hz), 135.2 (d, *J*_PC_ = 12.2 Hz), 135.1 (s, C-9, C-16 or C-20), 134.3 (d, *J*_PC_ = 19.3 Hz, C14), 133.0 (d, *J*_PC_ = 3.0 Hz), 132.8 (d, *J*_PC_ = 7.9 Hz), 132.6 (s, C-8, C-11 or C-18), 130.4 (d, ^3^*J*_PC_ = 8.8 Hz, C-10), 129.7 (d, ^3^*J*_PC_ = 11.4 Hz, C-15), 129.3 (d, ^3^*J*_PC_ = 11.8 Hz, C-19), 127.2 (d, ^1^*J*_PC_ = 10.2 Hz, C-17 or C13), 126.7 (d, ^1^*J*_PC_ = 11.6 Hz, C-17 or C-13), 124.7 (d, ^1^*J*_PC_ = 49.7 Hz, C12), 59.0 (C-2), 40.4 (C-5), 34.4 (d, ^3^*J*_PC_ = 12.0 Hz, C-6), 22.6 (C-4), 18.7 (C-1), 10.4 (C-3). ^31^P{^1^H} NMR (162.0 MHz, CD_2_Cl_2_): δ 25.7 (s). IR (ATR, cm^−1^): ṽ 3057w, 2969m, 2925w, 2879w, 1434m, 1066s, 950m, 753m.


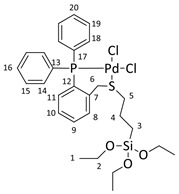


*Carbonylchloridobis[diphenyl(2-(((3-(triethoxysilyl)propyl)thio)methyl)phenyl)phosphino]rhodium(I) (**3**) and carbonylchloridobis[diphenyl(2-(((3-(triethoxysilyl)propyl)thio)methyl)phenyl)phosphino]rhodium(I) (**3′**) (see Scheme 2)*: 151 mg (0.38 mmol) of [Rh(CO)_2_Cl]_2_ dissolved in 6 mL of CH_2_Cl_2_ was added to 795 mg (1.55 mmol) of ligand **1** dissolved in 2 mL of CH_2_Cl_2_. The dark orange solution was then stirred at r.t. for 15 h. The solvent was removed under vacuum and the resulting solid was washed three times with 10 mL of diethyl ether. Yield: 296 mg (56%) of a yellow solid. Elemental analysis calcd. for C_57_H_74_ClO_7_P_2_RhS_2_Si_2_ (80%) + C_29_H_37_ClO_4_PRhSSi (20%) (1191.81/679.09): C 56.68, H 6.16, S 5.30; found: C 56.83, H, 6.19, S 5.13%. Note: The numbering of the NMR data of **3′** is according to the figure above. ^1^H NMR (400 MHz, CD_2_Cl_2_): δ 7.65–7.30 (m, 11H, H-11, H14-16, H18-20), 7.13–7.10 (m, 2H, H-8, H-10), 6.30 (s, 1H, H-9), 3.81–3.73 (m, 7H, H-2, H-6), 3.44–3.40 (m, 1H, H-6′), 2.88–2.80 (m, 1H, H-5), 2.31–2.24 (m, 1H, H-5′), 1.66–1.58 (m, 1H, H-4), 1.48–1.38 (m, 1H, H-4′), 1.22–1.16 (m, 9H, H-1), 0.58–0.50 (m, 1H, H-3), 0.45–0.37 (m, 1H, H-3′). ^31^P{^1^H} NMR (162.0 MHz, CD_2_Cl_2_): δ 25.7 (d, ^1^*J*_RhP_ = 124.7 Hz). IR (ATR, cm^−1^): ṽ 3054w, 2973w, 2919w, 2886w, 1971s (ν_C=O_), 1435m, 1071s, 958m, 755m.


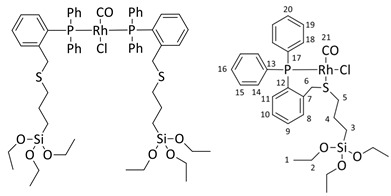


*General procedure for the immobilization of the phosphine complexes:* The support material and the transition metal complex were suspended in toluene. The yellow suspension was stirred at 110 °C for 24 h. After cooling to room temperature, the solid was separated by centrifugation (5000 rpm, 2 min) and washed twice with 10 mL of acetone and three times with 10 mL of ethanol. The catalyst was dried overnight at 80 °C under vacuum and then stored under an atmosphere of inert gas (N_2_).

*Synthesis of material **4a**:* 1.24 g of SBA-15, 300 mg (0.44 mmol) of complex **2**, 20 mL of toluene. Yield: 1.36 g of a yellow solid. Elemental analysis, found: C 14.00, H 2.61, S 0.86, Pd 2.10%. Specific surface: 470 m^2^·g^−1^, specific pore volume: 1.14 cm^3^·g^−1^, mean pore diameter: 5.36 nm. IR (ATR, cm^−1^): ṽ 3391w, 2979w, 1439w, 1021s, 949s, 802. ^13^C CP-MAS NMR (126 MHz): δ 130.2 (C_Ar_), 72.0 (template), 67.5 (template), 56.4 (C2), 37.6 (C5, C6), 18.8 (C4), 13.6 (C1), 8.4 (C3). ^29^Si CP-MAS NMR (99 MHz): δ −46.6 (T_1_), −56.4 (T_2_), −92.7 (Q_2_), −100.9 (Q_3_), −110.8 (Q_4_). ^31^P CP MAS NMR (202.49 MHz): δ 26.9.

*Synthesis of material **5a**:* 1.95 g of silica gel, 470 mg (0.68 mmol) of silica complex **2**, 20 mL of toluene. Yield: 2.10 g of a yellow solid. Elemental analysis, found: C 9.39, H 1.33, S 1.04, Pd 2.20%. Specific surface: 250 m^2^·g^−1^, specific pore volume: 0.63 cm^3^·g^−1^ mean pore diameter: 5.71 nm. IR (ATR, cm^−1^): ṽ 2962w, 1440m, 1036s, 921m, 750w. ^13^C CP-MAS NMR (126 MHz): δ 130.5 (C_Ar_), 57.4 (C2), 36.5 (C5, C6), 20.7 (C4), 15.0 (C1), 9.7 (C3). ^29^Si CP-MAS NMR (99 MHz): δ −47.3 (T_1_), −56.3 (T_2_), −99.6 (Q_3_), −110.3 (Q_4_). ^31^P CP MAS NMR (202.49 MHz): δ 27.5. ^31^P CP MAS NMR (202.49 MHz): δ 25.7.

*Synthesis of material **6a**:* 690 mg of SBA-15, 289 mg (0.24 mmol) of the mixture of complexes **3** and **3′** (see above), 20 mL of toluene. Yield: 634 mg of a dark yellow solid. Elemental analysis, found: C 19.80, H 2.87, S: 1.64, Rh 2.30%. Specific surface: 450 m^2^·g^−1^, specific pore volume: 0.94 cm^3^·g^−1^ mean pore diameter: 5.55 nm. IR (ATR, cm^−1^): ṽ 3333 w, 2978w, 1437w, 1048s, 1070s, 958m, 796m, 750w. ^13^C CP-MAS NMR (126 MHz): δ 130.1 (C_Ar_), 73.7 (template), 68.8 (template), 57.4 (C2), 36.7 (C5, C6), 21.1 (C4), 15.4 (C1), 8.9 (C3). ^29^Si CP-MAS NMR (99 MHz): δ −53.9 (T_2_), −61.4 (T_3_), −94.9 (Q_2_), −103.5 (Q_3_), −110.2 (Q_4_). ^31^P CP MAS NMR (202.49 MHz): δ 34.7.

*General procedure for reducing **4a–6a** with hydrogen gas:* Each of the materials (**4a**–**6a**) was suspended in toluene. The mixture was placed in a BR-100 high-pressure reactor (Berghof, Eningen, Germany), which was sealed. It was then purged three times with 15 bar of hydrogen gas, after which 30 bar of hydrogen pressure was applied. Reduction of the material was carried out at this pressure and at 100 °C. The solid was then separated by centrifugation at 5000 rpm for 2 min, washed twice with 10 mL of acetone and three times with 10 mL of ethanol, dried for 12 h at 80 °C and stored under an inert gas atmosphere (N_2_).

*Synthesis of material **4b**:* 706 mg of **4a**, 5 mL of toluene, 24 h. Yield: 684 mg of a beige solid. Elemental analysis, found: C 12.94, H 2.46, S 0.66, Pd 2.49%. Specific surface: 530 m^2^·g^−1^, specific pore volume: 1.15 cm^3^·g^−1^ mean pore diameter: 5.08 nm. IR (ATR, cm^−1^): ṽ 3364w, 2950w, 1439w, 1047s, 956m, 799m. ^13^C CP-MAS NMR (126 MHz): δ 130.4 (C_Ar_), 72.6 (template), 68.5 (template), 57.2 (C2), 36.5 (C5, C6), 21.2 (C4), 14.7 (C1), 9.6 (C3). ^29^Si CP-MAS NMR (99 MHz): δ −58.7 (T_2_), −102.2 (Q_3_), −110.9 (Q_4_). ^31^P CP MAS NMR (202.49 MHz): δ 27.5. ^31^P CP MAS NMR (202.49 MHz): δ 27.5.

*Synthesis of material **5b**:* 1.19 g of **5a**, 5 mL of toluene, 24 h. Yield: 1.11 g of a brown solid. Elemental analysis, found: C 7.49, H 1.29, S: 0.83, Pd 2.40%. Specific surface: 310 m^2^·g^−1^, specific pore volume: 0.70 cm^3^·g^−1^ mean pore diameter: 5.65 nm. IR (ATR, cm^−1^): ṽ 3385w, 2946w, 1645w, 1436w, 1120s, 1050s, 998s, 960m, 790m. ^13^C CP-MAS NMR (126 MHz): δ 130.3 (C_Ar_), 58.2 (C2), 37.3 (C5, C6), 23.1 (C4), 16.2 (C1), 10.9 (C3). ^29^Si CP-MAS NMR (99 MHz): δ −57.6 (T_2_), −100.6 (Q_3_), −110.5 (Q_4_). ^31^P CP MAS NMR (202.49 MHz): δ 26.6.

*Synthesis of material **6b**:* 429 mg of **6a**, 5 mL of toluene, 5 h. Yield: 358 mg of a brown solid. Elemental analysis, found: C 16.87, H 2.51, S 2.05, Rh 2.50%. Specific surface: 490 m^2^·g^−1^, specific pore volume: 0.99 cm^3^·g^−1^ mean pore diameter: 4.75 nm. IR (ATR, cm^−1^): ṽ 3350w, 2958w, 1638w, 1438w, 1088s, 1041s, 958m, 794m, 715w. ^13^C CP-MAS NMR (126 MHz): δ 130.3 (C_Ar_), 73.6 (template), 69.7 (template), 58.4 (C2), 39.6 (C5, C6), 21.5 (C4), 15.6 (C1), 10.5 (C3). ^29^Si CP-MAS NMR (99 MHz): δ −59.1 (T_2_), −66.5 (T_3_), −94.0 (Q_2_), −102.8 (Q_3_), −110.8 (Q_4_). ^31^P CP MAS NMR (202.49 MHz): δ 35.4.

*Catalytic hydrogenation of 2-cyclohexen-1-one at 15 bar:* 96 mg (1 mmol) of 2-cyclohexen-1-one, 60 mg of *n*-decane (internal GC standard), 0.2 mol-% of palladium (as content in the catalyst material) and 1 mL of toluene were weighed into a 20 mL round-bottom flask equipped with a magnetic stirring bar (PTFE). Then the round-bottom flask was placed in a BR-100 high-pressure reactor (Berghof) and the reactor was closed. After three gas exchanges with 15 bar of hydrogen, the final pressure was set and the catalysis started. For reactions at elevated temperatures, the reactor was preheated. The stirring speed was set to 500 rpm. After a reaction time of 2 h, the autoclave was opened and a sample was taken for GC analysis.

*Catalytic olefin hydrogenation at room temperature and under ambient pressure:* These reactions were carried out in 15 mL crimp-top vials sealed with a Teflon^®^-coated, aluminum cap which was fitted with a rubber septum. The weighing of the catalyst, the olefin, the solvent cyclohexane and the GC standard, was carried out in a similar manner as described above. Then the vial was closed and hydrogen was introduced using a hydrogen filled balloon equipped with a cannula that was inserted into the vial via the septum. Samples for GC analysis were taken using a syringe.

*Catalyst recycling studies:* The catalysts **4b**–**6b** were recycled over ten cycles applying 50 mg of catalyst in the first experiment. The experiments were carried out at ambient hydrogen pressure, using a balloon filled with hydrogen as described above, and at room temperature in 10 mL centrifuge glass tubes. 96 mg (1 mmol) of 2-cyclohexen-1-one, 60 mg of *n*-decane (internal GC standard) were applied. After addition of all solid and liquid components, test tube was sealed with a rubber septum. The tube was purged with hydrogen for 30 s and then connected via a cannula to a hydrogen balloon to initiate the catalysis. The stirring speed was set to 500 rpm. After 1 h, the hydrogen atmosphere was removed, the suspension was centrifuged and a sample was taken from the liquid phase. The remaining reaction solution was decanted, and the catalyst residues were washed twice with 10 mL of acetone and once with 10 mL of ethanol. The catalyst was dried in the air at 80 °C for 12 h and then reused for the next cycle.

## 4. Conclusions

The surface immobilization of palladium and rhodium on SBA-15 is made possible by a simple process leading to the formation of a triethoxysilyl-functionalized triphenylphosphine ligand and its coordination to appropriate palladium and rhodium precursor complexes. The transition metal sites become catalytically active for olefin hydrogenation only after (partial) reduction with dihydrogen. On the one hand, the phosphine sites on the surface may stabilize metal nanoparticles against agglomeration and growth, and on the other hand, they may reduce the polarity of the silica surface. We are working towards further optimizing the surface properties of silica supports. The importance of this is still underestimated. Element mapping (TEM) and XPS analysis of one of the palladium catalysts clearly demonstrate the presence of multiple palladium species on the surface. There is undoubtedly still palladium(II) present even after tenfold reuse of the catalyst, indicating an incomplete of the metal.

We are now planning to investigate in more detail whether the ratio of palladium(0) to palladium(II) influences the activity of the catalyst. One possibility is that catalytic hydrogenation only occurs at the palladium(0) sites, or even only at the palladium(0) nanoparticles formed during the activation process of the catalyst. Alternatively, the palladium(II) species that are still present on the surface may play a positive role in enhancing the activity of the catalyst through collaborative effects with palladium(0) species. Alternatively, immobilizing the phosphine ligand significantly reduces the number of Si-OH groups on the pore surface, thereby decreasing surface polarity. This influences the hydration of the surface and the diffusion of the substrate and solvent, ultimately affecting the catalyst’s activity.

## Figures and Tables

**Table 1:** Elemental analyses of materials **4a,b**, **5a,b**, and **6a,b**.

Material	C/%	H/%	S/%	Metal Content
**4a**	14.00	2.61	0.86	2.10
**4b**	12.94	2.46	0.66	2.49
**5a**	9.39	1.33	1.04	2.20
**5b**	7.49	1.29	0.83	2.40
**6a**	19.80	2.87	1.64	2.30
**6b**	16.87	2.51	2.05	2.50

**Table 2:** Specific surfaces, total pore volumes and mean pore diameters of materials **4a,b**, **5a,b**, and **6a,b**.

Material	Spec. Surface Area/m^2^/g	V_P_/cm^3^/g	d_P_/nm
**4a**	470	1.14	5.4
**4b**	530	1.15	5.1
**5a**	250	0.63	5.7
**5b**	310	0.70	5.7
**6a**	450	0.94	5.6
**6b**	490	0.99	4.8

**Table 3:** Hydrogenation of a series of olefins with catalysts **4b**–**6b**.

Entry	Olefin	Product	4b	5b	6b
			Yield ^[a,b]^/%		
1	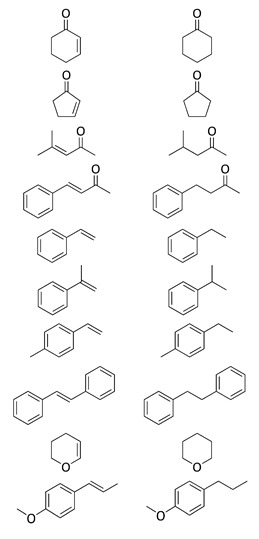		98	69/(>99) ^[c]^	>99
2			17/(>99) ^[c]^	9/(21) ^[c]^	42/(>99) ^[c]^
3			6/(20) ^[c]^	3/(5) ^[c]^	>99
4			4/(8) ^[c]^	3/(20) ^[c]^	3/(17) ^[c]^
5			>99	69/(>99) ^[c]^	53/(>99) ^[c]^
6			>99	>99	>99
7			>99	>99	10/(38) ^[c]^
8			43/(>99) ^[c]^	25/(>99) ^[c]^	41/(>99) ^[c]^
9			8/(43) ^[c]^	59/(>99) ^[c]^	95/(>99) ^[c]^
10			>99	81/(>99) ^[c]^	6/(66) ^[c]^

^[a]^ reaction conditions: r.t., 1 bar H_2_, 1 mmol of substrate, 2 mL of cyclohexane, 0.2 mol-% Pd/Rh, 5 h, ^[b]^ determined by GC-FID, ^[c]^ after 24 h.

## Data Availability

Experimental data can be obtained from the corresponding authors.

## Supplementary Materials

The following supporting information can be downloaded at: https://www.mdpi.com/article/10.3390/molecules31152601/s1.

## Abbreviations

The following abbreviations are used in this manuscript:

CP MAS NMR	Cross-polarization, magic-angle spinning NMR spectroscopy
EDX	Energy-dispersive X-ray spectroscopy
EFTEM	Energy-filtered transmission electron microscopy
GC-FID	Gas chromatography coupled with a flame ionization detector
IR	Infrared spectroscopy
NMR	Nuclear magnetic resonance spectroscopy
SEM	Scanning electron microscopy
SBA-15	Santa Barbara material no. 15
TEM	Transmission electron microscopy
XPS	X-ray photon spectroscopy
