# Supplementary material for: A Simple Access to Highly Active Heterogeneous Hydrogenation Catalysts
Source: Molecules. 2026 Jul 25;31(15):2601. doi: 10.3390/molecules31152601 (PMC13468633; doi:10.3390/molecules31152601)
Supplement: Supplementary file 1 [file molecules-31-02601-s001.zip › molecules-4425003-supplementary.pdf]

NMR spectra

NMR spectra of 1-(2-(diphenylphosphino)phenyl)-*N,N*-dimethylmethanamine

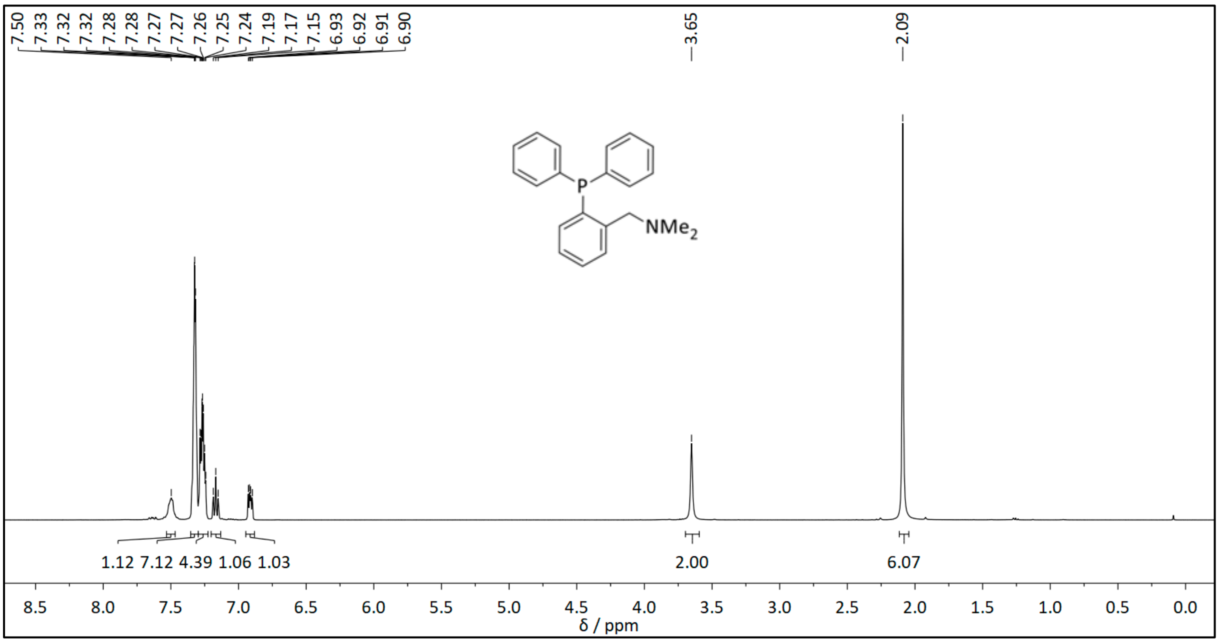

Figure S1. *^{1}H NMR spectrum of 1-(2-(diphenylphosphino)phenyl)-N,N-dimethylmethanamine.*

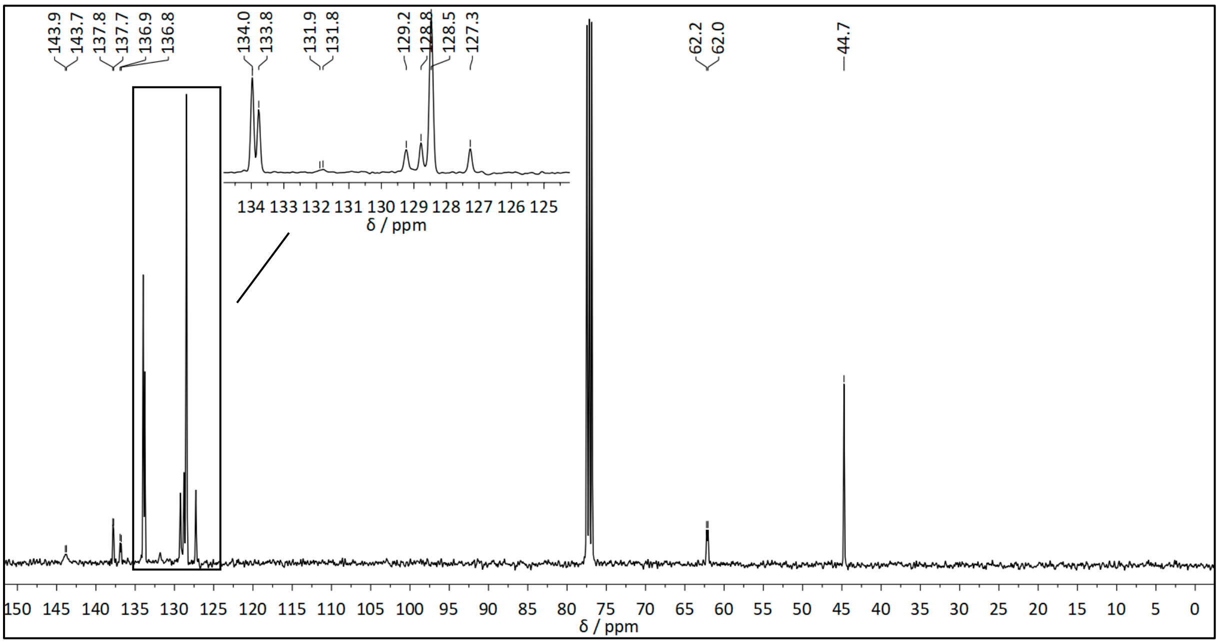

Figure S2. *^{13}C NMR spectrum of 1-(2-(diphenylphosphino)phenyl)-N,N-dimethylmethanamine.*

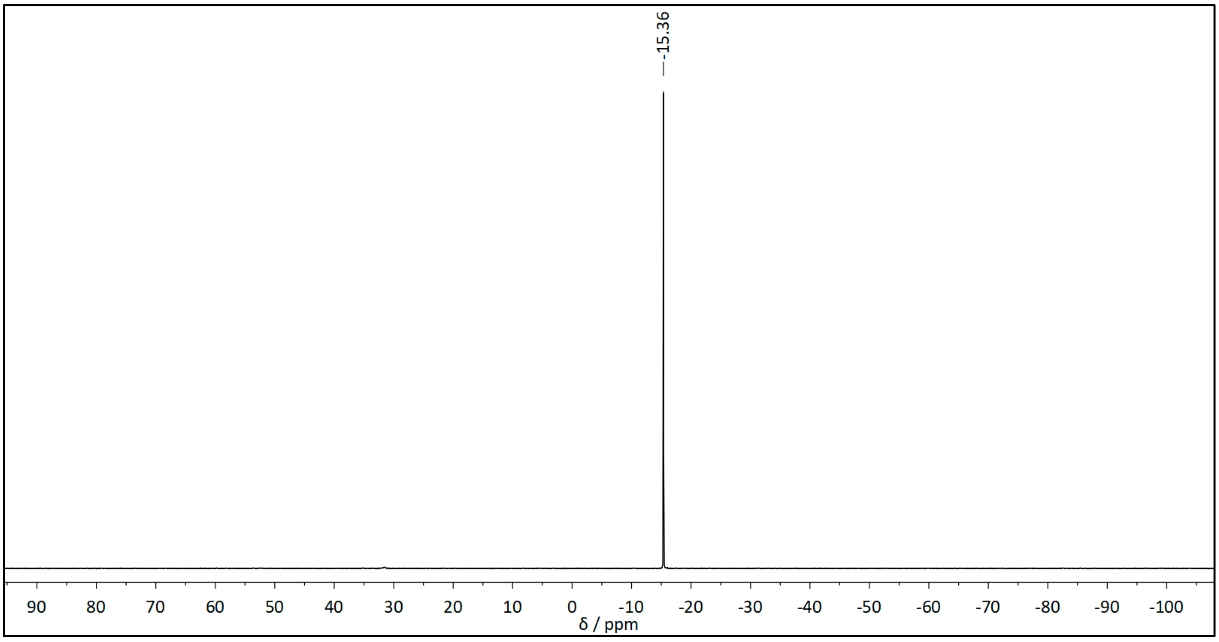

Figure S3. *^{31}P NMR spectrum of 1-(2-(diphenylphosphino)phenyl)-N,N-dimethylmethanamine.*

NMR spectra of (2-(chloromethyl)phenyl)diphenylphosphine

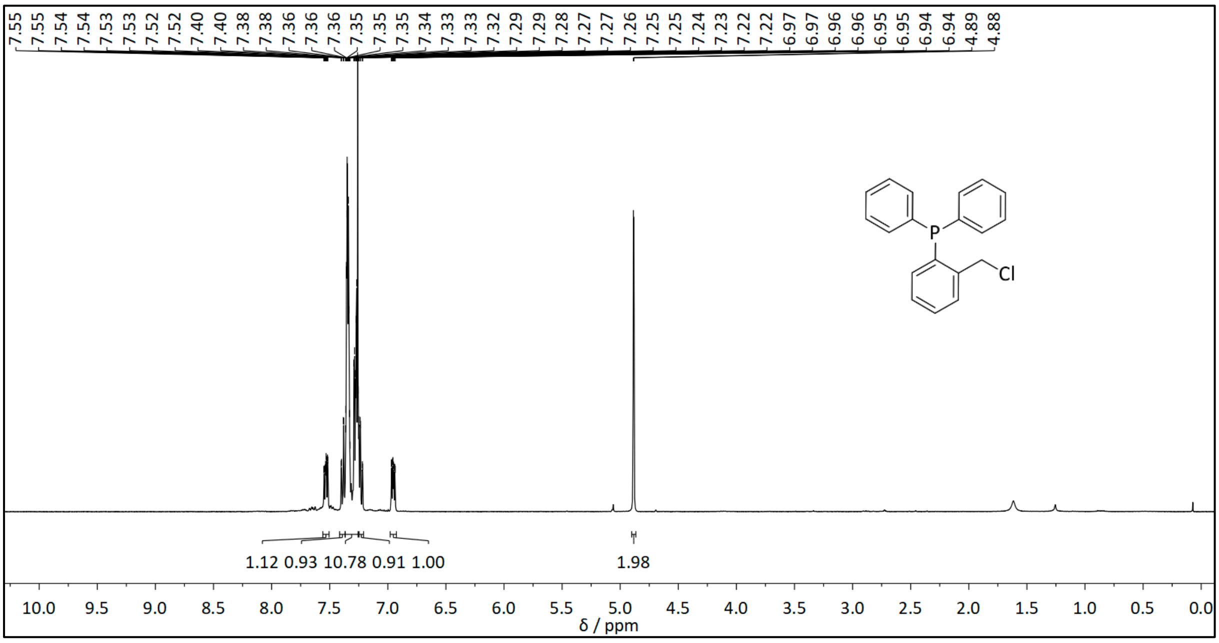

Figure S4. *^{1}H NMR spectrum of (2-(chloromethyl)phenyl)diphenylphosphine.*

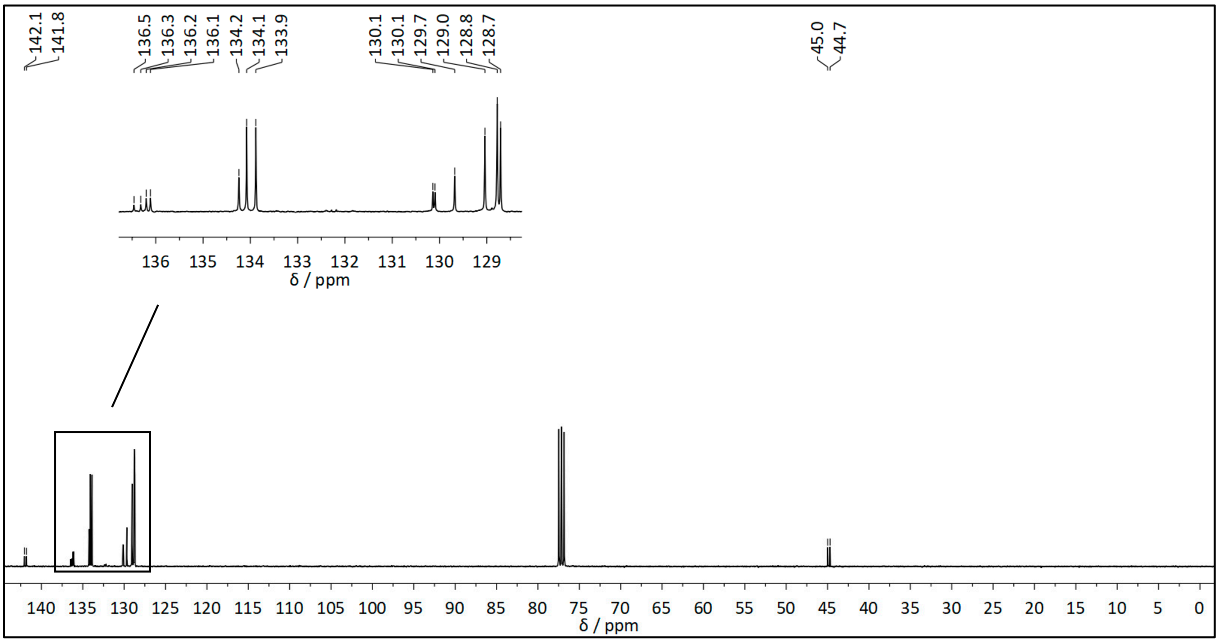

Figure S5. *^{13}C NMR spectrum of (2-(chloromethyl)phenyl)diphenylphosphine.*

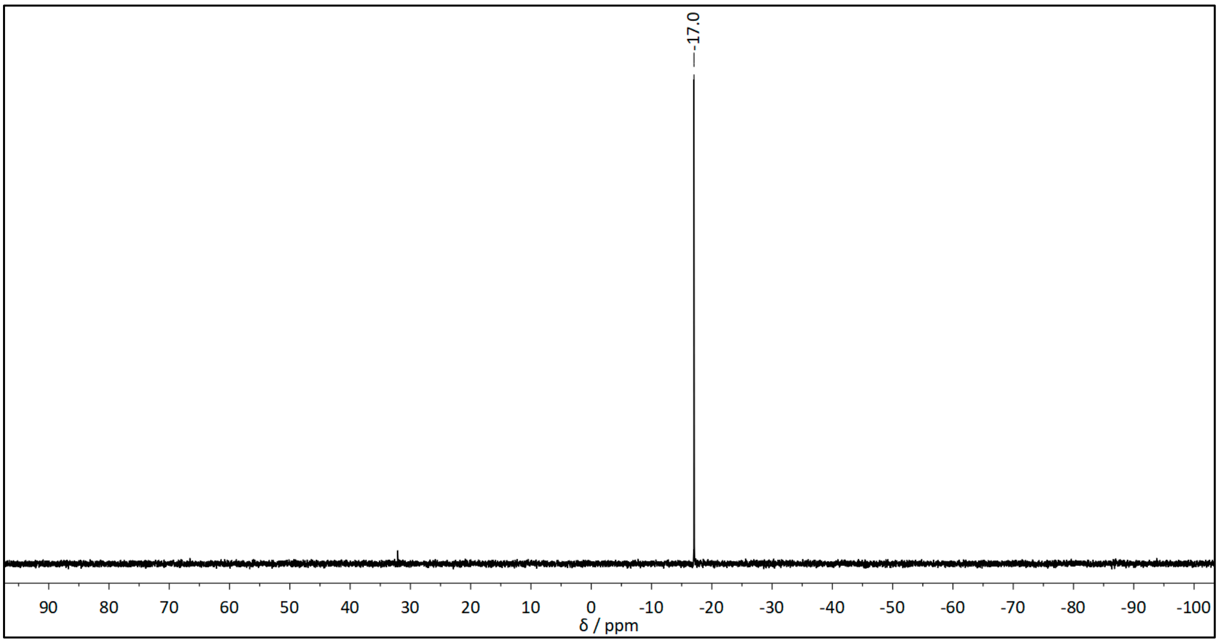

Figure S6. *^{31}P NMR spectrum of (2-(chloromethyl)phenyl)diphenylphosphine.*

NMR spectra of ligand **1**

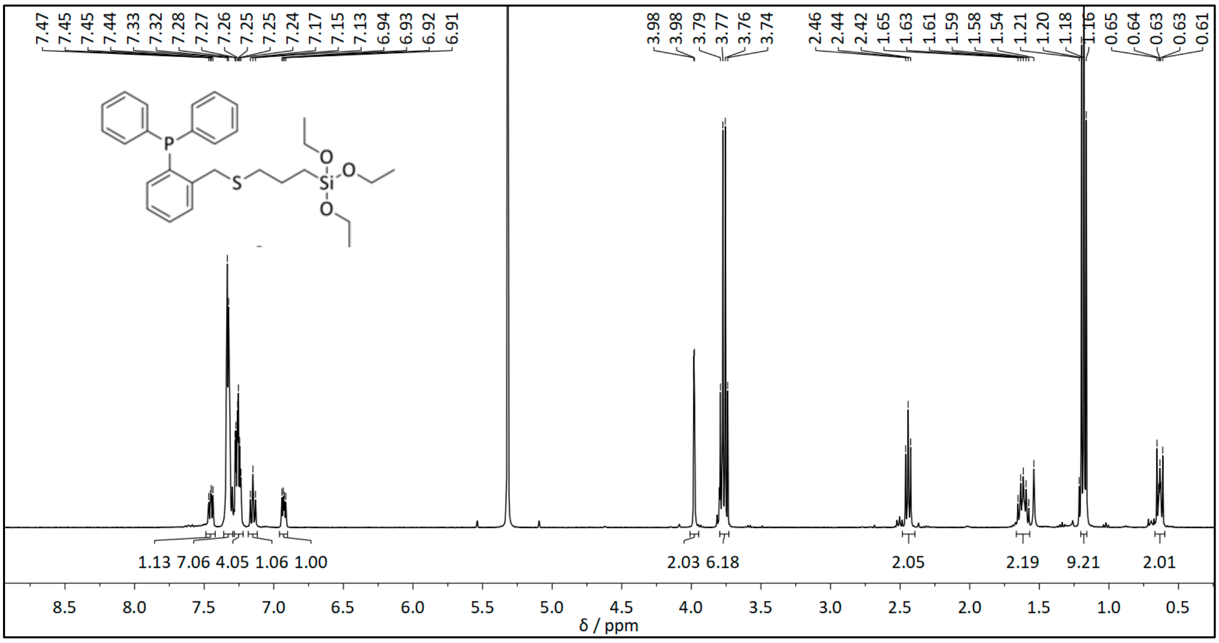

Figure S7. *^{1}H NMR spectrum of ligand* ***1****.*

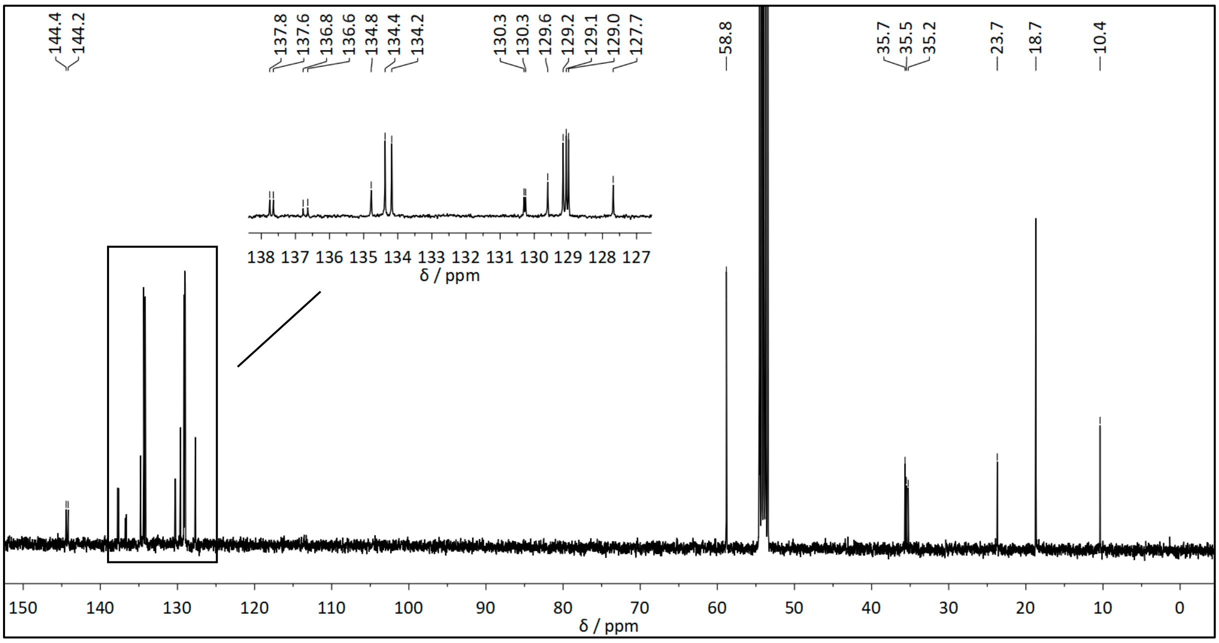

Figure S8. *^{13}C NMR spectrum of ligand* ***1****.*

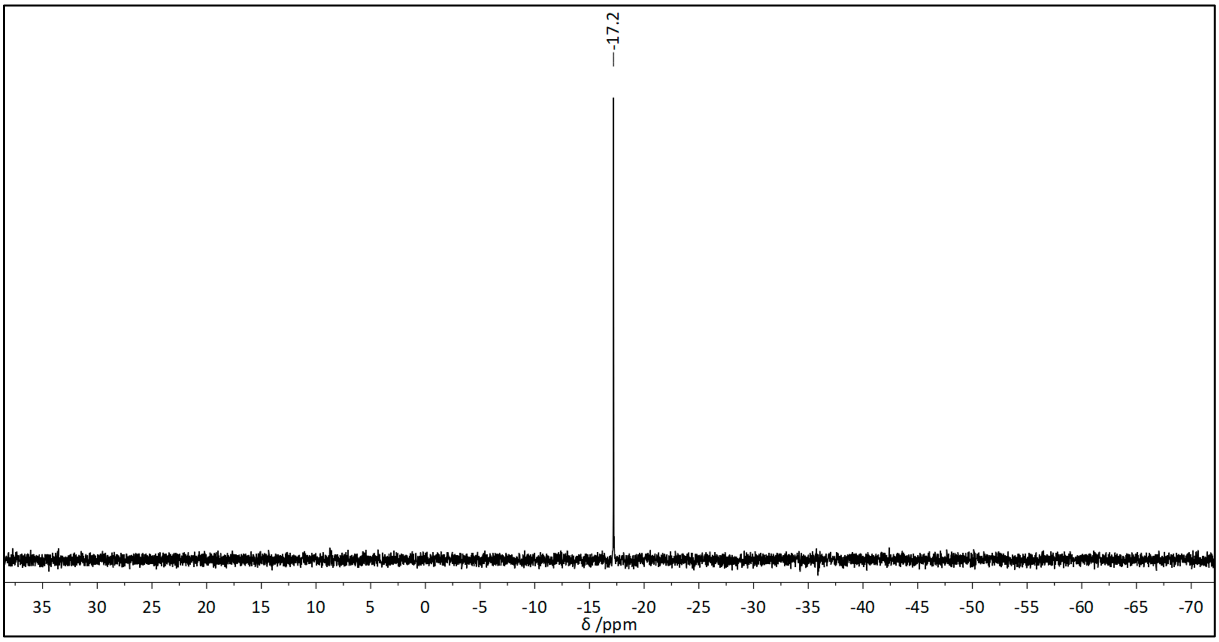

Figure S9. *^{31}P NMR spectrum of ligand* ***1****.*

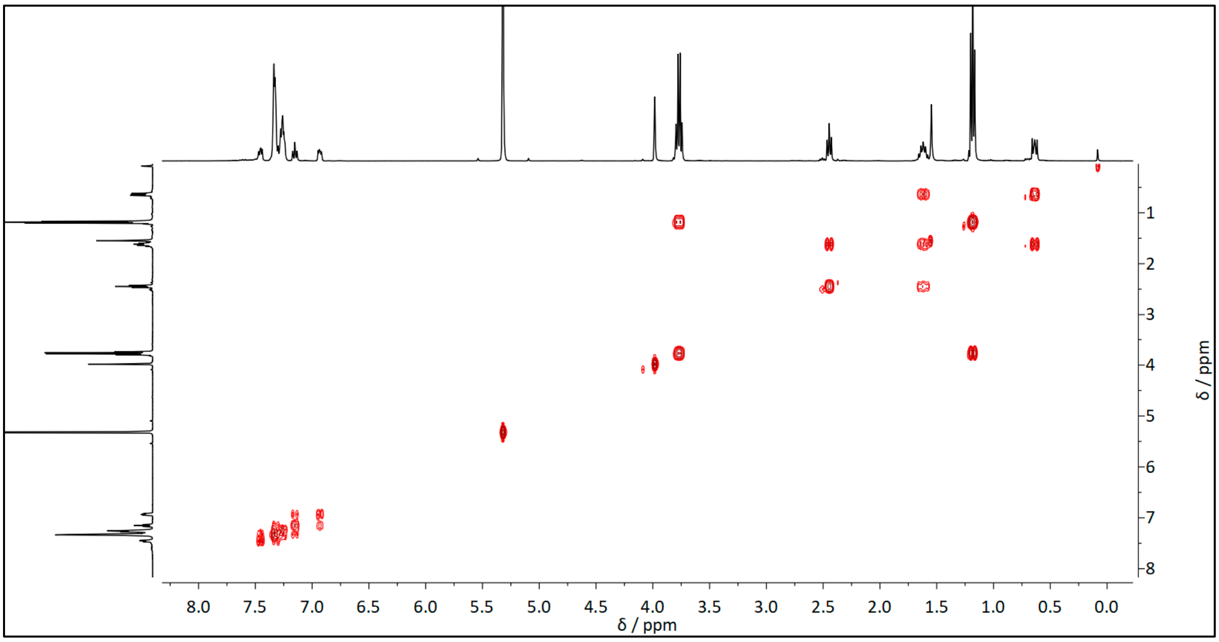

Figure S10. *H,H COSY spectrum of ligand* ***1****.*

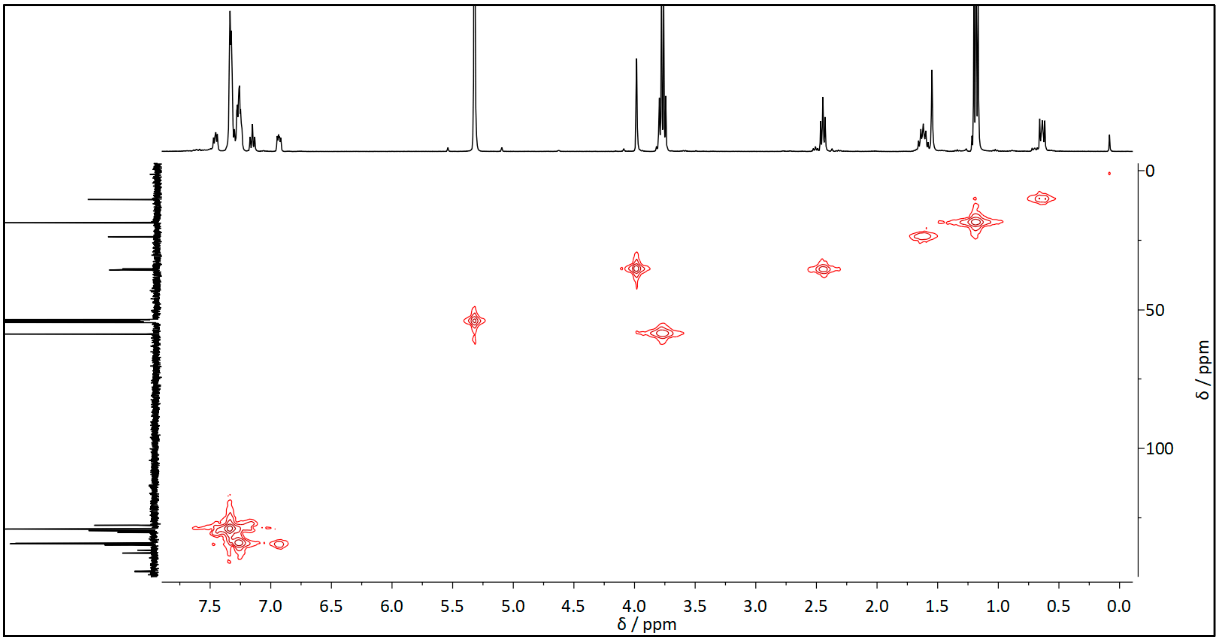

Figure S11. *HSQC spectrum of ligand* ***1***.

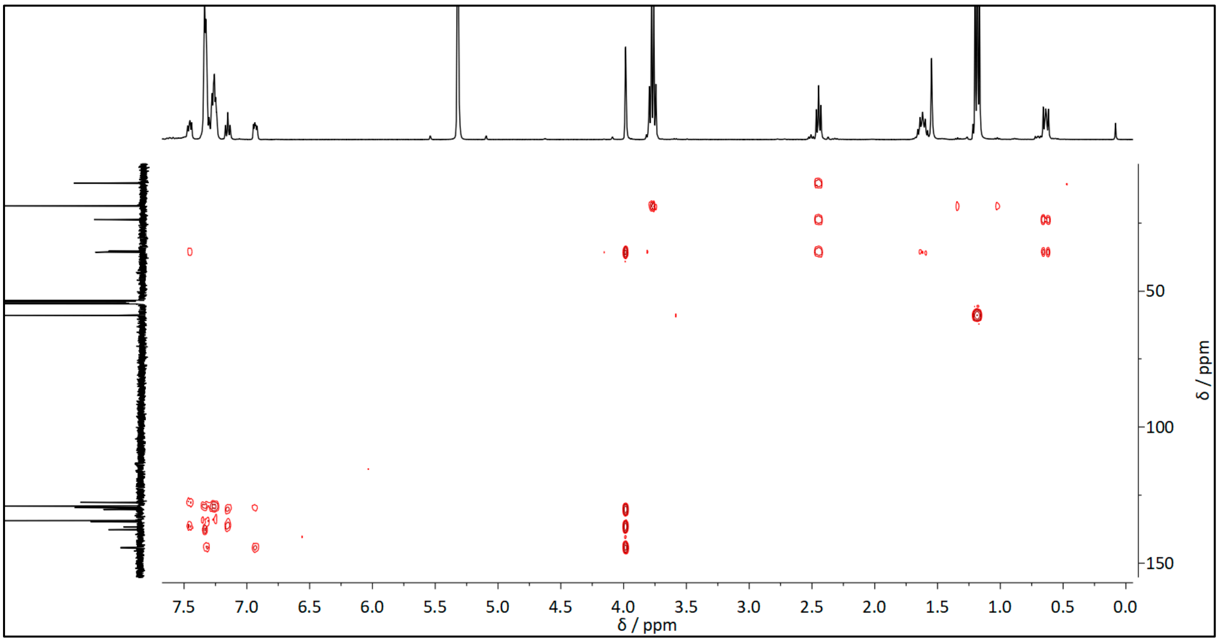

Figure S12. *HMBC spectrum of ligand* ***1***.

NMR spectra of palladium complex **2**

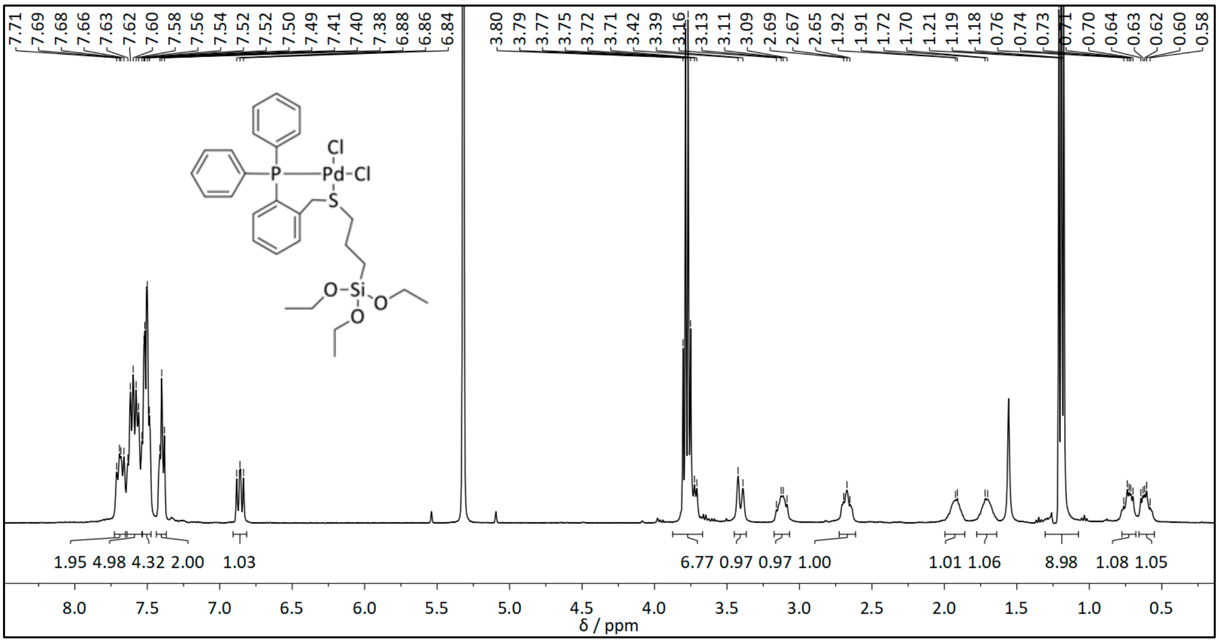

Figure S13. *^{1}H NMR spectrum of complex **2**.*

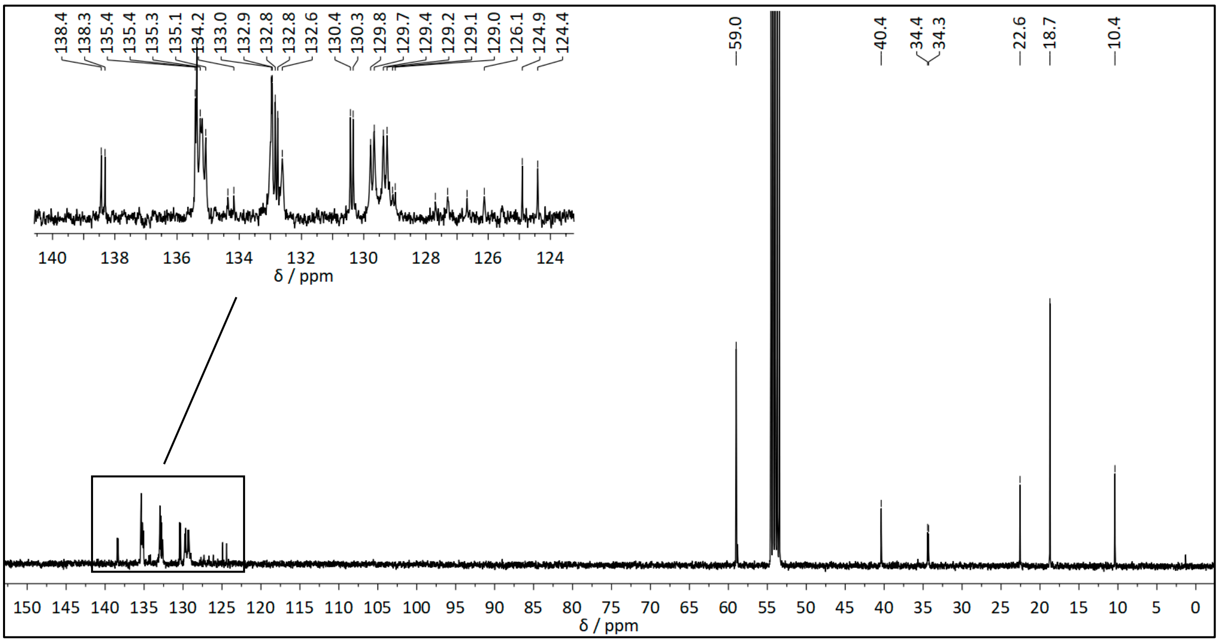

Figure S14. *^{13}C NMR spectrum of complex **2**.*

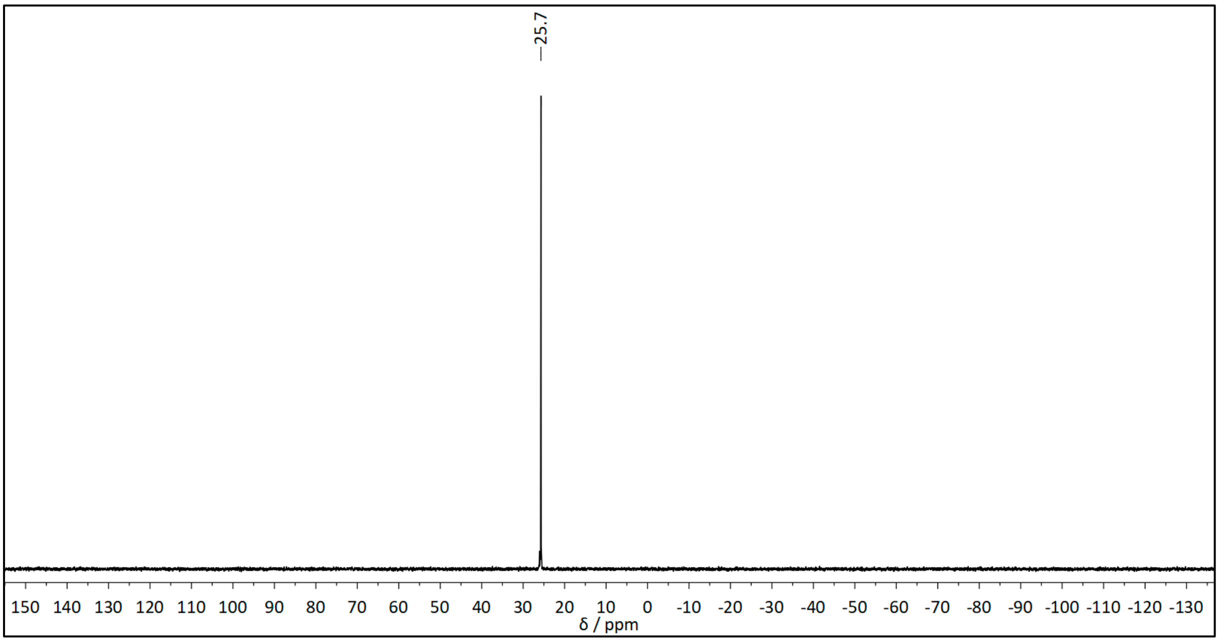

Figure S15. *^{31}P NMR spectrum of complex **2**.*

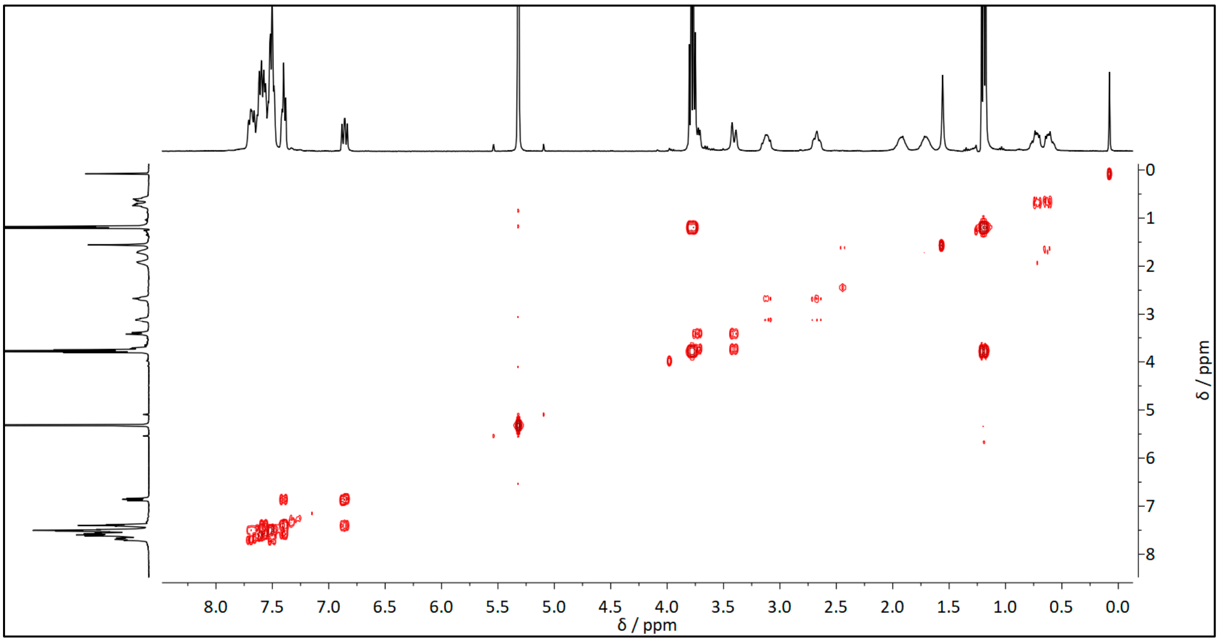

Figure S16. *H,H COSY spectrum of complex **2**.*

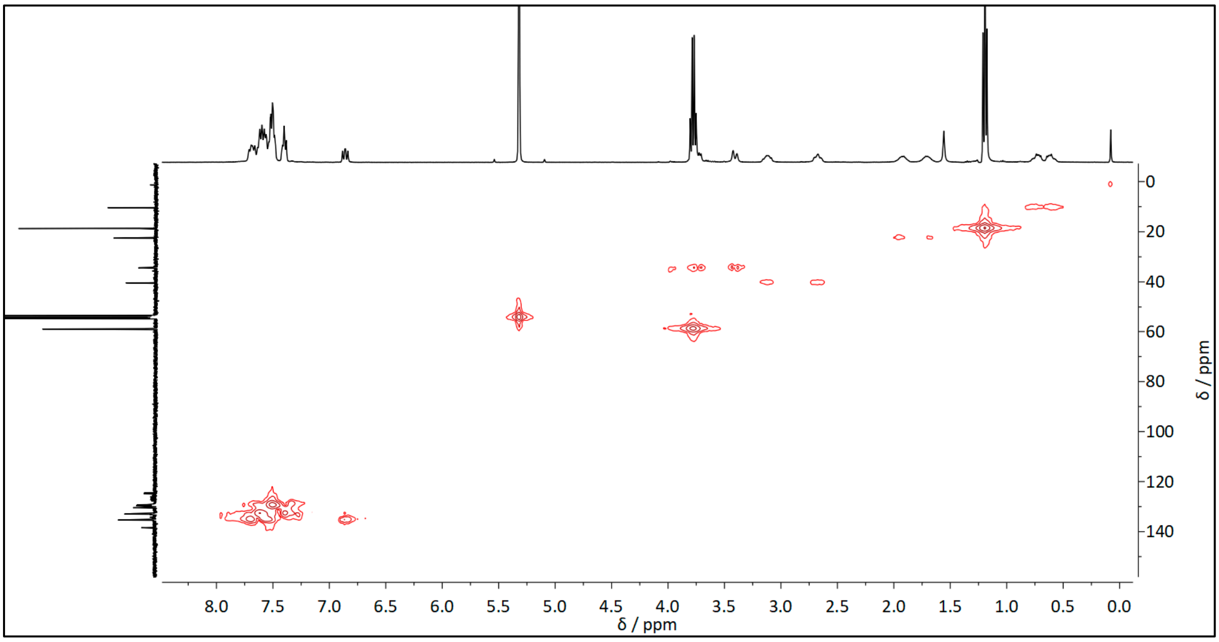

Figure S17. *HSQC spectrum of complex* ***2.***

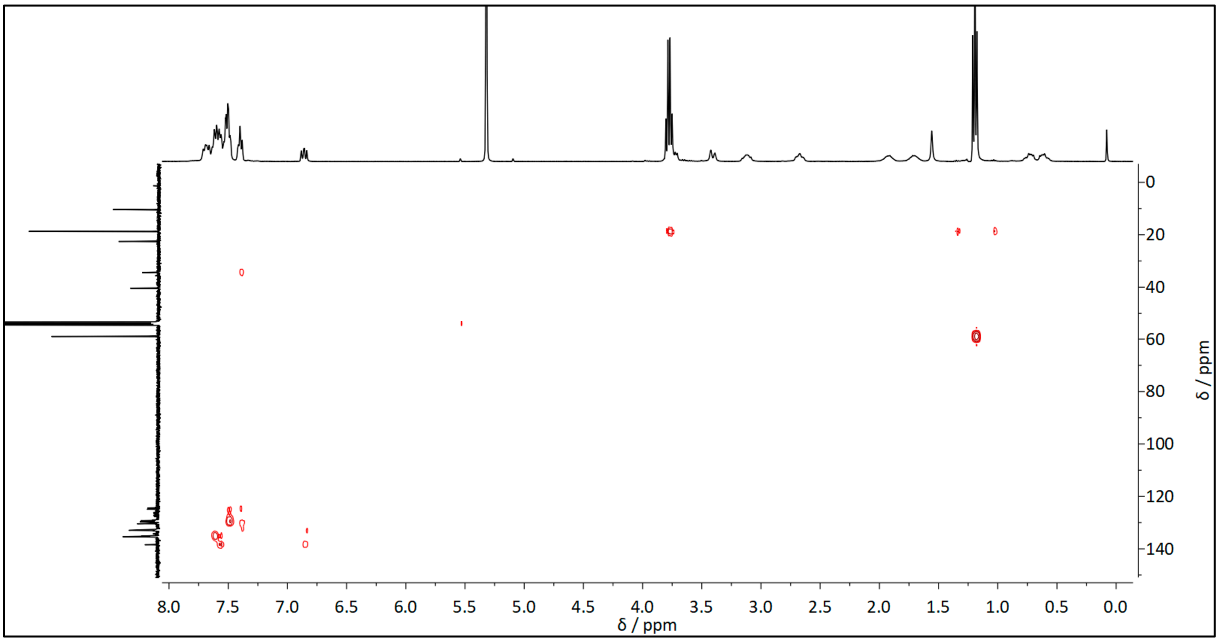

Figure S18. *HMBC spectrum of complex* ***2.***

NMR spectra of the mixture of the rhodium complexes **3** and **3'**

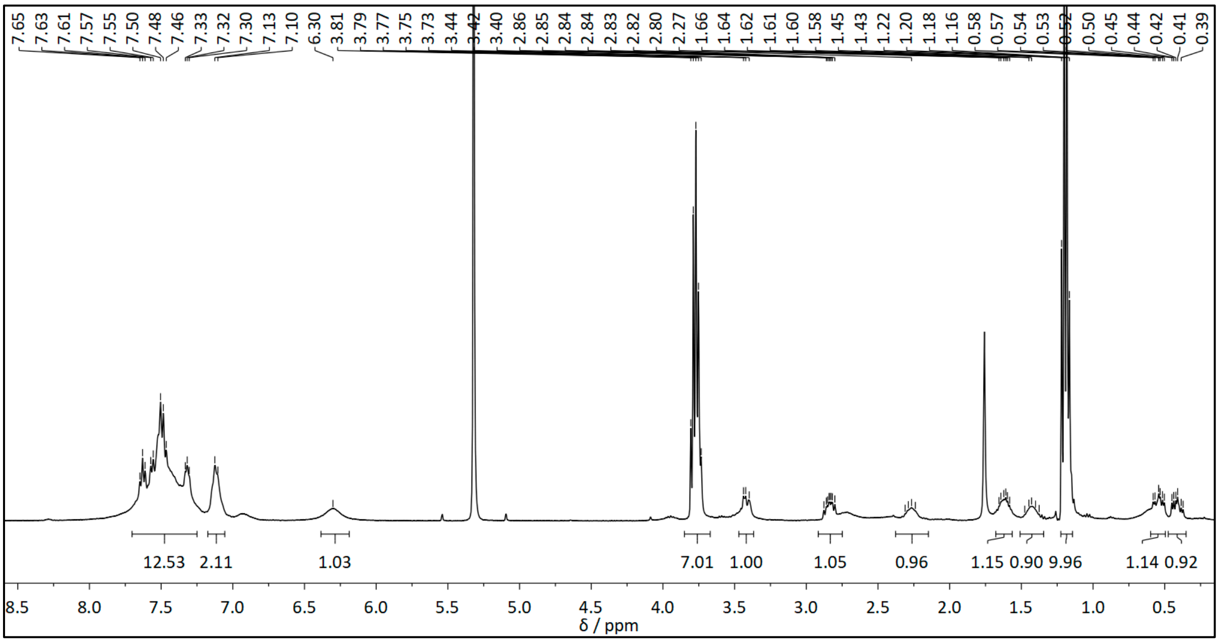

Figure S19. *^{1}H NMR spectrum of the mixture of the rhodium complexes **3** and **3'**.*

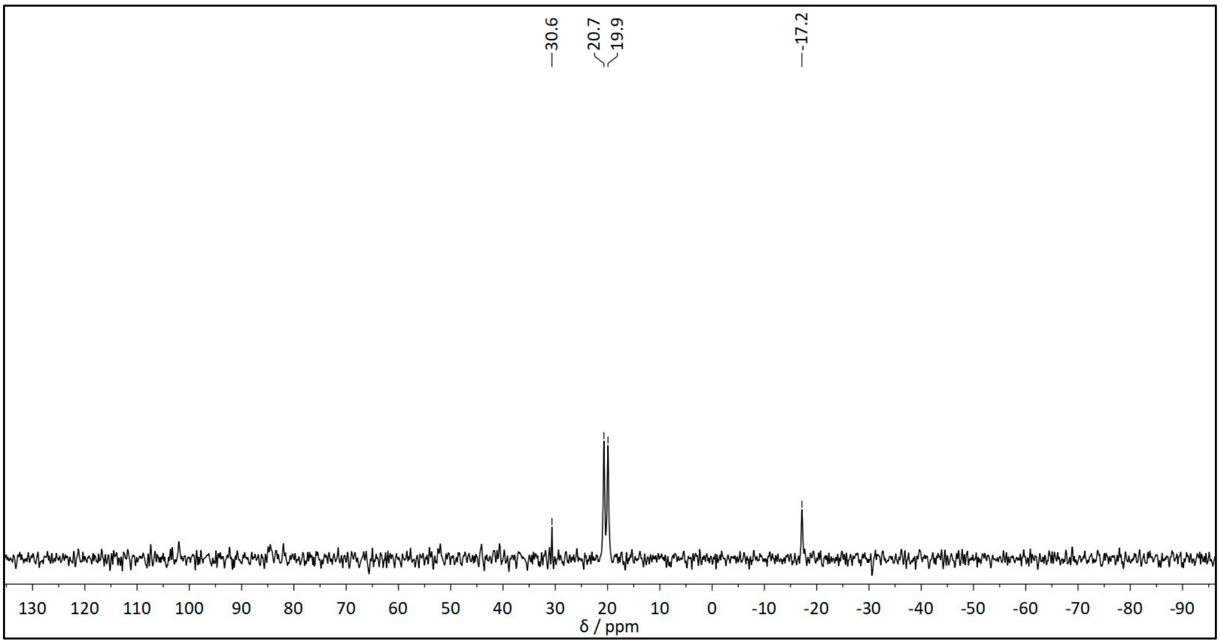

Figure S20. *^{31}P NMR spectrum of the mixture of the rhodium complexes **3** and **3'**.*

NMR spectra of material **4a**

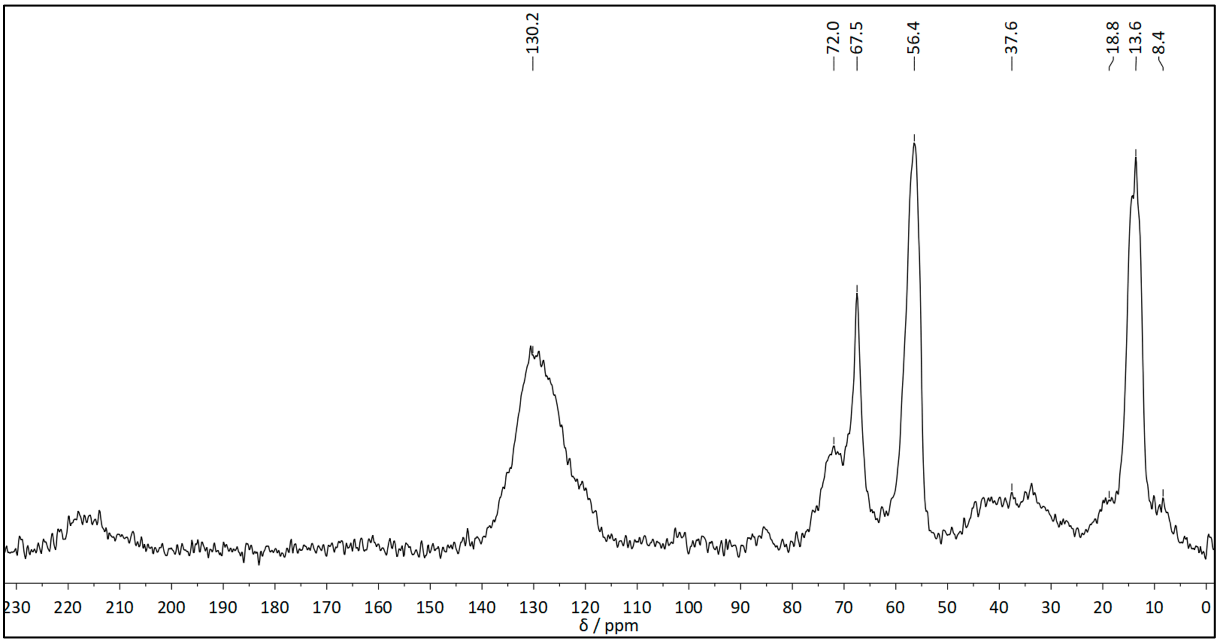

Figure S21. *^{13}C CP-MAS NMR spectrum of material* ***4a****.*

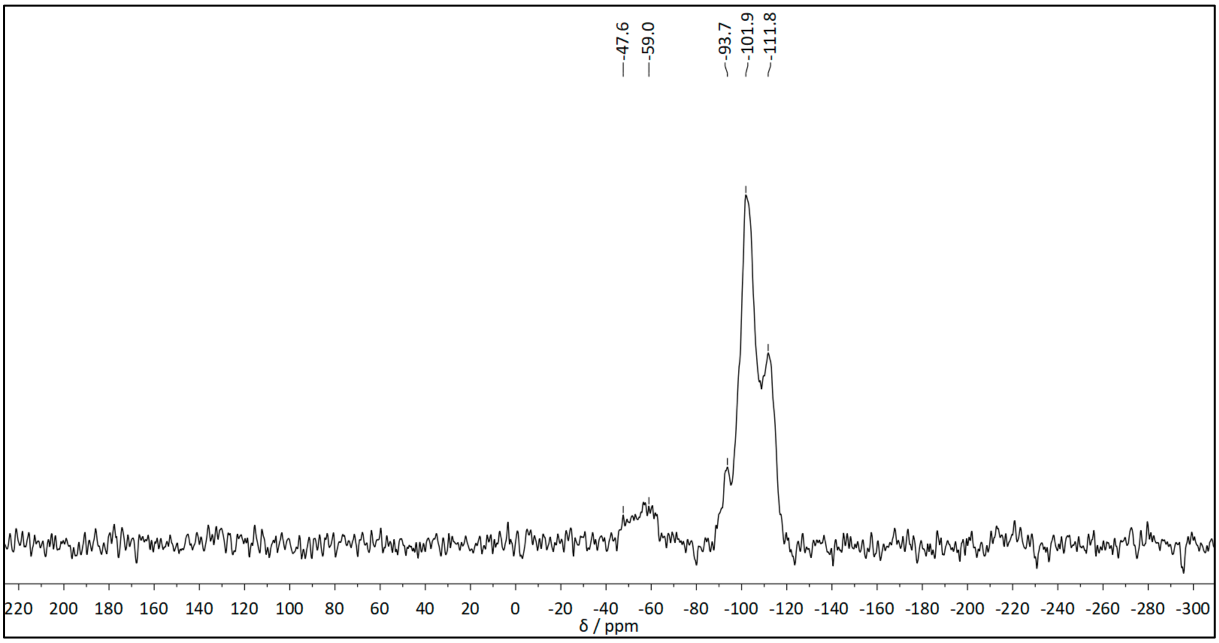

Figure S22. *^{29}Si CP-MAS NMR spectrum of material* ***4a****.*

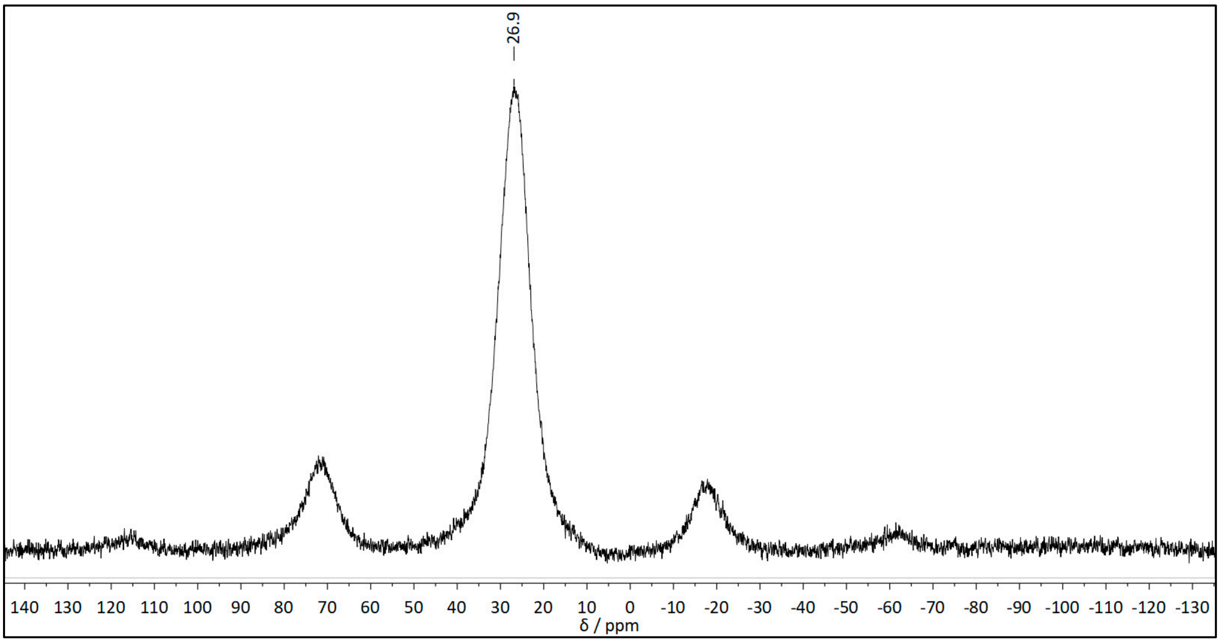

Figure S23. *^{31}P CP-MAS NMR spectrum of material **4a**.*

NMR spectra of material **4b**

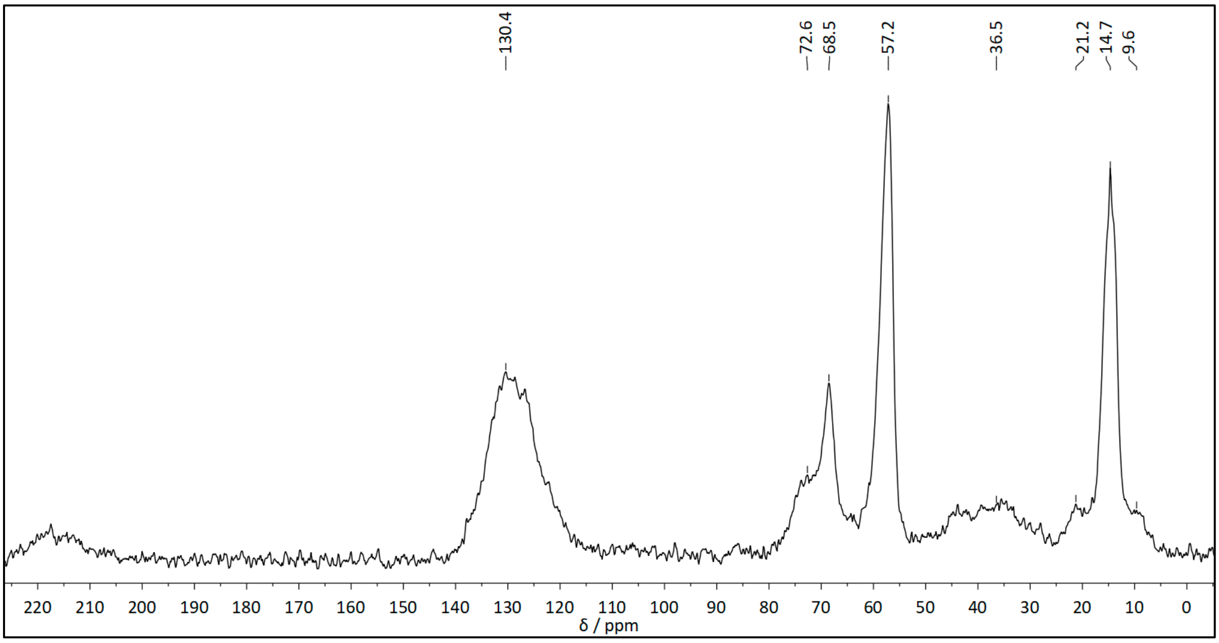

Figure S24. *^{13}C CP-MAS NMR spectrum of material **4b**.*

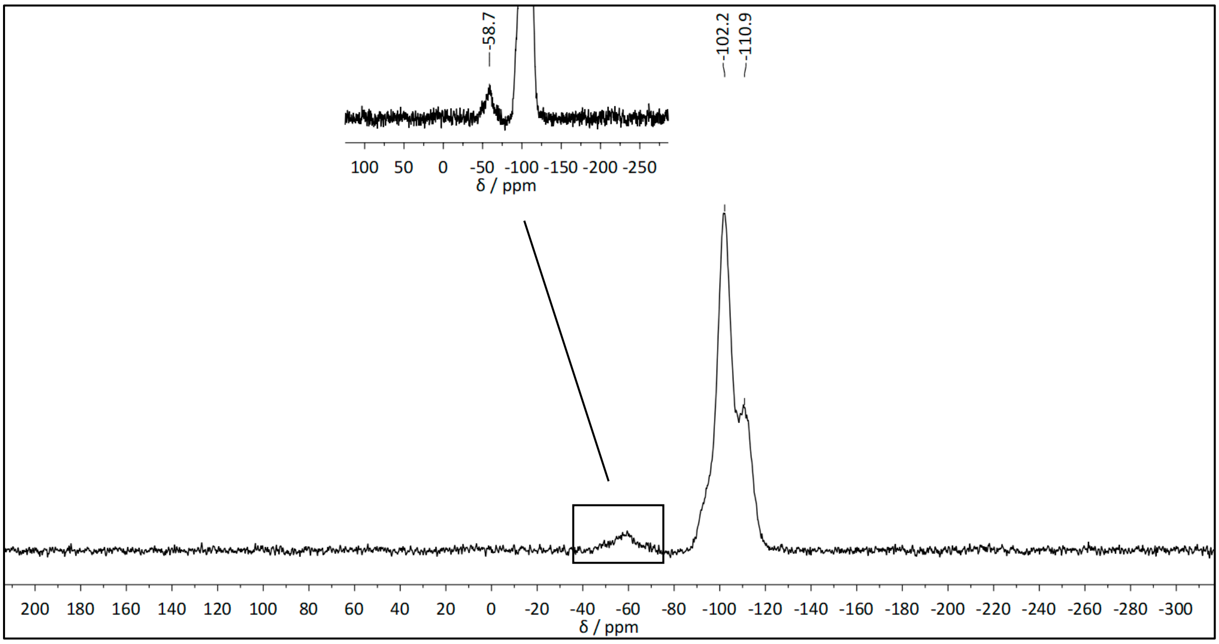

Figure S25. *^{29}Si CP-MAS NMR spectrum of material **4b**.*

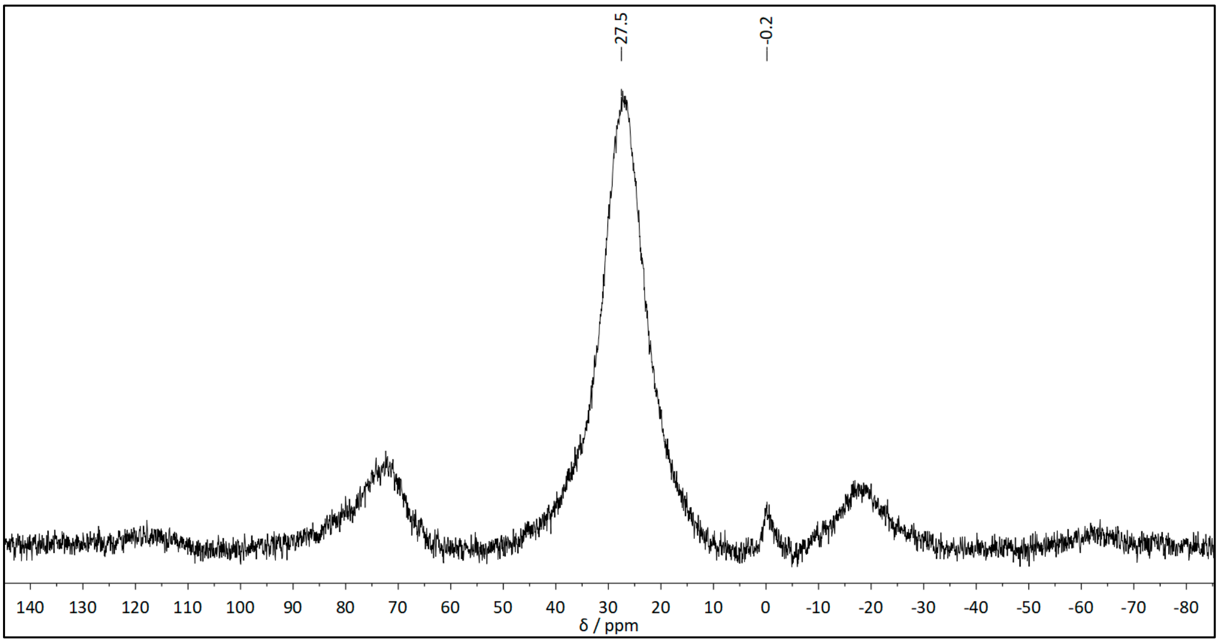

Figure S26. *^{31}P CP-MAS NMR spectrum of material **4b**.*

NMR spectra of material **5a**

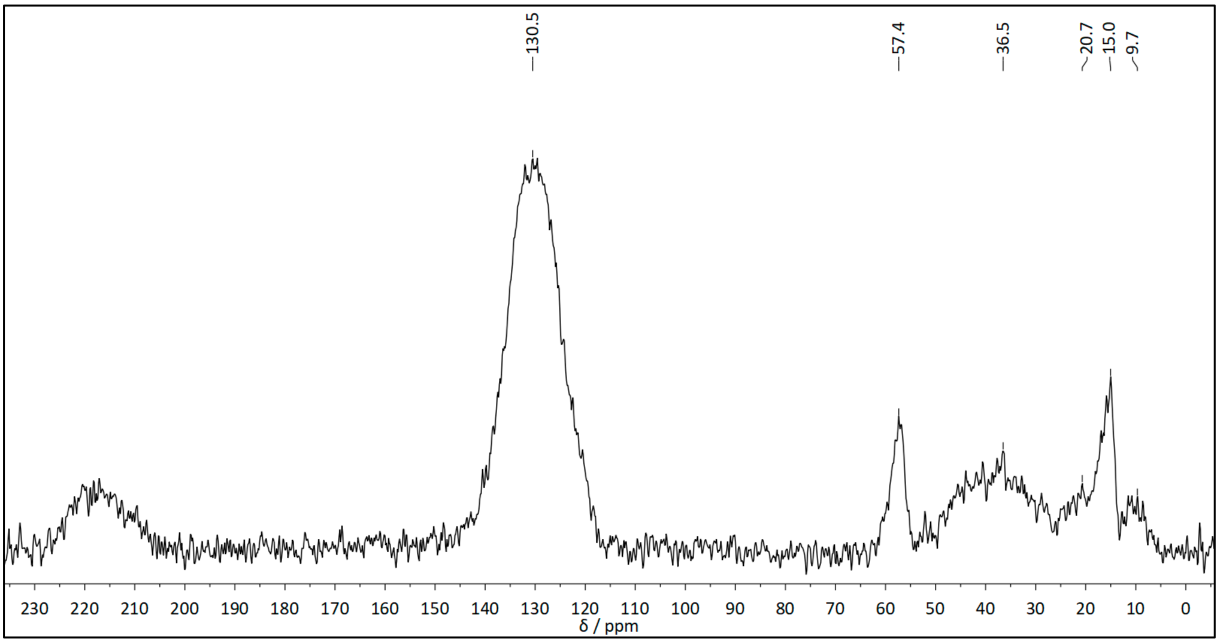

Figure S27. *^{13}C CP-MAS NMR spectrum of material* ***5a****.*

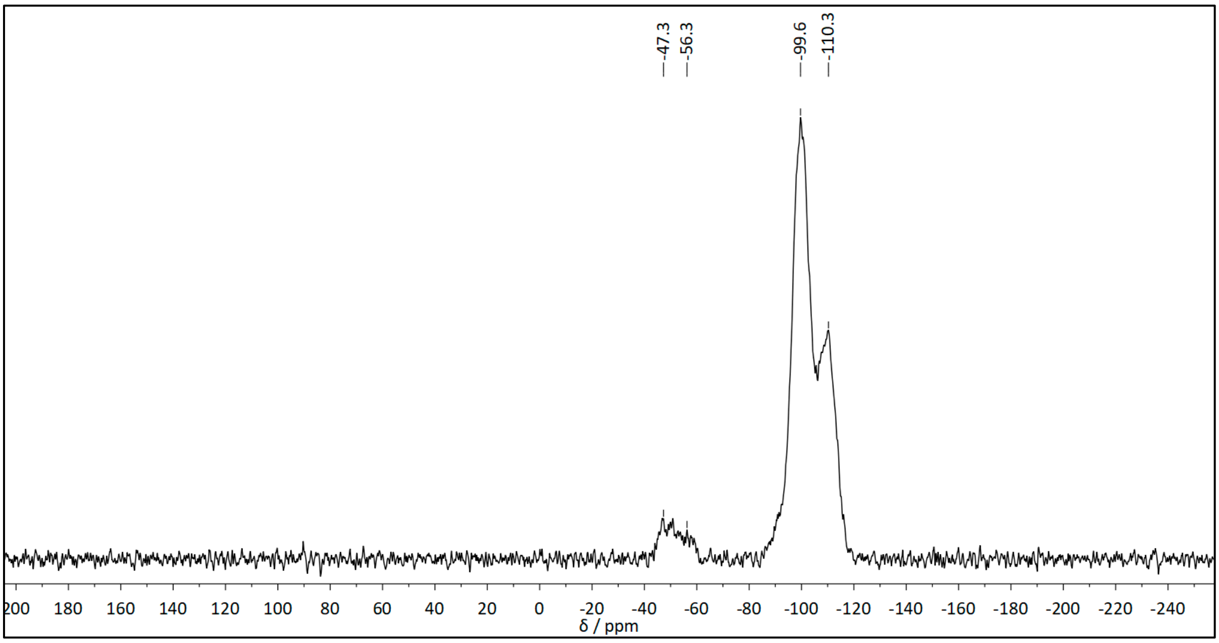

Figure S28. *^{29}Si CP-MAS NMR spectrum of material* ***5a****.*

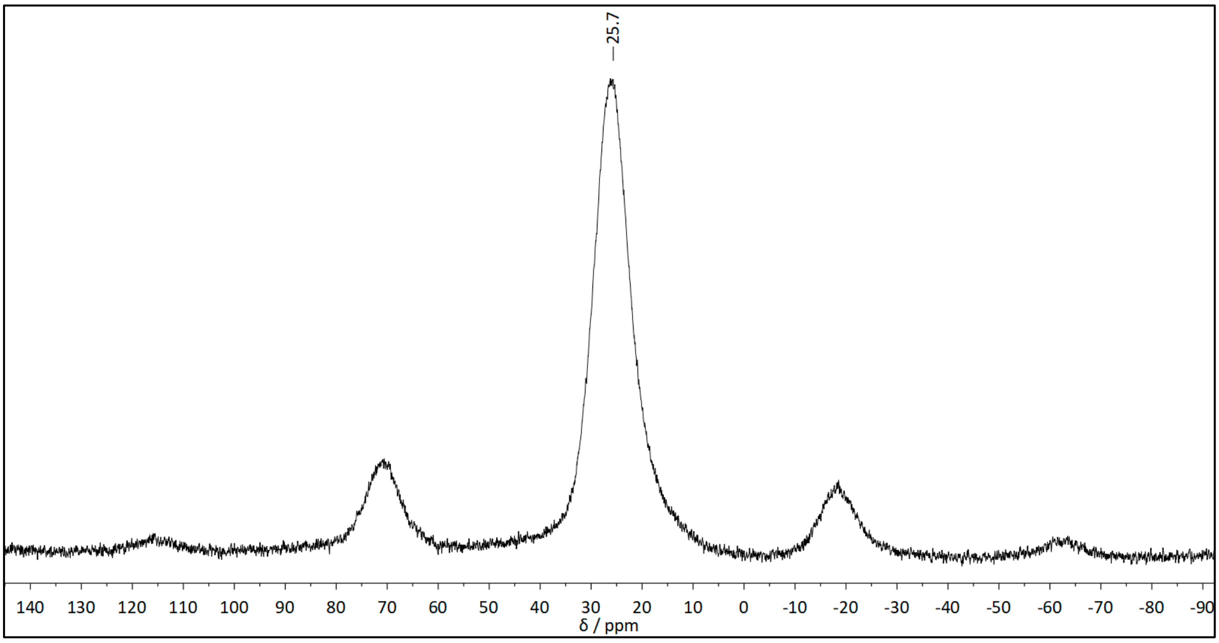

Figure S29. *^{31}P CP-MAS NMR spectrum of material* ***5a****.*

NMR spectra of material **5b**

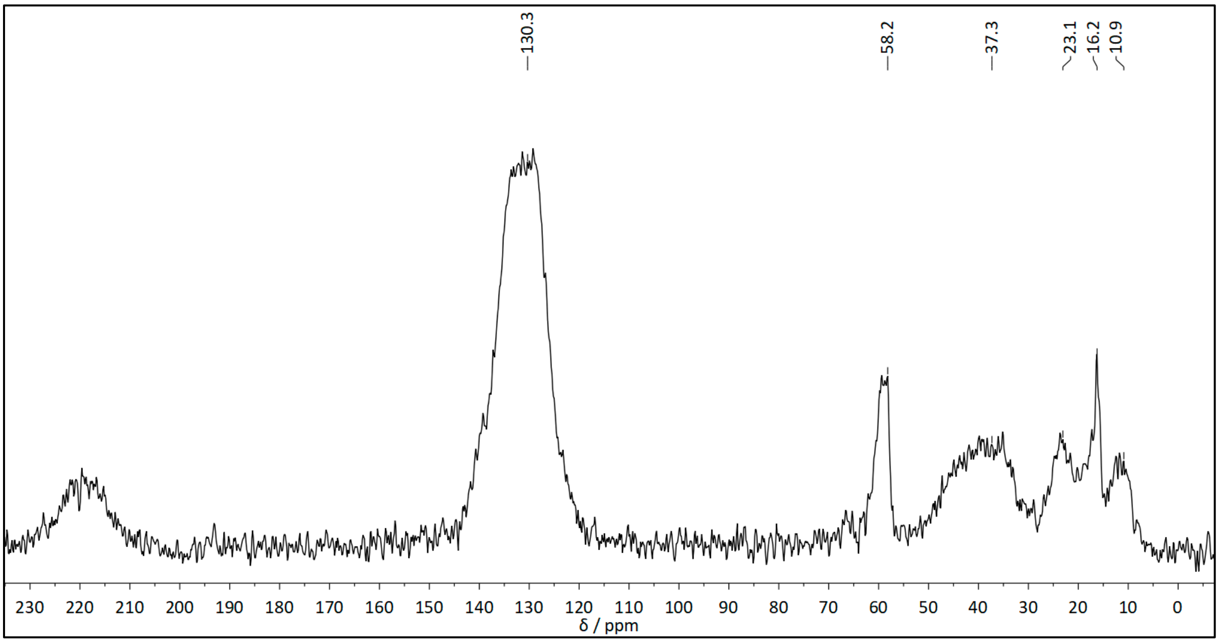

Figure S30. *^{13}C CP-MAS NMR spectrum of material* ***5b****.*

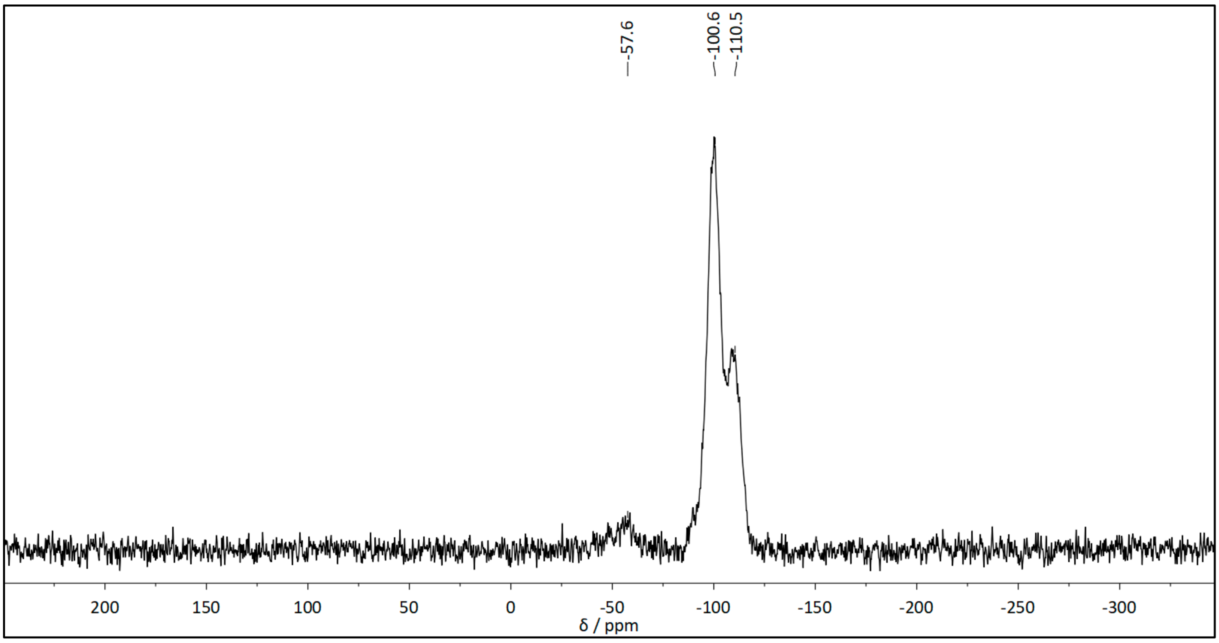

Figure S31. *^{29}Si CP-MAS NMR spectrum of material* ***5b****.*

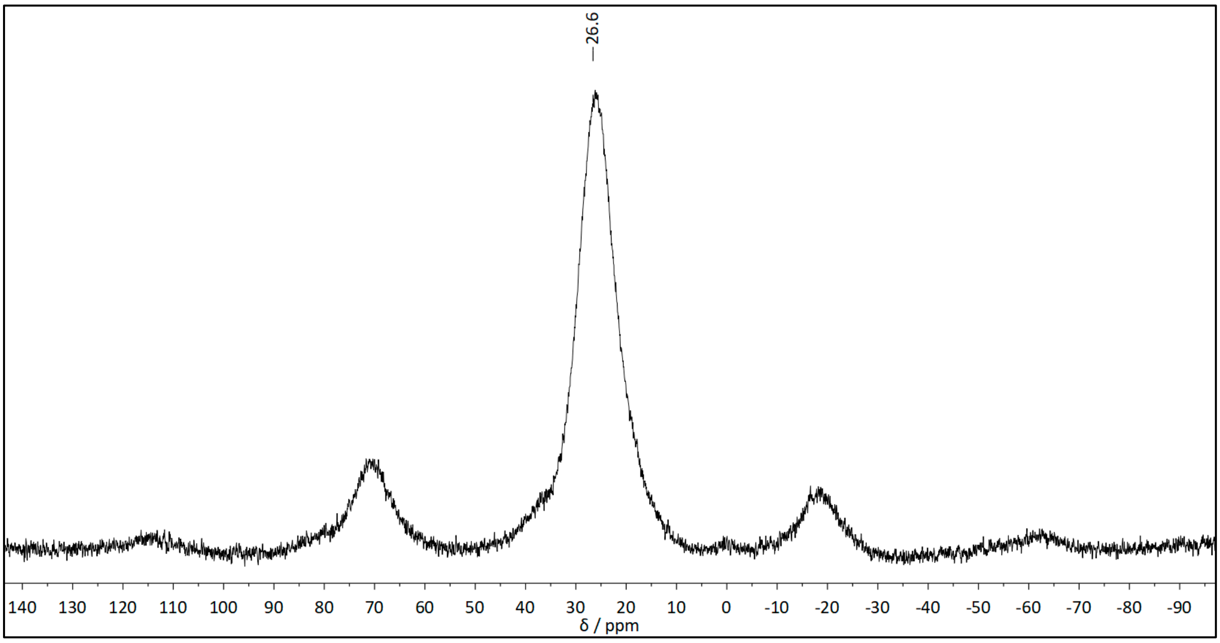

Figure S32. *^{31}P CP-MAS NMR spectrum of material* ***5b****.*

NMR spectra of material **6a**

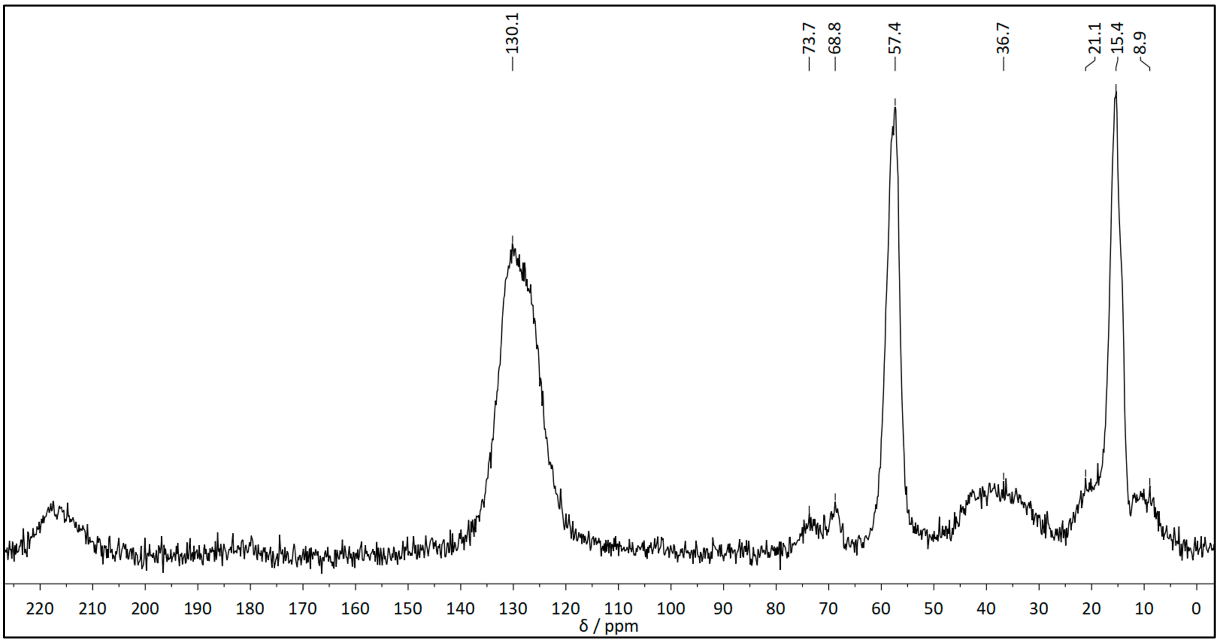

Figure S33. *^{13}C CP-MAS NMR spectrum of material* ***6a***.

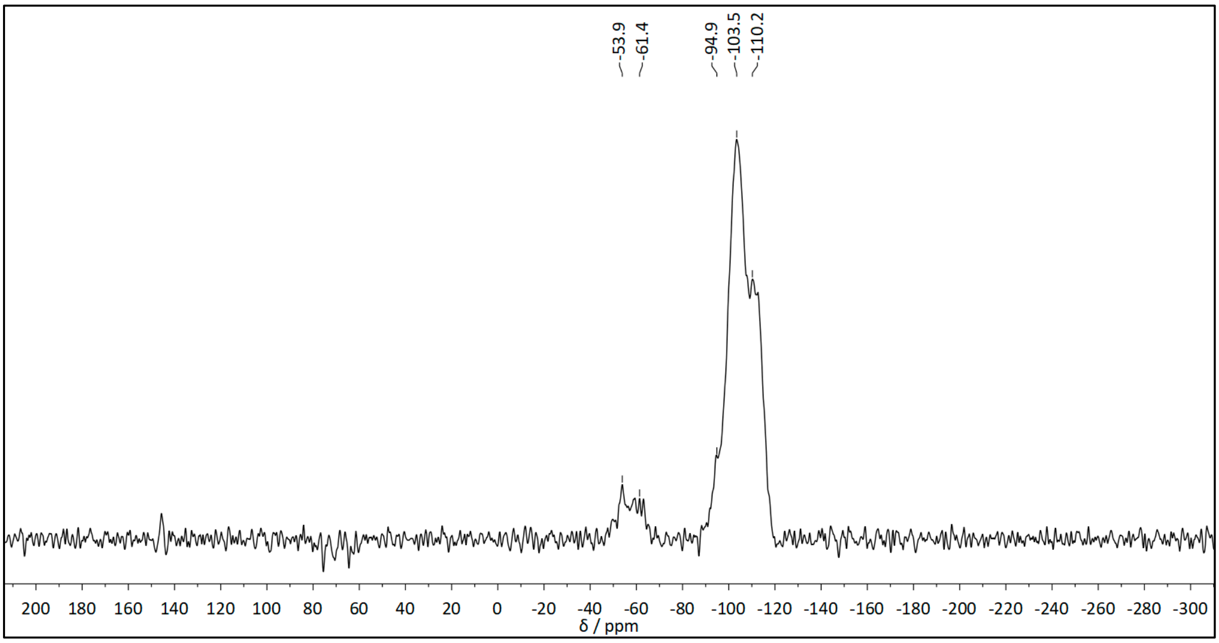

Figure S34. *^{29}Si CP-MAS NMR spectrum of material* ***6a***.

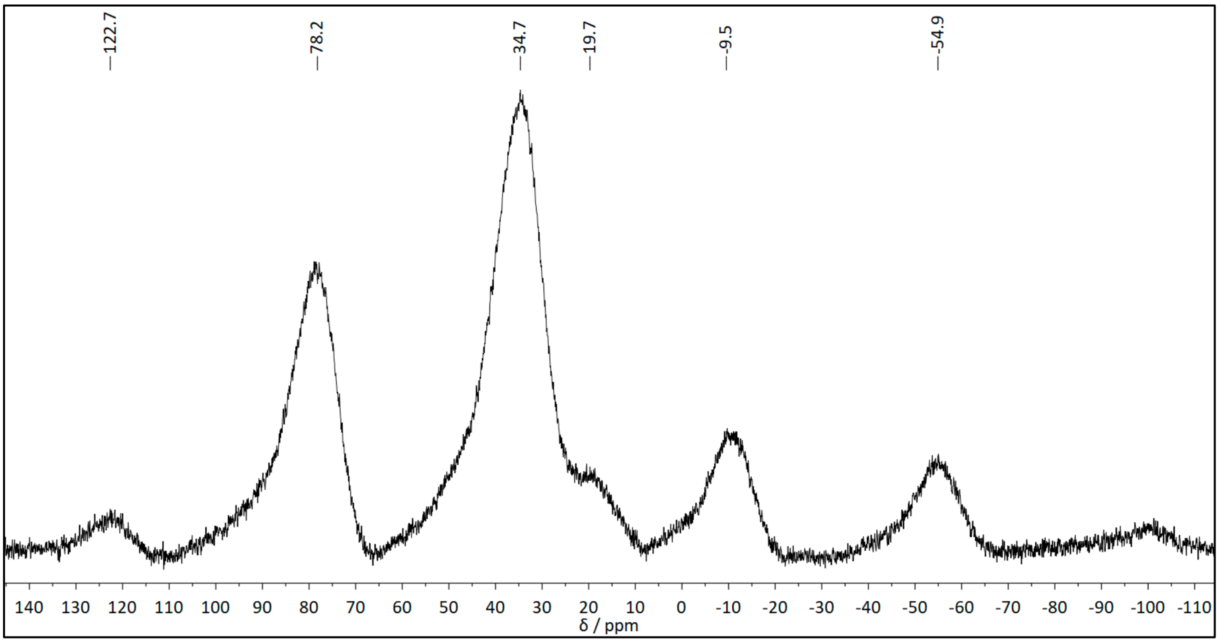

Figure S35. *^{31}P CP-MAS NMR spectrum of material* ***6a****.*

NMR spectra of material **6b**

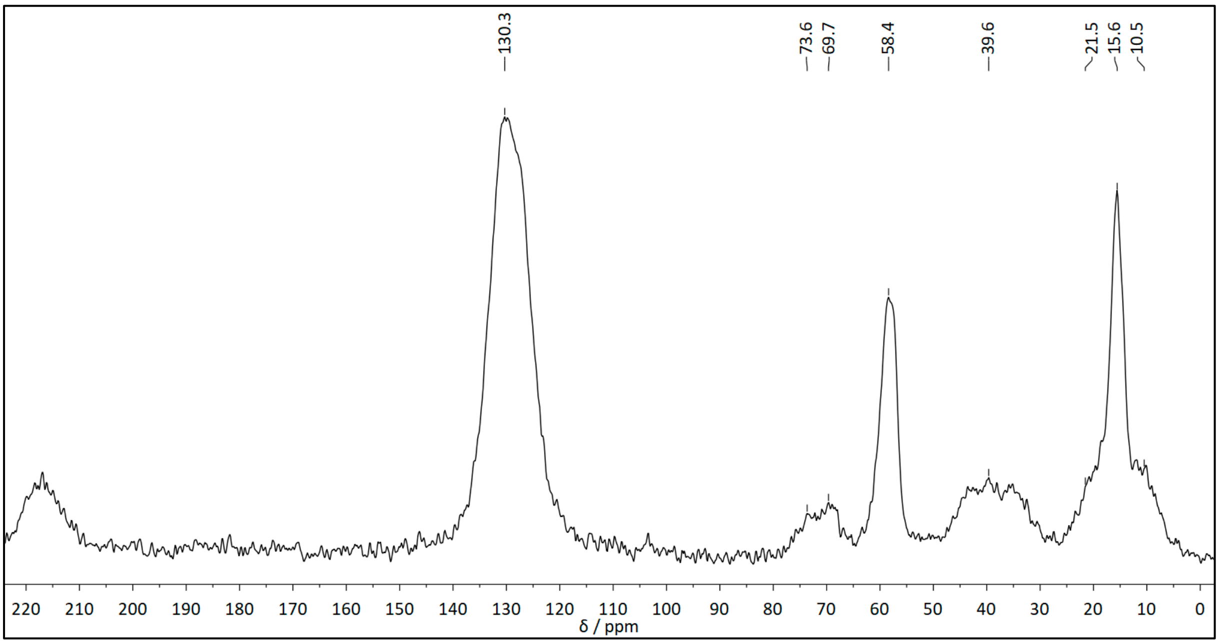

Figure S30. *^{13}C CP-MAS NMR spectrum of material* ***6b****.*

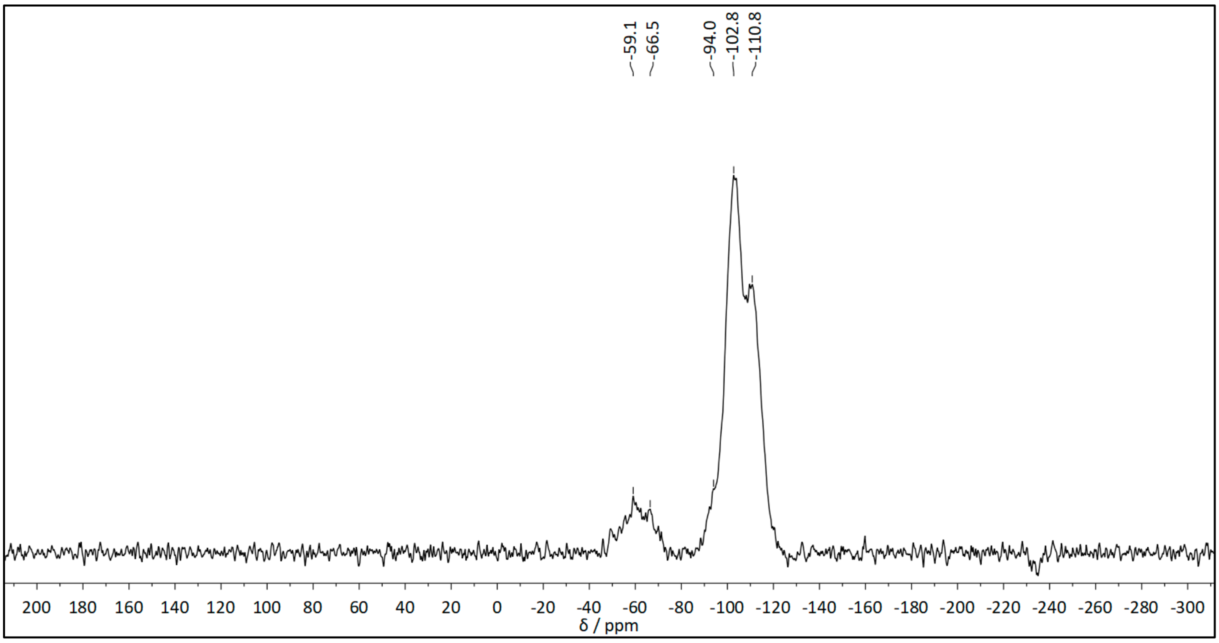

Figure S31. *^{29}Si CP-MAS NMR spectrum of material **6b**.*

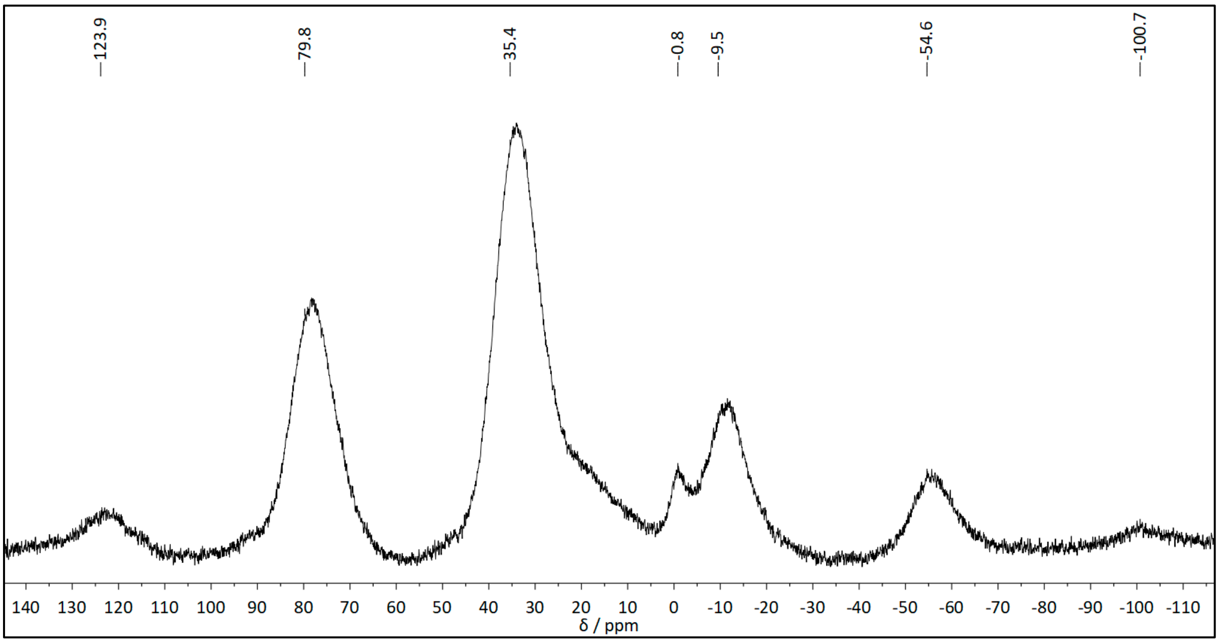

Figure S38. *^{31}P CP-MAS NMR spectrum of material **6b**.*

IR spectra

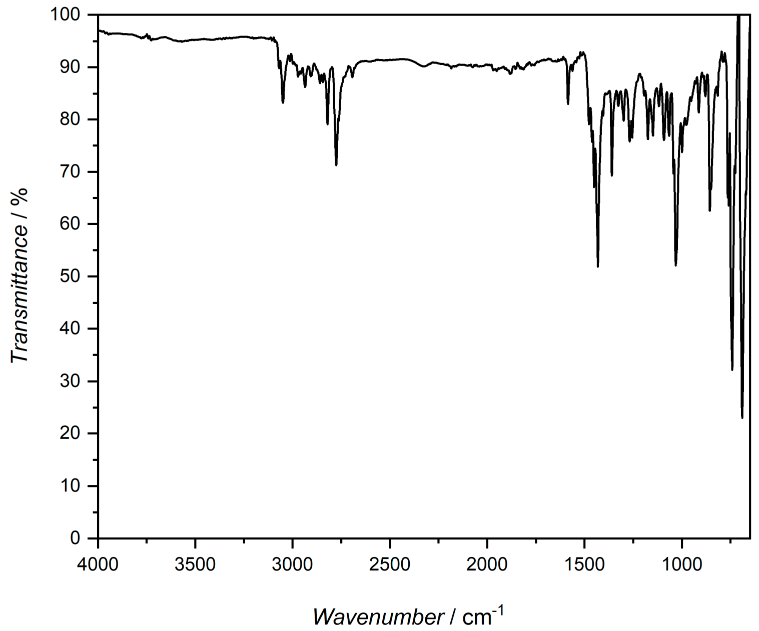

Figure S32. *FT-ATR-IR spectrum of 1-(2-(diphenylphosphino)phenyl)-N,N-dimethylmethanamine.*

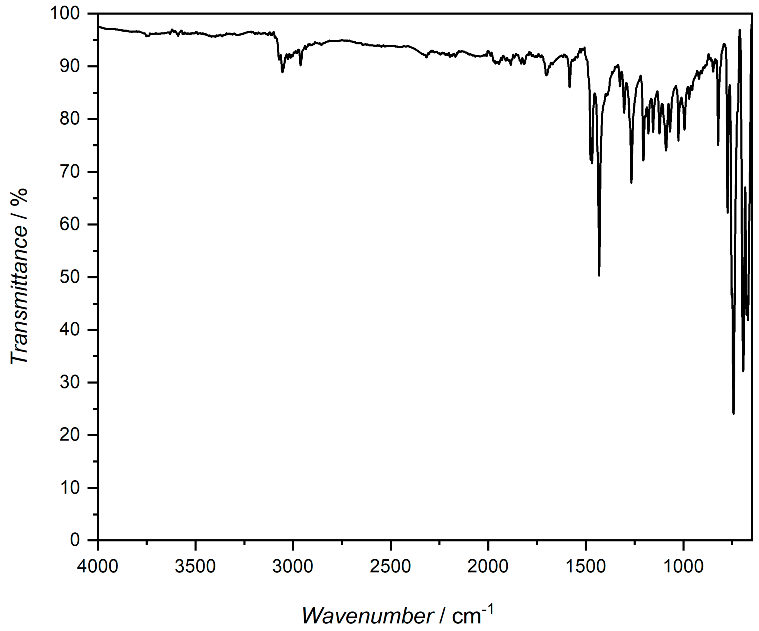

Figure S40. *FT-ATR-IR spectrum of (2-(chloromethyl)phenyl)diphenylphosphine.*

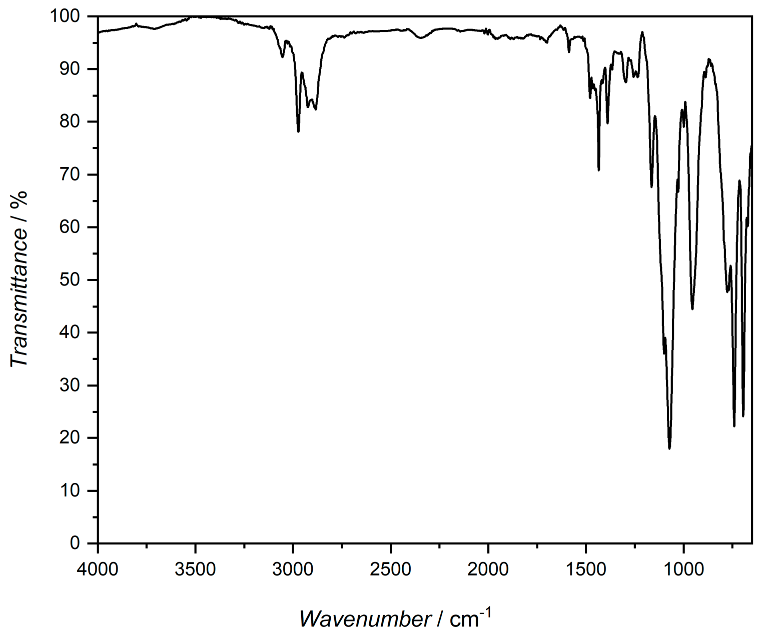

Figure S41. *FT-ATR-IR spectrum of ligand* ***1****.*

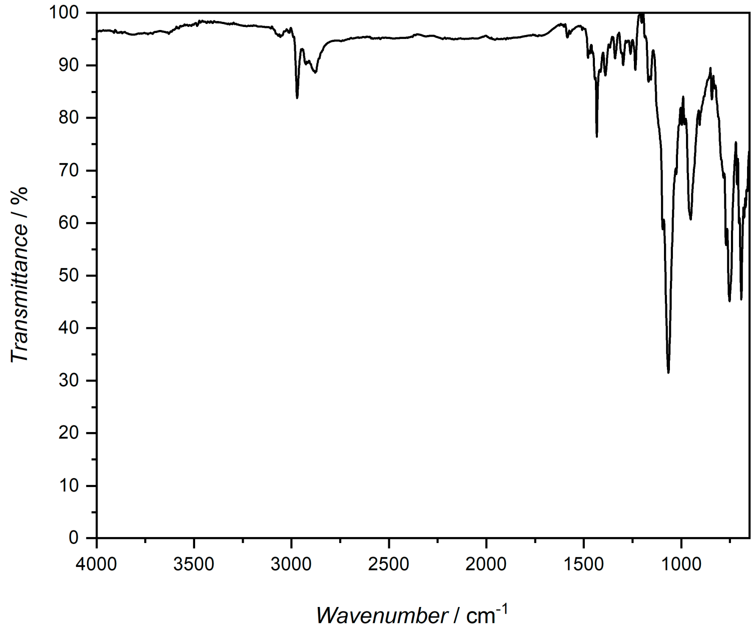

Figure S42. *FT-ATR-IR spectrum of complex* ***2****.*

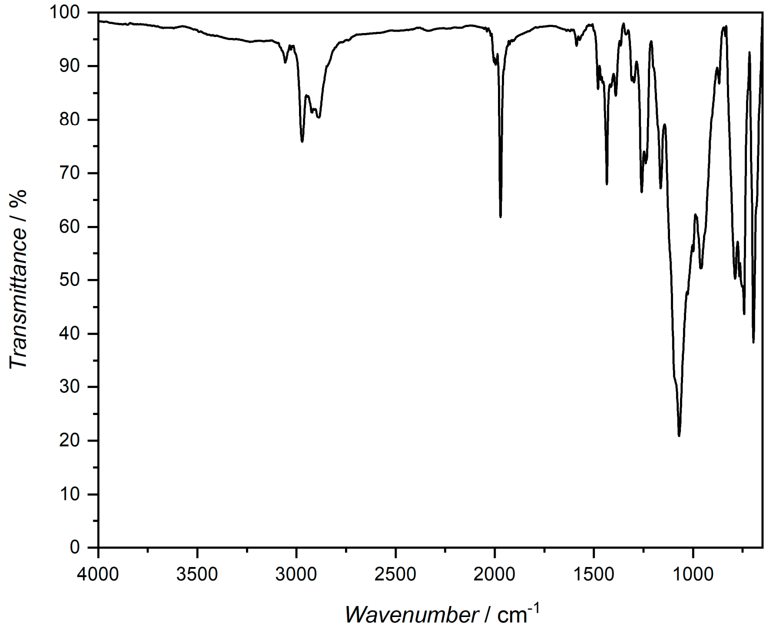

Figure S43. *FT-ATR-IR spectrum of the mixture of the rhodium complexes **3** and **3'**.*

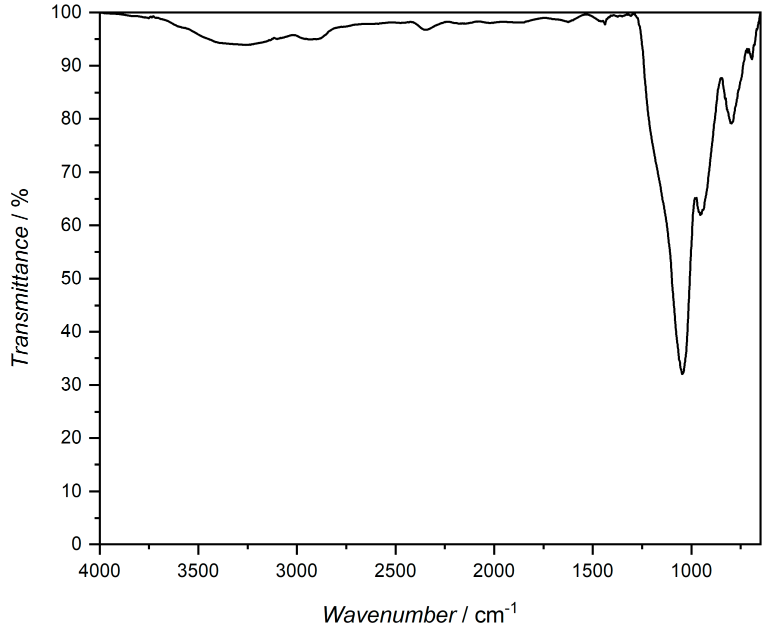

Figure S44. *FT-ATR-IR spectrum of material **4a**.*

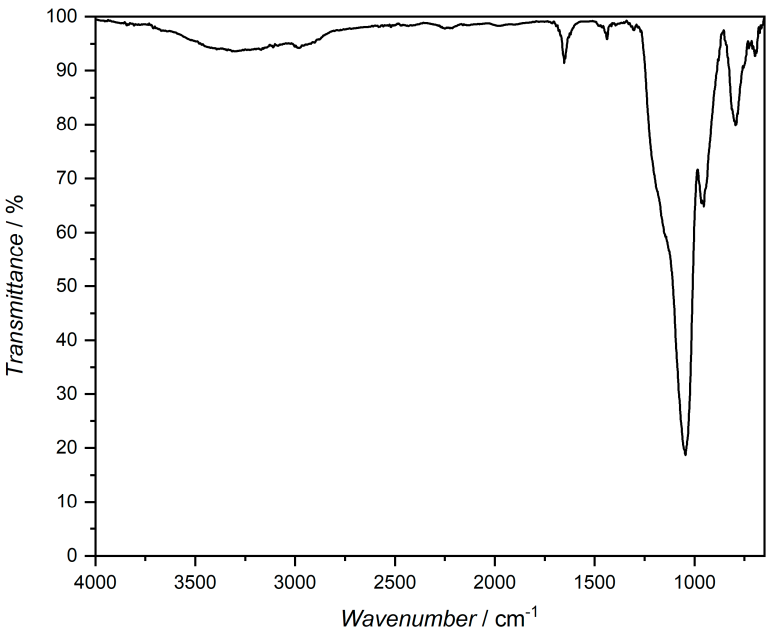

Figure S45. *FT-ATR-IR spectrum of material* ***4b***.

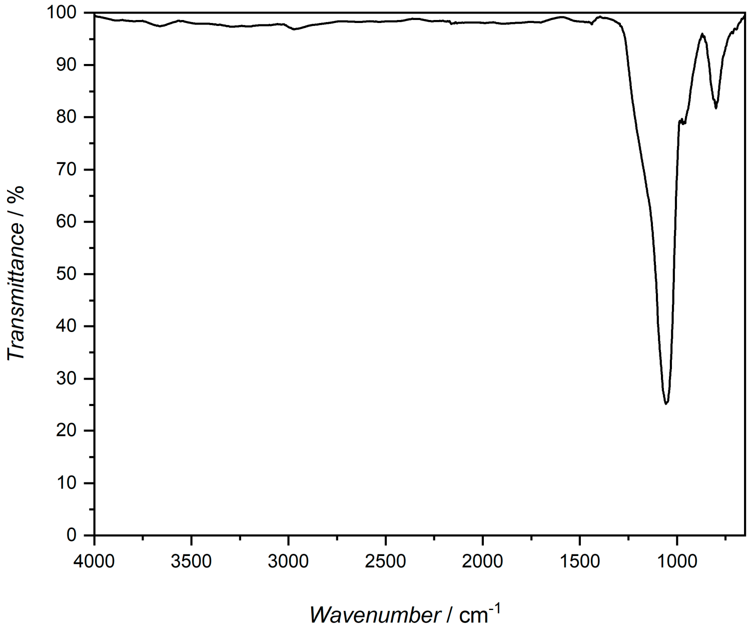

Figure S46. *FT-ATR-IR spectrum of material* ***5a***.

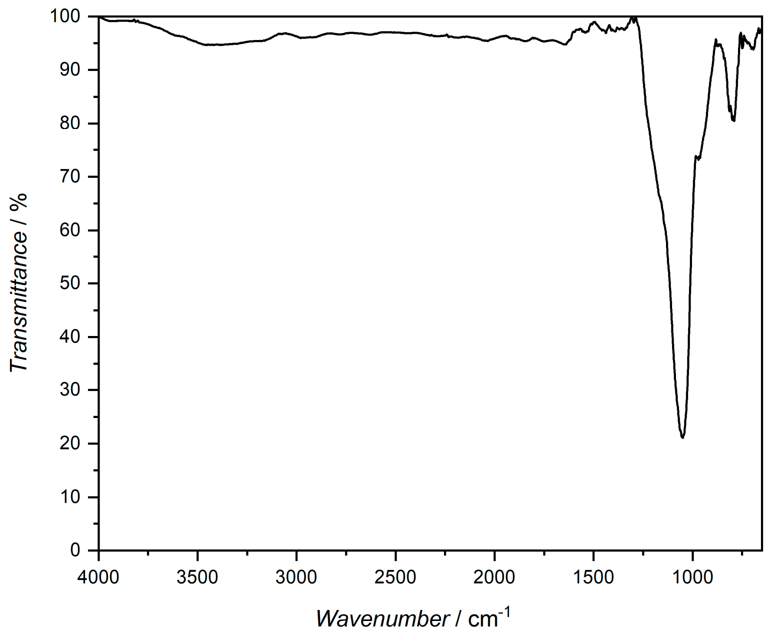

Figure S47. *FT-ATR-IR spectrum of material* ***5b***.

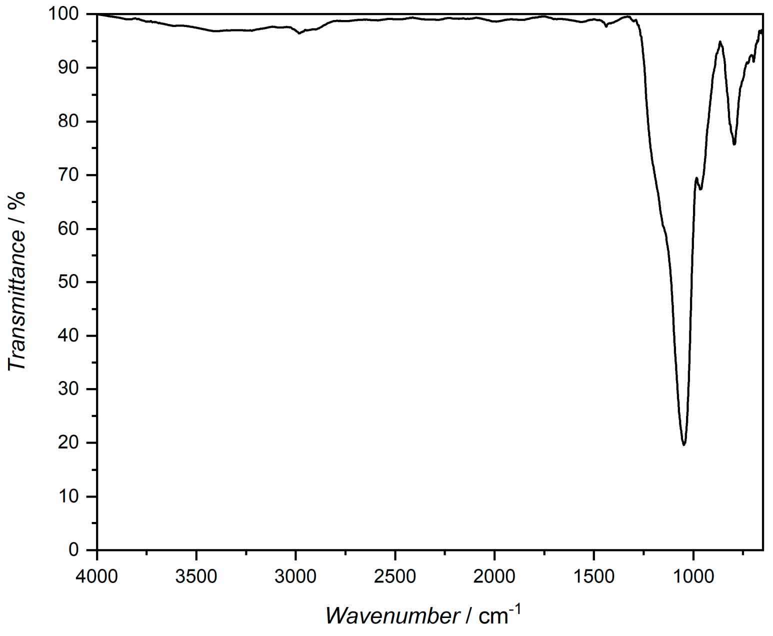

Figure S48. *FT-ATR-IR spectrum of material* ***6a***.

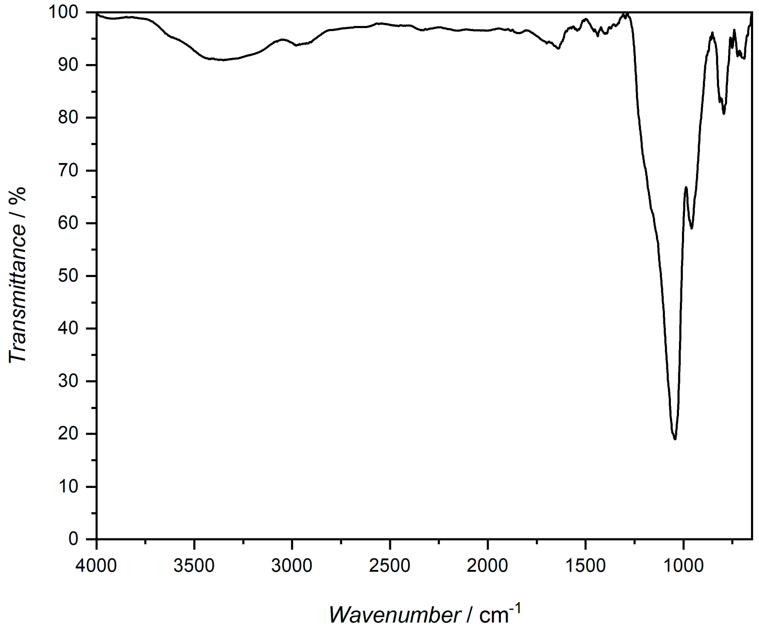

Figure S49. *FT-ATR-IR spectrum of material* ***6b***.

EDX data, XPS and TEM

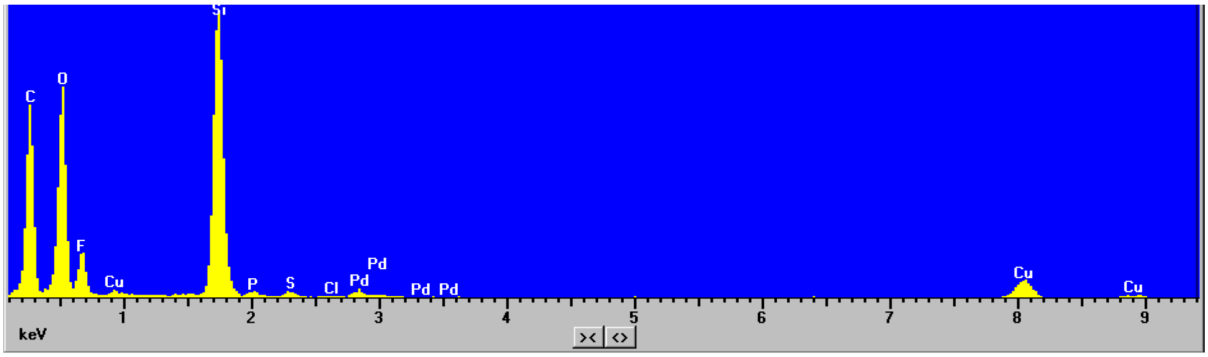

Figure S50. *EDX spectrum of material **4a**.*

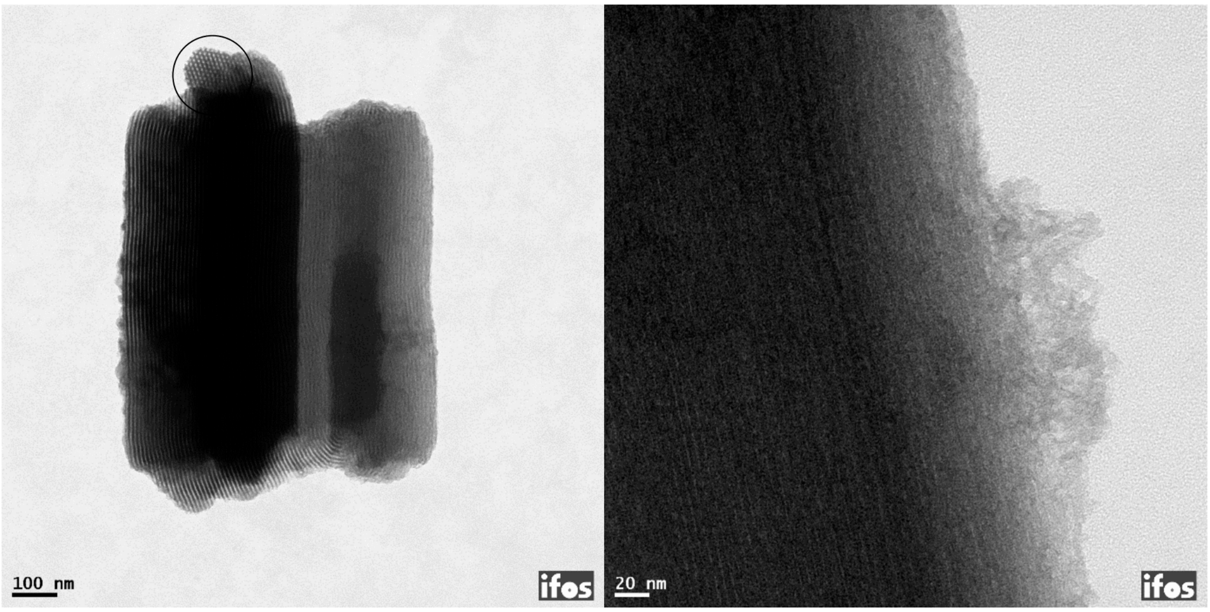

Figure S49+x. *EFTEM images of material **4a**; the hexagonal structure is clearly visible in the circle on the left image.*

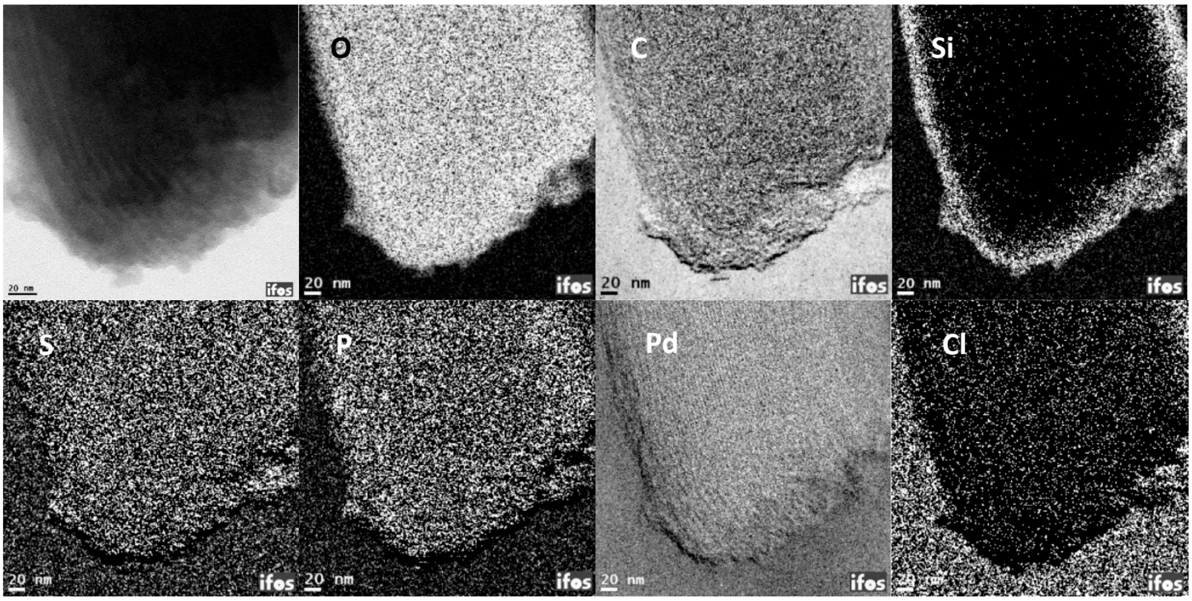

Figure S51. *EFTEM image (upper left image) and element mapping (TEM) of material **4a**; the mapping of palladium and chlorine is close to the noise level and thus not representative.*

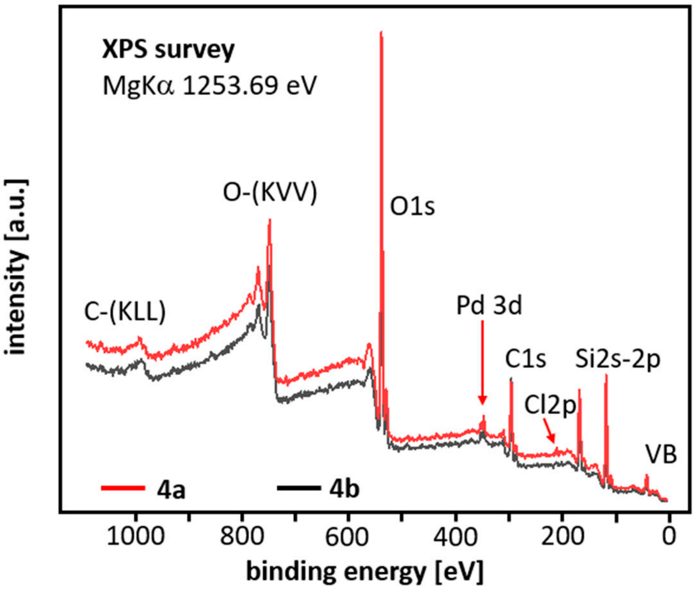

The unusually high oxygen concentration (O1s signal) can be explained by possible residual water in the matrix material itself. For the 4b complex, the chlorine Cl 2p signal is practically nonexistent (reduction).

Figure S52. *XPS survey spectra of materials **4a,b**.*

4a no hydrogenation

Area ratio [area units = au]

Pd 3d 5/2 total	1812.7 au	
Pd^{d+}	280.0 au	= 15.45 %
Pd(II)s	825.0 au	= 45.50%
Pd(II)b	707.7 au	= 39.04%
Pd (II) total		= 84.54%

4b hydrogenation

Pd 3d 5/2 total	2917.53 a.u.	
$Pd^{\delta+}$	280.0 a.u.	= 30.85 %
Pd^{0}	500.0 a.u.	= 17.13%
$Pd^{\delta+}$ Pd^{0} total		= 47.98%
Pd(II)s	1058.63 a.u.	= 36.29%
Pd(II)b	458.9 a.u.	= 15.73%
Pd (II) total		= 51.59%

4b recycled.

Pd 3d 5/2 total	2201.4 a.u.	
$Pd^{\delta+}$	280.0 a.u.	= 36.34 %
Pd^{0}	500.0 a.u.	= 12.07%
$Pd^{\delta+}$ Pd^{0} total		= 48.41%
Pd(II)s	967,2 a.u.	= 43.94%
Pd(II)b	168,54 a.u.	= 7.65%
Pd (II) total		= 51.59%

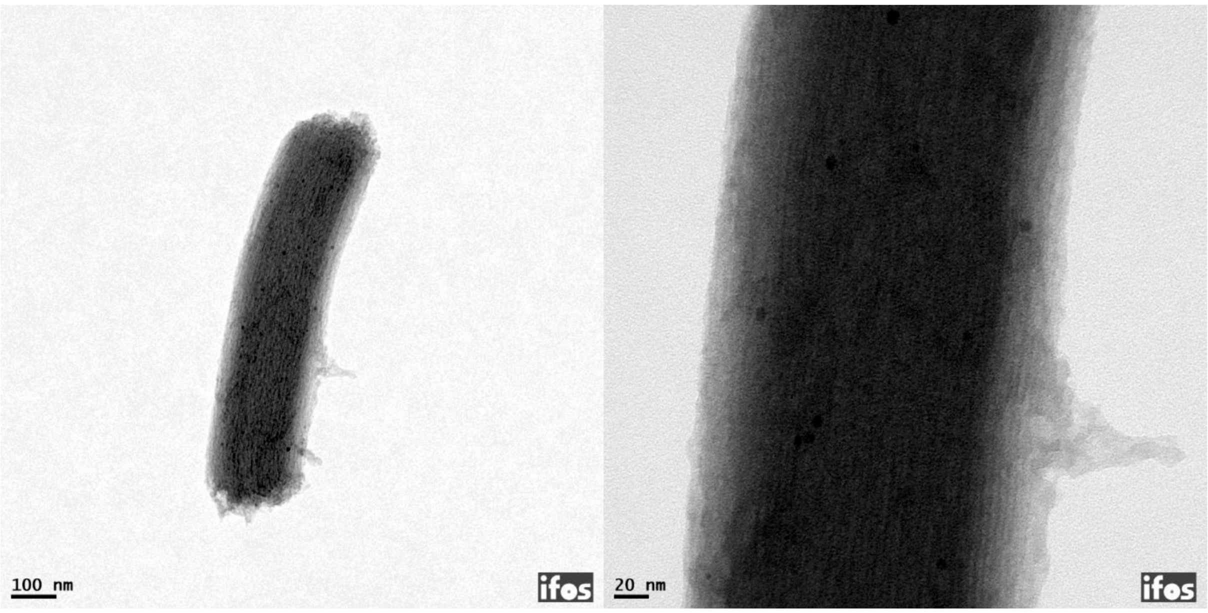

Figure S53. *EFTEM images of material **4b**.*

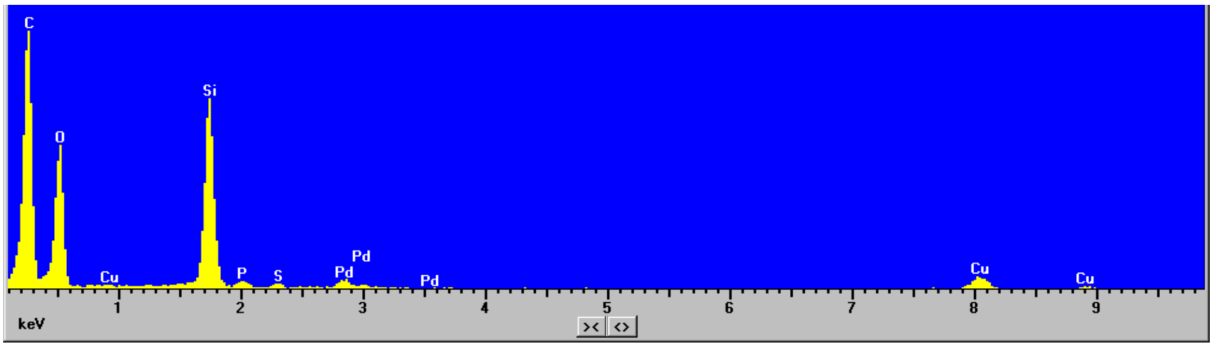

Figure S54. *EDX spectrum of material* ***5a****.*

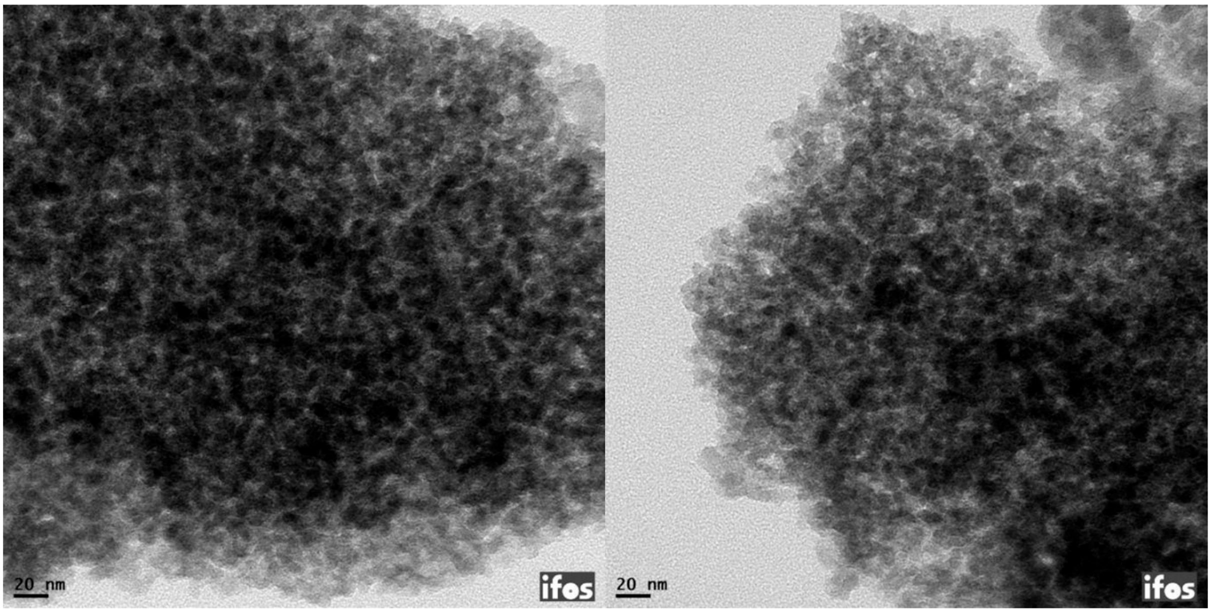

Figure S55. *EFTEM images of material* ***5a****.*

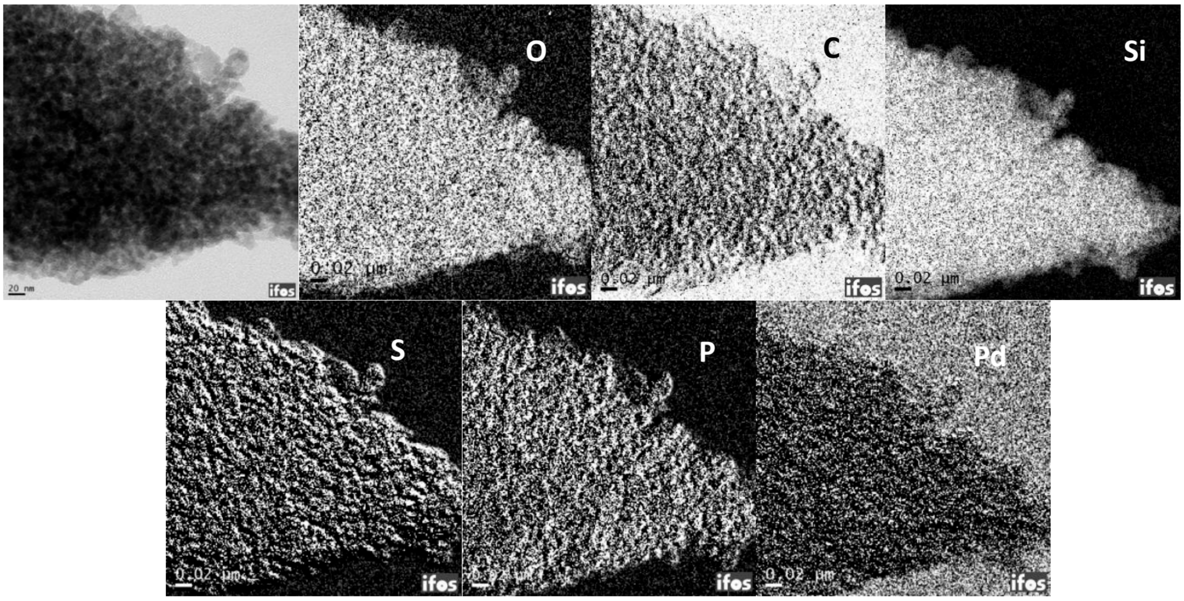

Figure S56. *EFTEM image (upper left image) and element mapping (TEM) of material* ***5a****; the mapping of palladium is close to the noise level and thus not representative.*

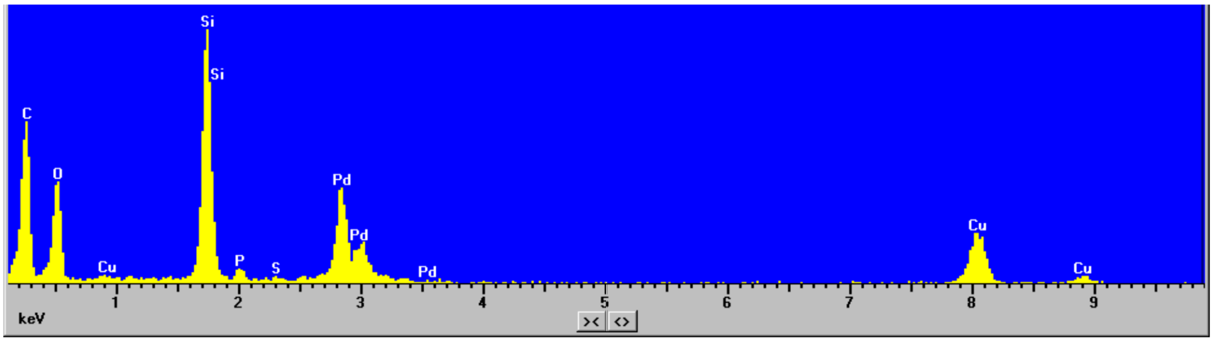

Figure S57. *EDX spectrum of material* ***5b****.*

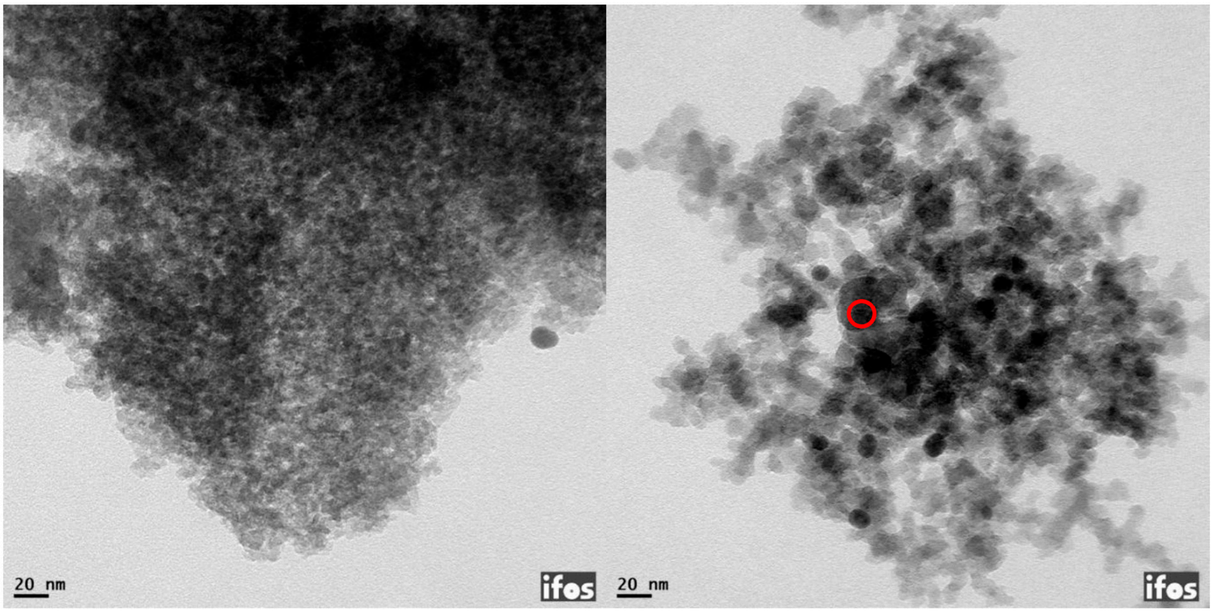

Figure S58. *EFTEM images of material* ***5b****.*

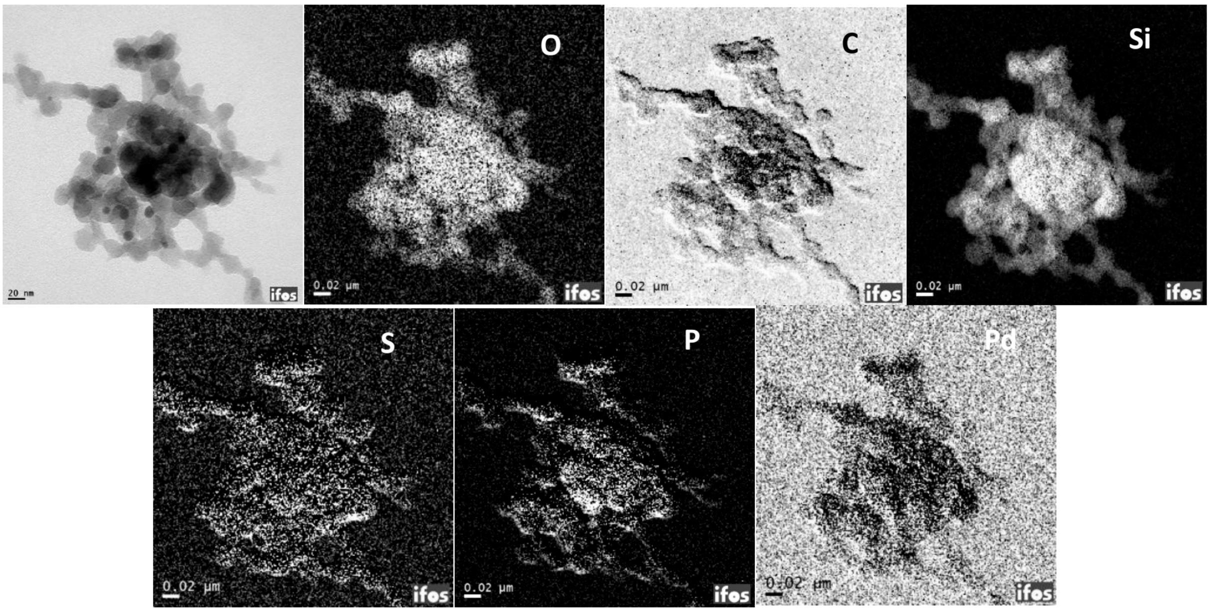

Figure S59. *EFTEM image (upper left image) and element mapping (TEM) of material* ***5b****; the mapping of palladium is close to the noise level and thus not representative.*

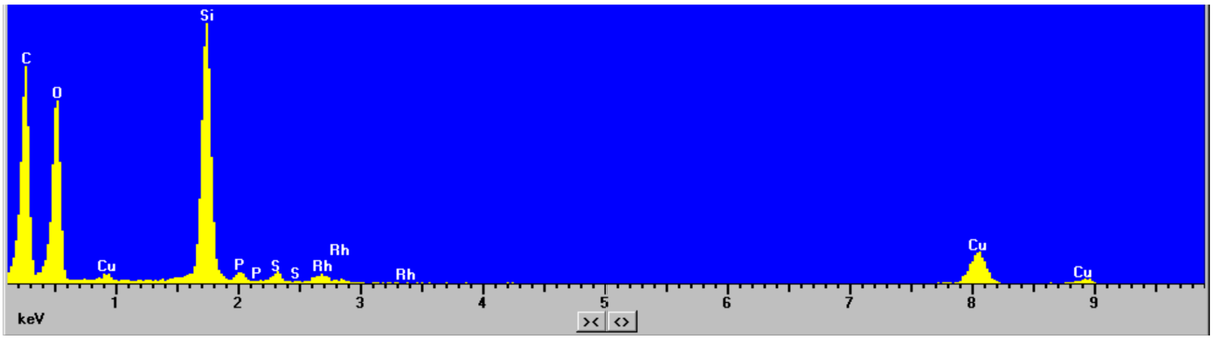

Figure S60. *EDX spectrum of material* ***6a****.*

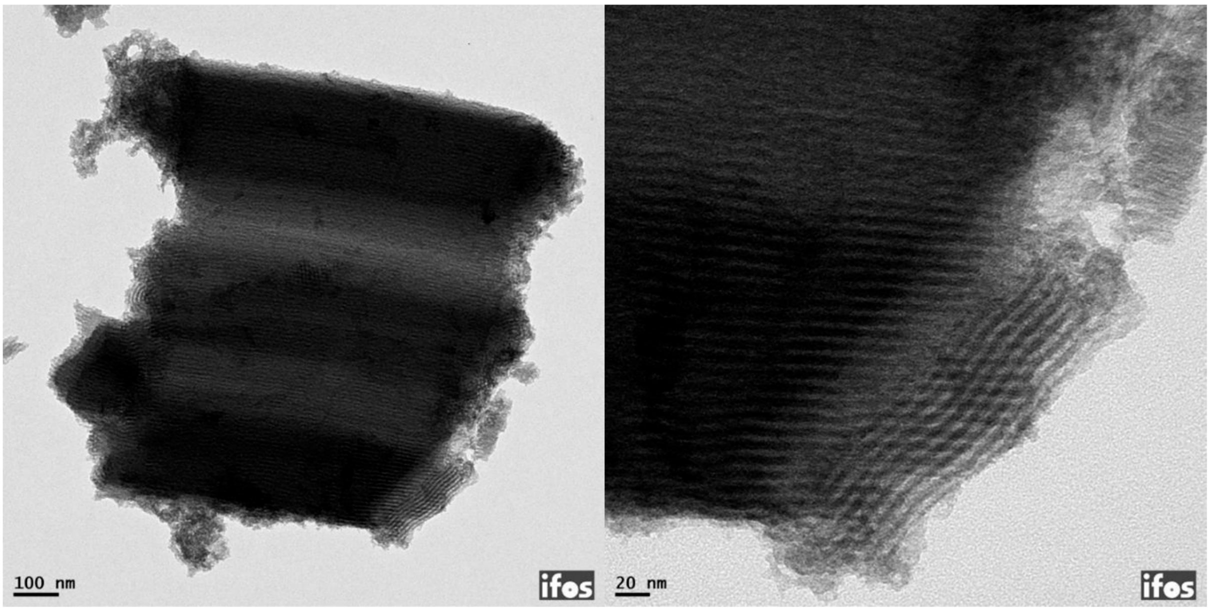

Figure S61. *EFTEM images of material* ***6a****.*

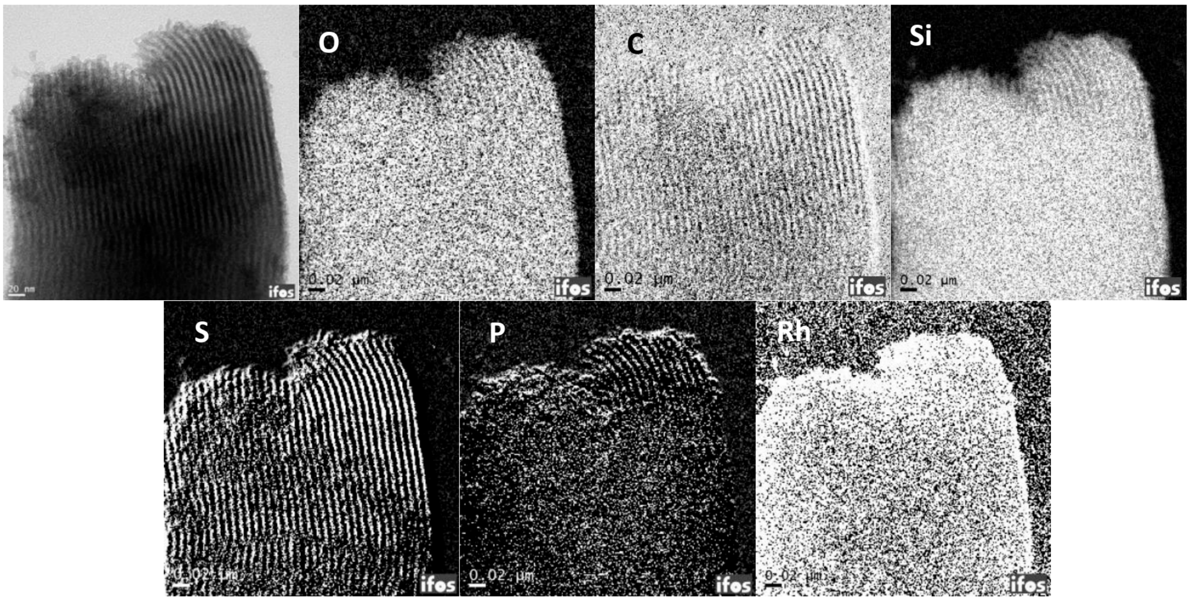

Figure S62. *EFTEM image (upper left image) and element mapping (TEM) of material* ***6a****; the mapping of rhodium is close to the noise level and thus not representative.*

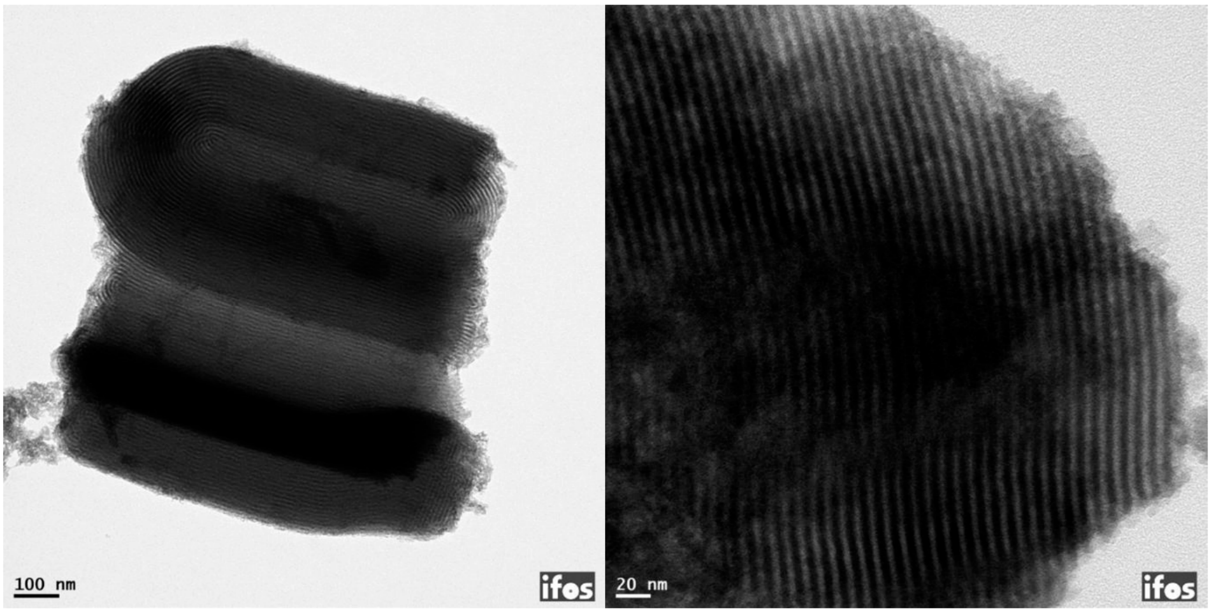

Figure S63. *TEM images of material* ***6b****.*

SEM images

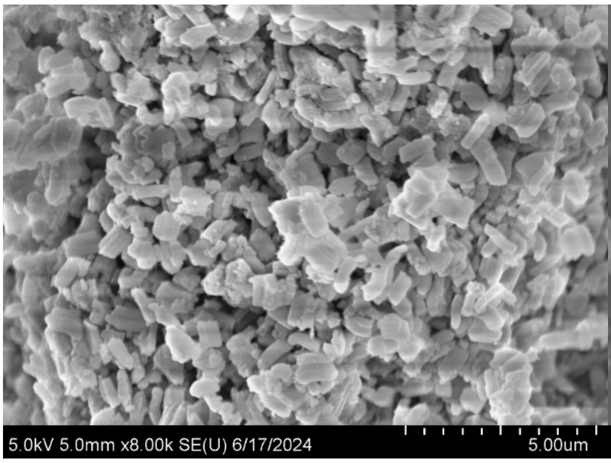

Figure S64. *SEM image of* ***4a***.

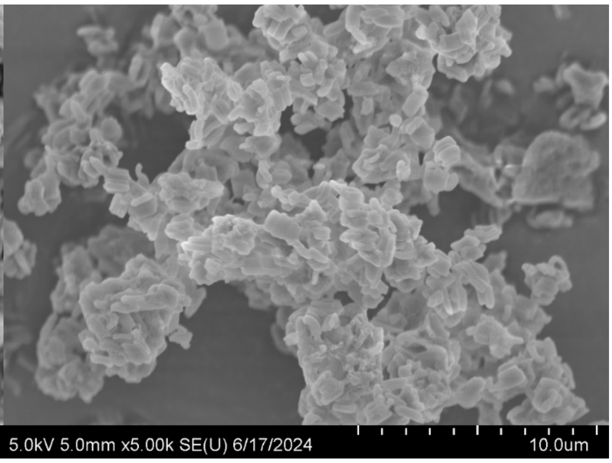

Figure S65. *SEM image of* ***4b***.

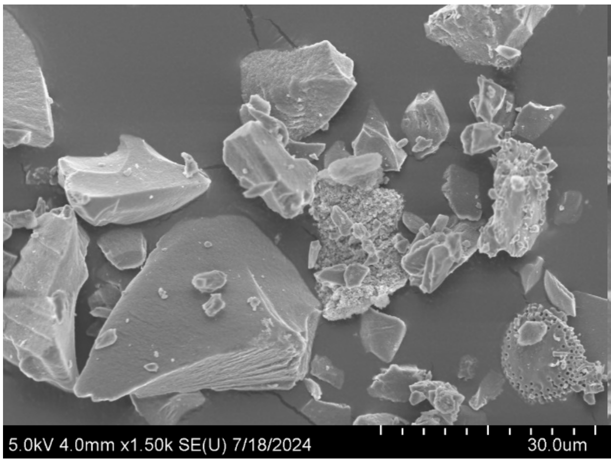

Figure S66. *SEM image of* ***5a***.

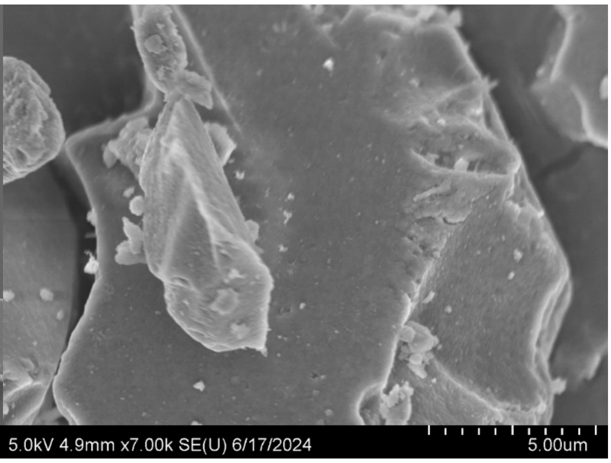

Figure S67. *SEM image of* ***5b***.

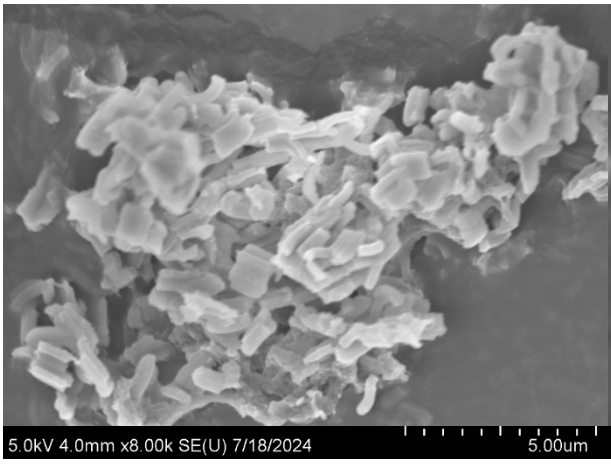

Figure S68. *SEM image of* ***6a***.

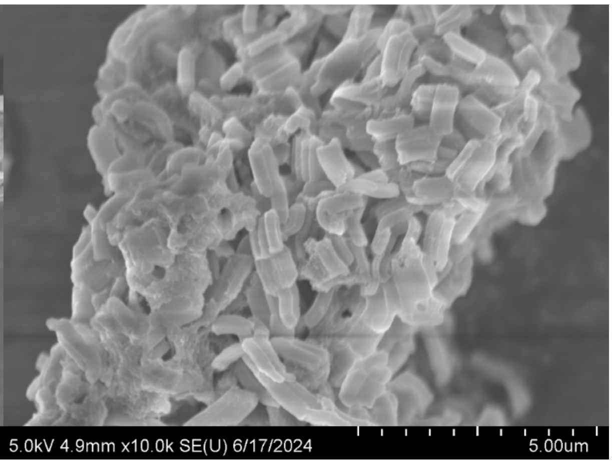

Figure S69. *SEM image of* ***6b***.

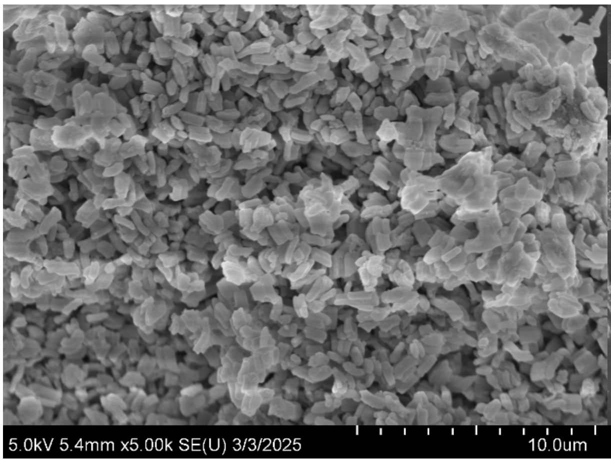

Figure S70. *SEM image of tenfold recycled catalyst* ***4b***.

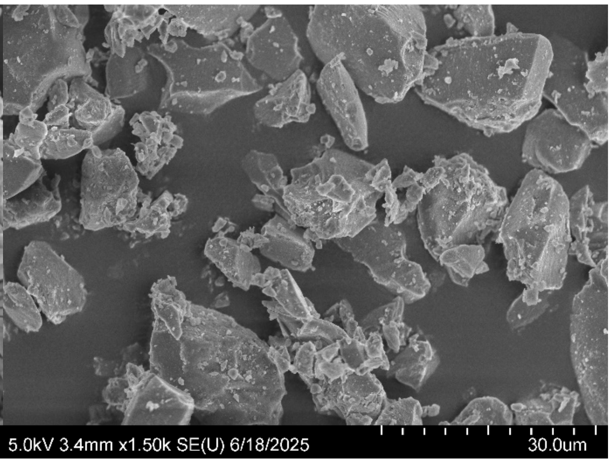

Figure S71. *SEM image of tenfold recycled catalyst* ***5b***.

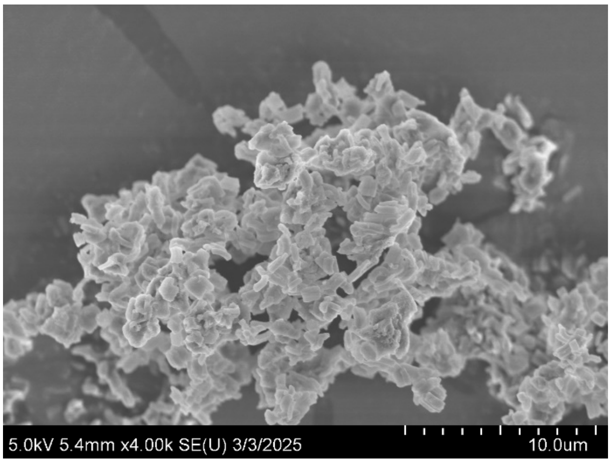

Figure S72. *SEM image of tenfold recycled catalyst* ***6b***.

Recycling studies

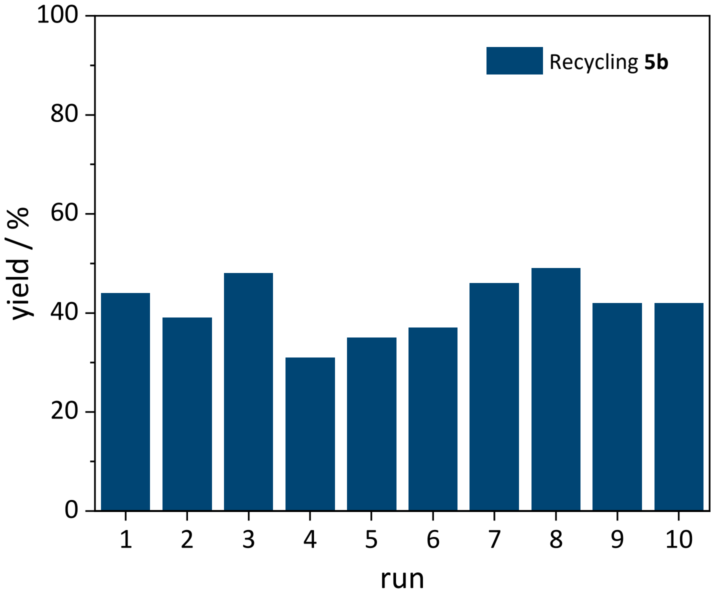

Figure S73. *Recycling of **5b** over ten cycles; reaction parameters: r.t., 1 bar* H_2*, 1 mmol of 2-cyclohexen-1-one, cyclohexane (2 ml), 1.2 mol-% of palladium, 1 h.*

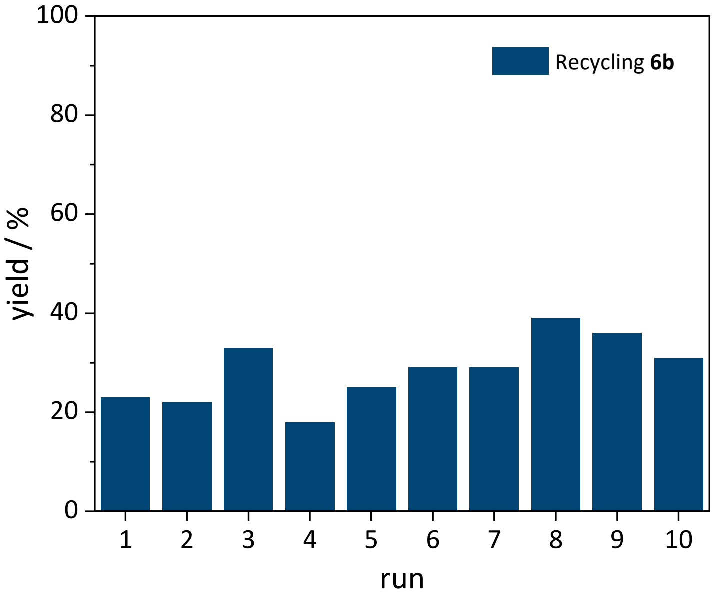

Figure S74. *Recycling of **6b** over ten cycles; reaction parameters: r.t., 1 bar* H_2*, 1 mmol of 2-cyclohexen-1-one, cyclohexane (2 ml), 1.2 mol-% of rhodium, 3 h.*
